# Decoding the chemical space of fast-ion conductors via a descriptor-guided transfer learning framework

**DOI:** 10.1126/sciadv.aee4959

**Published:** 2026-08-07

**Authors:** Zhilong Wang, Fengqi You

**Affiliations:** ^1^ Cornell University AI for Science Institute, Cornell University, Ithaca, NY 14853, USA.; ^2^ College of Engineering, Cornell University, Ithaca, NY 14853, USA.; ^3^ Robert Frederick Smith School of Chemical and Biomolecular Engineering, Cornell University, Ithaca, NY 14853, USA.

## Abstract

Fast-ion conductors (FICs) are key components for next-generation high-performance batteries, yet predicting ion mobility remains challenging because of the unclear transport mechanisms. This difficulty is further compounded by experimental datasets that lack precise crystal structures. Here, we present a descriptor-guided transfer learning framework, named IonNet, to predict ion mobility for compounds, regardless of structure accessibility. IonNet adopts a multichannel subnetwork architecture that captures universal representations by integrating static and statistical descriptors of compounds. We demonstrate the exceptional performance of IonNet in predicting ion mobility, consistently outperforming 16 ablation study combinations and previous deep learning models. Leveraging the universal adaptability of chemical representations, IonNet not only uncovers 87 FICs among ∼4500 stable perfectly stoichiometric compounds but also efficiently pinpoints ∼63,000 prospective FICs from ∼5 million substituted compounds. This study not only presents a full-chain artificial intelligence tool for identifying FICs but also offers compositional principles governing ion mobility, thereby accelerating the development of energy storage and conversion.

## Teaser

A descriptor-guided transfer learning framework was proposed to predict the ion mobility of compounds.

## INTRODUCTION

All-solid-state batteries (ASSBs) are gaining notably attention as next-generation energy storage solutions for consumer electronics and electric vehicles. By replacing the flammable organic liquid electrolyte in traditional batteries with inorganic solid electrolytes (SEs), this design not only improves safety but also allows for the integration of high-energy electrodes, leading to increased energy density (*1*–*3*). A key challenge to realize such batteries is to develop SEs with high ionic conductivity (σ) and excellent electrochemical stability toward the lithium (Li) metal anode and high-voltage cathode (*4*, *5*). Materials that exhibit high σ, typically exceeding 1.0 × 10^−4^ S cm^−1^ at room temperature (∼300 K), also called fast-ion conductors (FICs), not only substantially reduce cell impedance and potentially increase active materials loading in the cathode composite but have also recently been found to reduce the accumulation of mechanical stress in the Li metal anode (*6*–*8*). Thus far, FICs have been reported only in a handful of sulfide-based compounds [for example, Li_10_GeP_2_S_12_ (*9*) and Li_7_P_3_S_11_ (*10*)] and oxides {such as garnets (Li_7_La_3_Zr_2_O_12_) (*11*), NASICON [Na (sodium) superionic conductor]–type oxides (Li_3_Fe_2_P_3_O_12_) (*12*), and perovskites (Li_5_La_3_Ta_2_O_12_) (*13*)}. The slow progress in finding FICs is primarily due to the complexity of experimental procedures and the high cost of ab initio molecular dynamics (AIMD) simulations (*14*, *15*); however, this process could be accelerated if the structural and compositional features that control the facile Li^+^ migration could be fully exploited.

An effective approach is to look for similarities in the crystal structure or atomic arrangement among the few existing FICs and define them as potential principles for identifying unknown FICs. For instance, Ceder and colleagues (*16*) found a corner-sharing (CS) framework as a structural feature common to many oxide FICs. This concept was experimentally validated in LiGa(SeO_3_)_2_ that shows a high bulk σ of 1.1 × 10^−4^ S cm^−1^. The CS framework makes it easier to form reduced-repulsion channels in the structure and minimizes the interaction of Li^+^ with non-Li cations and propagating in a relatively flat energy landscape. The flexibility of CS connections can also promote the relaxation of the framework to accommodate Li^+^ through the bottleneck, thereby facilitating Li^+^ migration. Later, a face-sharing configuration was further identified as an indicator for mining FICs from face-centered cubic oxides (*17*), validated by the overstoichiometric Li-In-Sn-O compound that exhibits a high σ of 3.38 × 10^−4^ S cm^−1^. Structure-based descriptors can sharpen the screening pipeline and lower computational or experimental costs, but they are inevitably limited by their dependence on local structural motifs. It is crucial to acknowledge that compounds lacking these descriptors may be erroneously classified as ordinary conductors. Compositional representations, in contrast, provide a structure-agnostic alternative that expands the search space and allows exploration of candidates where structural information is missing or ambiguous. Rather than replacing structure-driven approaches, composition-based methods offer a complementary perspective for finding ion conductors.

Before experimental investigation, machine learning (ML) techniques, including graph neural networks (GNNs) (*18*, *19*) and generative models (*20*–*22*), have emerged as feasible preprocessing steps for finding high-performance materials. These approaches offer an enticing avenue to establish a potential mapping framework between compounds and σ across the vast chemical space, thereby expediting the discovery of FICs (*23*, *24*). For instance, Zhang *et al.* (*25*) proposed a subgraph isomorphism matching method to meticulously assess the topological similarity between the skeleton framework of distinct compounds. This method prioritizes the local atomic environment over global features such as lattice symmetry. To demonstrate its efficacy, NASICON-type LiTi_2_(PO_4_)_3_ was used as a case study. By the subgraph isomorphism matching method, they identified 13 candidates, such as Li_3_X_2_(PO_4_)_3_ (X = In, Bi, Sb, and Sc), that exhibited σ comparable to that of the LiTi_2_(PO_4_)_3_ prototype (1.47 × 10^−6^ S cm^−1^). From a more nuanced perspective, to examine the relationship between ion hopping events and multiple mobile ions, Cazorla and colleagues (*26*) used *K*-means clustering models to explore the regularity of ion coordination in FICs, leveraging AIMD-based data of 61 materials [46% contain Li, 23% contain halides, 15% contain Na, 8% contain O (oxygen), and 8% contain Ag/Cu (silver/copper)]. Their findings revealed that fast-ion diffusion is strongly positively correlated with sufficient hopping length and long hopping spans but is independent of high hopping frequency and short gap residence times at gaps. These insights offer profound physical insights for the design of FICs. The emergence of ML force fields (MLFFs), such as GNoME (*27*), CHGNet (*28*), and M3GNet (*29*), has revolutionized the simulation of atomic dynamic displacements. These advanced methods have notably accelerated AIMD simulations, enabling them to extend over tens or even hundreds of picoseconds with substantial efficiency.

Despite these promising technological approaches, the number of known FICs remains relatively small compared to the vast unexplored chemical space. Existing advanced graph-based models [e.g., CGCNN (*30*) and MEGNet (*31*)] and crystalline generative models [e.g., MatterGen (*22*) and MatterSim (*32*)] rely on precise crystal structures as inputs. These approaches typically construct graphs from atomic coordinates and local bonding environments, where the structural topology and interatomic distances play a central role in determining model representations. As a result, their applicability becomes limited in many experimental datasets where only compositional information is available and reliable crystal structures are unknown. In such cases, the absence of structural descriptors prevents these models from forming meaningful graph representations, leading to degraded predictive performance and making structure-based frameworks difficult to apply. Conversely, numerous ion conductors with available high-coverage chemical compositions remain underused, with far less attention given to chemical compositions than to structure-dominated frameworks, highlighting a substantial gap in leveraging existing information for discovery and optimization. Another hotly debated open challenge is that the prediction of structures and properties of inorganic compounds with impurities or defects has not yet been solved, which makes the extension of existing known FICs into the doped chemical space a slow and tentative study based on human intuition. Moreover, the scarcity and heterogeneity of experimental datasets, contrasted with the relative abundance of computational data, make knowledge-reused techniques [e.g., transfer learning (TL)] a particularly compelling strategy to decode the chemical space of FICs (*33*, *34*).

Here, we proposed an attentional TL framework, named IonNet, as a chemical compositional indicator for arbitrary inorganic compounds. IonNet effectively and fully uses theoretical prediction data and experimental data in terms of data scale and σ evaluation level. By using IonNet, from more than 4000 perfect inorganic compounds, we identified 87 unknown compounds that were predicted to be FICs. Unrestricted by the crystalline structure, IonNet offers predictive capabilities across any doping chemical compositions. Among nearly 5 million candidates, IonNet identifies 62,935 potential FICs and elucidates the dynamic effects of varying doping proportions on σ. This approach addresses limitations of relying solely on topological structures, enabling its application to doping compounds and bridging the gap in the precise design of FICs on the basis of chemical composition. Furthermore, it holds promise for integration with structural factor–based screening processes, thereby enhancing the precision of FIC evaluations.

## RESULTS

### Description of the IonNet framework

**Fig. 1.**
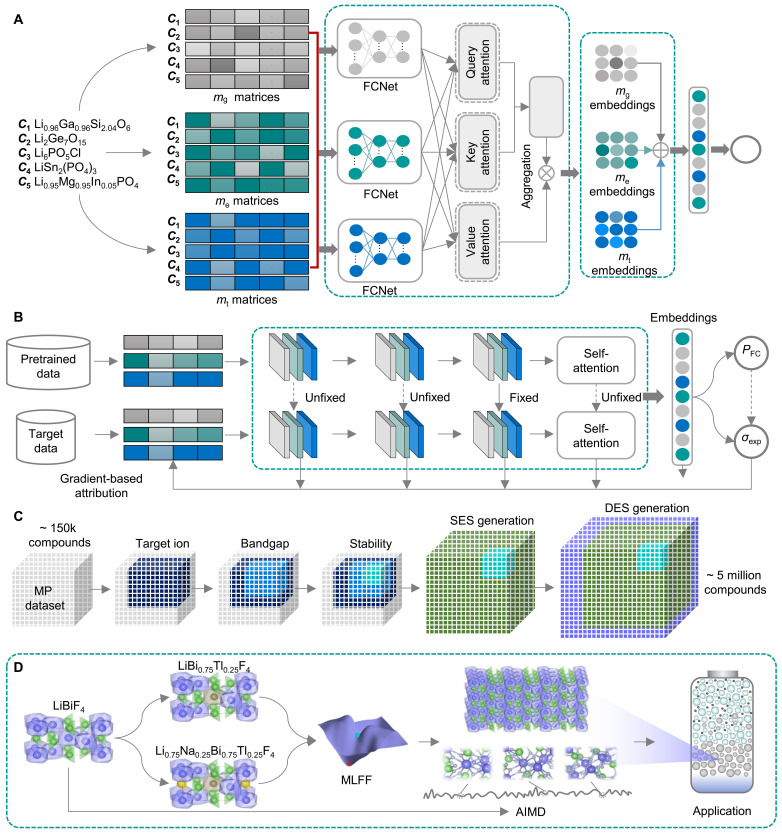
Diagram for illustrating the IonNet framework and application. (**A**) Descriptor-specialized architecture, where three mainstream compositional descriptors were fed into three independent self-attention subnetworks: *m*_g_, *m*_e_, and *m*_t_. The FCNet is appended to project the input descriptors into the *Q*, *K*, and *V* spaces to capture long-range dependencies. (**B**) TL framework from the computational property (*P*_FC_) to the experimental σ. (**C**) Identification of FICs from the MP database and substituted compounds using SES and DES. (**D**) Validation of FICs via AIMD simulations. The crystal structure generation of substituted compounds can be implemented using MLFFs.

Here, we developed a descriptor-guided TL framework (IonNet) for the accurate prediction of FICs. Drawing inspiration from atom-centered potential energy surface models (*35*), we assembled three mainstream compositional descriptors into three independent self-attention subnetworks to extract latent features and effectively represent ion conductors: meredig (*m*_g_) (*36*), magpie (*m*_e_) (*37*), and MEGNet (*m*_t_) (Fig. 1A) (*31*). The first two types of descriptors are derived from chemical composition and capture the static and statistical properties of elements, thereby fully exploiting information encoded in elemental identities and stoichiometric ratios. In contrast, the *m*_t_ originates from graph embeddings trained on a large crystal structure database, yielding descriptors that are more nonlinear and capable of incorporating structure-informed elemental priors (see the details in Supplementary Text and fig. S1).

To capture long-range dependencies, IonNet uses the fully connected network (FCNet) to project the input descriptors into the query (*Q*), key (*K*), and value (*V*) spaces. Each self-attention subnetwork feeds the generated *Q*, *K*, and *V* into the attention module to compute the weighted embeddings, preserving the global dependency information of the chemical characteristics. The σ is regarded as the sum of the contributions of each descriptor. In contrast to conventional ensemble models, which combine outputs of multiple learners in a post hoc manner, IonNet jointly trains modality-specific subnetworks and integrates them through self-attention. This design ensures that each feature channel contributes distinct and complementary information while allowing its relative importance to be adaptively adjusted for different samples. Compared to simple feature concatenation, the multichannel architecture preserves modality-specific representations and avoids information dilution, thereby enabling synergistic interactions across features that are crucial for accurate property prediction (Supplementary Text and fig. S2).

**Fig. 2.**
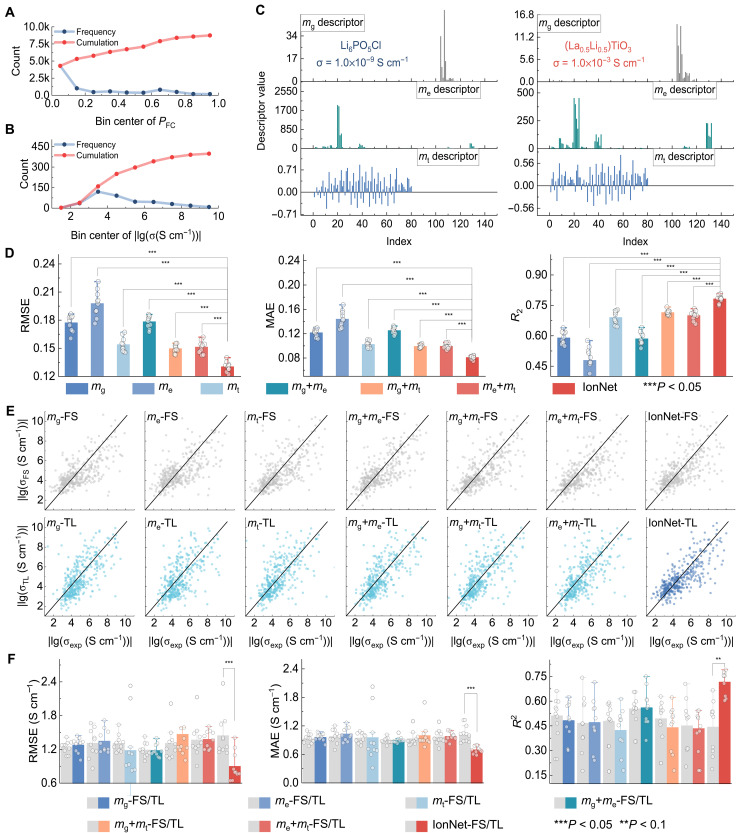
Dataset, descriptor visualization, and performance evaluation of IonNet. (**A**) Statistic of 8750 uniquely calculated *P*_FC_. (**B**) Statistic of 398 experimentally measured σ at log_10_ scale. The frequency within the interval is shown in blue, while the cumulative count is shown in red. (**C**) Visualization of 120-dimensional *m*_g_ (in gray), 132-dimensional *m*_e_ (in green), and 80-dimensional *m*_t_ (in blue) for representing experimental data Li_6_PO_5_Cl (ordinary ion conductor, σ = 1.0 × 10^−9^ S cm^−1^) and (La_0.5_Li_0.5_)TiO_3_ (FIC, σ = 1.0 × 10^−3^ S cm^−1^), giving the numerical difference between fast-ordinary ion conductors and in three descriptors. (**D**) Performance evaluation and ablation studies based on the computational dataset (*P*_FC_ prediction) across various descriptor combinations. Pairwise *t* tests between the IonNet model and other baseline models were conducted, with the corresponding *P* values provided (*n* = 10). (**E**) Scatterplots of the experimental measurements and model predictions. The models were trained using various descriptor combinations in FS (row above) training and TL (row below) modes. All the data in the validation sets of 10-fold cross-validation were selected for display. (**F**) Performance evaluation and ablation studies based on the experimental dataset (σ prediction) in FS and TL modes across various descriptor combinations using 10-fold cross-validation. Pairwise *t* tests between the IonNet-TL model and the IonNet-FS model were conducted, with the corresponding *P* values provided. The metrics RMSE, MAE, and *R*^2^ were selected to evaluate the models, with their mean and standard deviation (SD) on validation data calculated on the basis of 10-fold cross-validation (*n* = 10).

IonNet is pretrained on a unique set of 8750 compounds of Li^+^-containing conductors from the open database (Fig. 2A) (*38*), which integrates structural features (space group and lattice symmetry), chemical compositions (chemical fraction), and Li^+^ site attributions (mobility channel and effective ratio), to optimize the latent representation of compounds (Fig. 1B and Supplementary Text). The pretrained model is then fine-tuned on a downstream task using experimental data (Fig. 2B and table S1), further enhancing its performance in predicting the σ. After fine-tuning the model, IonNet was applied on a large scale to ∼150,000 perfectly stoichiometric compounds in the Material Project (MP) database (*39*), enabling the screening of FICs on the basis of additional performance indicators of SSEs, such as bandgap and thermodynamic stability (Fig. 1C). Furthermore, partial replacement methods, single-element substitution (SES), and double-element substitution (DES) are used to expand the substituted compound space to the ∼5 million–level scale, from which IonNet was used to identify potential FICs. For the candidate FICs, AIMD simulations were conducted to evaluate the σ for validations, and experiments can be performed on the most promising candidate (Fig. 1D). It is worth noting that for substituted compounds, an additional step of crystal structure generation is required using MLFFs.

### Performance evaluation and ablation analysis of IonNet

Two datasets were used to evaluate the performance of the IonNet: (i) computational probabilities (0.0 to 1.0) of being FICs (*P*_FC_) from the IonML platform (*38*), with 8750 entries (Fig. 2A); (ii) experimentally measured σ (10^−1^ to 10^−10^ S cm^−1^) from the previous published literature (*40*), with 398 entries (Fig. 2B). Both datasets are dominated by oxygen-containing compounds, which constitute 69.4 and 79.4%, respectively. Phosphorus-containing compounds also represent a substantial fraction in both datasets, suggesting partial overlap in compositional categories. Moreover, the broader compositional coverage of computational data may facilitate more effective model pretraining and provide a richer representation basis for subsequent fine-tuning on the experimental system (fig. S3 and table S2). The smoothing of the cumulative curves ensures that the data distribution is continuous and balanced, providing the rationality of using the regression model for predicting the *P*_FC_ and the σ. Figure 2C provides the visualization of three descriptors (120-dimensional *m*_g_, 132-dimensional *m*_e_, and 80-dimensional *m*_t_) for representing experimental data Li_6_PO_5_Cl (σ = 1.0 × 10^−9^ S cm^−1^) and (La_0.5_Li_0.5_)TiO_3_ (σ = 1.0 × 10^−3^ S cm^−1^), giving the numerical difference between fast-ordinary ion conductors and in three descriptors, which may be the key chemical knowledge to be learned by the IonNet (Materials and Methods).

Training IonNet in from-scratch (FS) mode on large-scale computational data is crucial for enabling its subsequent use in TL mode on experimental data. This strategy also highlights the ability of IonNet to capture potential mappings between chemical compositions and ion migration–related properties (*33*, *34*, *41*). To validate this, we first assessed the model performance on computational data (Table 1). For this regression task predicting the *P*_FC_, IonNet achieves a root mean squared error (RMSE), a mean absolute error (MAE), and a coefficient of determination (*R*^2^) of 0.130 ± 0.005, 0.081 ± 0.003, and 0.782 ± 0.018, respectively (Fig. 2D and fig. S4). This shows that on the basis of the computational data, the IonNet as a whole is able to predict the probability that a compound is an FIC with a smaller error. We further conducted ablation experiments on computational data as the baseline models. As can be seen from Fig. 2D, IonNet uses an integration of three descriptors and shows obvious advantages in model performance. Pairwise *t* tests between IonNet and baseline models yield *P* values below 0.05, confirming the statistically significant superiority of IonNet in predicting the *P*_FC_. This illustrates the complementarity of static element features (*m*_g_), statistical element features (*m*_e_), and nonlinear compositional features based on graph structures (*m*_t_). It also shows the advantage of this parallel architecture in improving model robustness. We also note that the *m*_t_ descriptor has an advantage in computational data (RMSE = 0.154 ± 0.008, MAE = 0.103 ± 0.005, and *R*^2^ = 0.691 ± 0.031), which is likely due to its more general chemical composition information obtained from the MEGNet (*31*) and its better versatility on the relatively large-scale computational data of this work.

**Table 1.** Performance of various descriptors on the computational and experimental data based on FS training and TL. The mean and standard deviation (SD) on validation data were calculated on the basis of 10-fold cross-validation. The units for RMSE and MAE of experimental data are S cm^−1^ at log_10_ scale.

Model	Computational data			Experimental data (FS)			Experimental data (TL)		
	RMSE	MAE	*R* ^2^	RMSE	MAE	*R* ^2^	RMSE	MAE	*R* ^2^
*m*_g_	0.177 ± 0.009	0.122 ± 0.006	0.590 ± 0.031	1.247 ± 0.106	0.936 ± 0.100	0.513 ± 0.117	1.283 ± 0.133	0.965 ± 0.077	0.484 ± 0.115
*m*_e_	0.198 ± 0.014	0.144 ± 0.013	0.479 ± 0.071	1.314 ± 0.256	0.983 ± 0.150	0.468 ± 0.169	1.352 ± 0.203	1.035 ± 0.140	0.473 ± 0.146
*m*_t_	0.154 ± 0.008	0.103 ± 0.005	0.691 ± 0.031	1.314 ± 0.152	0.950 ± 0.103	0.480 ± 0.132	1.186 ± 0.598	0.960 ± 0.538	0.425 ± 0.136
*m*_g_ + *m*_e_	0.178 ± 0.006	0.125 ± 0.005	0.586 ± 0.032	1.194 ± 0.128	0.896 ± 0.062	0.555 ± 0.087	1.185 ± 0.111	0.906 ± 0.063	0.562 ± 0.116
*m*_g_ + *m*_t_	0.150 ± 0.004	0.099 ± 0.003	0.715 ± 0.013	1.313 ± 0.287	0.937 ± 0.153	0.495 ± 0.118	1.472 ± 0.593	1.001 ± 0.262	0.441 ± 0.142
*m*_e_ + *m*_t_	0.151 ± 0.007	0.100 ± 0.004	0.700 ± 0.023	1.372 ± 0.321	0.972 ± 0.155	0.452 ± 0.160	1.384 ± 0.157	0.985 ± 0.092	0.435 ± 0.117
*m*_g_ + *m*_e_ + *m*_t_ (IonNet)	0.130 ± 0.005	0.081 ± 0.003	0.782 ± 0.018	1.449 ± 0.381	1.014 ± 0.157	0.445 ± 0.162	0.904 ± 0.257	0.702 ± 0.075	**0.717 ± 0.072**

To examine whether the size of the source domain is sufficient for pretraining, we performed additional experiments using subsets of the source dataset with 1000, 2000, 4000, and 6000 samples. As shown in fig. S5, the prediction error consistently decreases as the number of training samples increases, indicating that the model benefits from larger datasets and that the pretraining process remains stable across different data scales. Notably, the model trained on the full dataset of 8750 samples achieves the best performance, suggesting that the current dataset size already provides substantial learning capacity for the proposed framework. At the same time, the observed trend implies that incorporating larger datasets in the future could further improve the generalization ability of the pretrained model.

We then compared the performance of the IonNet framework in both the FS and TL modes, as shown in the far right of Fig. 2E. For this regression task, IonNet trained in TL mode (IonNet-TL model) achieves the RMSE, MAE, and *R*^2^ of 0.904 ± 0.257, 0.702 ± 0.075, and 0.717 ± 0.072, respectively (Table 1 and fig. S6), which is much better than the performance of IonNet in FS training (IonNet-FS model, serving as a baseline model; RMSE = 1.449 ± 0.381, MAE = 1.014 ± 0.157, *R*^2^ = 0.445 ± 0.162). Ablation experiments and pairwise *t* tests were also conducted on experimental data in both FS and TL modes (Fig. 2F). When FS is used, integrating three descriptors is not the best choice, but combining *m*_g_ and *m*_e_ (*m*_g_ + *m*_e_-FS model; RMSE = 1.194 ± 0.128, MAE = 0.896 ± 0.062, *R*^2^ = 0.555 ± 0.087), to some extent, reflects the complementarity of the two elemental descriptors in representing chemical compositions. We speculate that the poor performance of *m*_t_ in FS-based models is because *m*_t_ comes from MEGNet models trained on large-scale data, which may lead to overfitting when used alone for training on relatively small-scale data. In fig. S7, we found that the performance differences between different network layer freezing strategies are not substantial, indicating that the model has a certain degree of stability and can reflect the correlation between the source and target tasks. The optimal strategy was determined on the basis of the performance metrics.

Furthermore, the performance of IonNet was compared with recent predictive models. The MAEs of the AIMD-based model (O-sequence), hybrid multisource fingerprint-based model (M-feature), variational autoencoder model (V-AIMD), and transformer-based model (T-AIMD) are 1.093, 1.031, 1.097, and 0.927, at log_10_ scale, respectively (*42*), demonstrating a higher predictive error than 0.702 of IonNet. These results demonstrate the improved predictive accuracy for conductivity-related tasks. A detailed quantitative comparison is provided in Table 1 and table S3. Beyond the direct comparison in σ prediction, it is important to place this work within the broader landscape of ML for SE discovery. For instance, recent studies have demonstrated that ML can be applied to a wide range of properties, including bandgap, ion migration barriers, mechanical modulus, and thermal expansion (*43*). These studies highlight the growing trend toward multiproperty prediction and holistic materials design, rather than focusing on a single target property.

### Chemical interpretation of IonNet

**Fig. 3.**
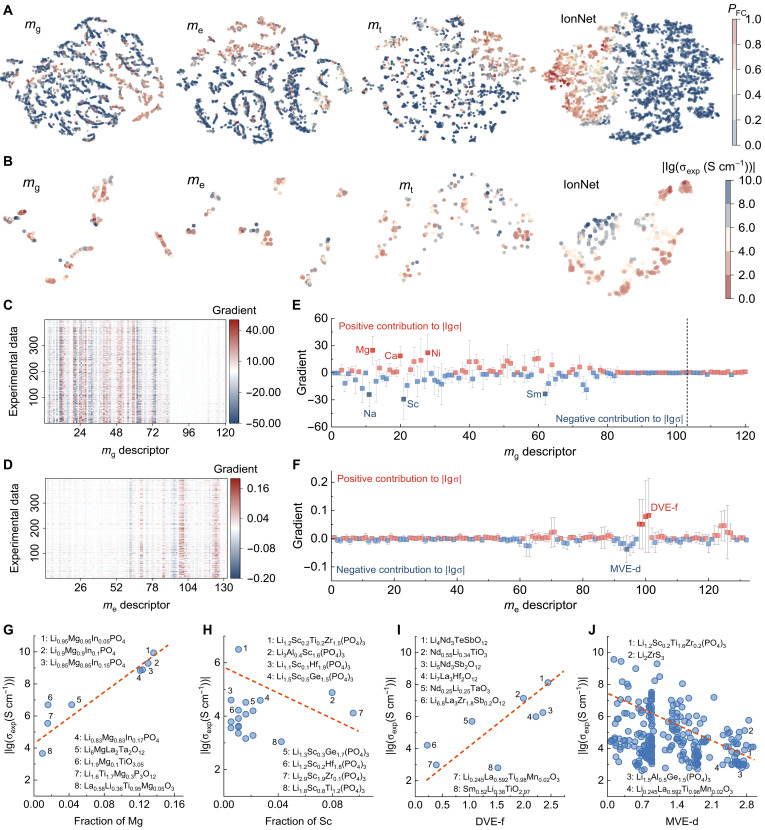
Chemical interpretation of IonNet. (**A**) t-SNE visualization of the computational data using individual descriptors (*m*_g_, *m*_e_, or *m*_t_) as well as the latent features from the concatenated output layer of IonNet, presented with color gradients ranging from blue (low *P*_FC_) to red (high *P*_FC_). (**B**) t-SNE visualization of the experimental data, presented with color gradients ranging from red (low |lgσ|) to blue (high |lgσ|). (**C** and **D**) Gradient visualization of IonNet using the 120-dimensional *m*_g_ and 132-dimensional *m*_e_ descriptors by the GBA method. (**E** and **F**) AG of each feature of *m*_g_ and *m*_e_, highlighting the features that IonNet prioritizes during the decision-making process. Given that descriptors are introduced into different subnetworks, only the absolute values of gradients are compared under the same descriptor. The error bars are based on the cross-validation models (*n* = 10). (**G** to **J**) Scatterplots of key features (fraction of Mg, fraction of Sc, DVE-f, and MVE-d) showing the correlation with the σ. The representative compounds from the experimental dataset are presented, and the red dashed lines indicate the trends (positive or negative) between features with |lgσ|.

We then used t-distributed stochastic neighbor embedding (t-SNE) to visualize the representations derived from different models, aiming to investigate the chemical interpretation of IonNet (*44*). Figure 3A presents the scatterplots generated from the computational data using individual descriptors (*m*_g_, *m*_e_, or *m*_t_) as well as the latent features from the concatenated output layer of IonNet. In contrast, Fig. 3B shows scatterplots based on the experimental data. These plots reveal that the latent features identified by IonNet are well clustered according to their strong ion migration properties (high *P*_FC_ or high σ). Moreover, the descriptor distributions of computational and experimental data (fig. S8) exhibit a high degree of overlap overall, which means that the global distribution differences between the two domains in the high-level feature space are small. These observations highlight IonNet’s ability to effectively extract discriminative features from descriptors for downstream tasks and to improve predictive performance for finding FICs. To further investigate the potential domain gap between the computational dataset and the experimental dataset, we analyzed the learned embeddings using the maximum mean discrepancy and Wasserstein distance and observed a notable reduction compared to the raw feature space (table S4), indicating improved feature alignment. These results demonstrate that the proposed framework is relatively robust to distribution shifts between the source and target domains. To identify the key chemical factors influencing the σ, a gradient-based attribution (GBA) method was used to evaluate feature importance (*45*). For each subnetwork, the gradient (Δσ/Δ**X***_i_*) of the network output with respect to a specific input feature vector **X***_i_* was computed. The element-wise product of the gradient and the feature value was then calculated as an indicator of the feature importance. Compared with model-agnostic interpretation approaches such as Shapley additive explanations and permutation importance, GBA offers a more computationally efficient and direct way to probe deep neural networks (NNs) by leveraging model gradients. This is particularly advantageous for the present high-dimensional feature space with several hundred descriptors, where model-agnostic methods can become prohibitively expensive. To further ensure robustness, we evaluated the consistency of GBA rankings across cross-validation folds and analyzed the average gradient (AG). Figure 3 (C and D) illustrates the GBA visualization of all 398 experimental samples using the 120-dimensional *m*_g_, the 132-dimensional *m*_e_ descriptors, and the 80-dimensional *m*_t_ descriptors (fig. S9). IonNet accurately captures both static elemental properties and statistical elemental properties, simultaneously focusing on the σ. Figure 3 (E and F) displays the AG of each feature, highlighting the features that IonNet prioritizes during the decision-making process (table S5). Notably, some key features are marked, such as the fraction of magnesium (Mg; AG = 24.79) or scandium (Sc; AG = −29.25) in the chemical composition, as well as the deviation of valence electrons in the unfilled f orbital (DVE-f; AG = 0.083) and the mean of valence electrons in the unfilled d orbital (MVE-d; AG = −0.037) across the constituent elements, etc. As shown in Fig. 3E, the model exhibits notable differences in the AG distribution for the first 83 elements [H (hydrogen), He (helium), Li, …, Tl (thallium), Pb (lead), and Bi (bismuth)], which predominantly constitute the compounds. This observation highlights two key points: First, IonNet effectively captures the fractional information of individual elements; second, it underscores the fact that the diversity of elemental composition influences σ, extending beyond the contribution of a single element’s fraction.

We further conducted a sensitivity analysis of the key features and identified all compounds in the experimental dataset containing Mg or Sc, such as Li_0.95_Mg_0.95_In_0.05_PO_4_ and Li_1.2_Sc_0.2_Ti_0.2_Zr_1.5_(PO_4_)_3_. The fraction of Mg (Fig. 3G) or Sc (Fig. 3H) was then correlated with the predicted values (|lgσ|). A distinct positive or negative correlation trend emerged, particularly for the fraction of Mg, which exhibits a strong positive correlation with |lgσ| in the existing data. This observation suggests that introducing a small amount of Mg doping (∼1%) may enhance Li^+^ dynamics, whereas excessive Mg concentrations (∼10%) could hinder the migration of Li^+^. At low doping levels, Mg^2+^ likely acts as an aliovalent modifier that increases mobile Li^+^ carrier concentration and slightly lowers migration barriers via vacancy creation and mild lattice disorder, thereby enhancing σ. At high Mg^2+^ concentrations, however, site blocking, stronger Mg^2+^-anion and Mg^2+^-Li^+^ interactions, structural distortion, or clustering can disrupt Li^+^ percolation pathways and increase migration barriers, thereby decreasing σ. Conversely, the fraction of Sc within the composition exhibits a weaker negative correlation with |lgσ|, implying that higher levels of Sc doping might improve Li^+^ migration. This effect may be attributed to the close ionic radii of Sc^3+^ (0.745 Å, six-coordination) and Li^+^ (0.76 Å, six-coordination). Sc doping likely induces oxygen vacancies or other point defects, which in turn provide additional migration paths or sites for Li^+^, enhance diffusion channels, and facilitate more efficient migration of Li^+^.

These observations suggest several experimental relevant design principles for FIC design: (i) Moderate aliovalent doping can enhance σ by increasing the concentration of mobile Li^+^, (ii) excessive dopant concentrations should be avoided to prevent site blocking and disruption of Li^+^ percolation networks, and (iii) dopants with ionic radii close to that of Li^+^ may be particularly effective in introducing beneficial defects while maintaining structural stability.

We also analyzed the influence of DVE-f (Fig. 3I) and MVE-d orbitals (Fig. 3J). The large DVE-f indicates that the elements involved in the formation of the compound have different f electronic properties (Supplementary Text). It can be seen from Li_4_Nd_3_TeSbO_12_ (DVE-f = 2.45) and Nd_0.55_Li_0.34_TiO_3_ (DVE-f = 2.00) in Fig. 3I that large deviations may lead to enhanced localization of f electrons, which in turn increases the local lattice rigidity. In severe cases, it may cause serious distortion of the crystal lattice and inhibit the migration ability of Li^+^. This phenomenon may be particularly notable in some rare-earth element (La, Nd, Pr, etc.)–doped materials, given that the localization of f electrons affects the Li^+^ permeability of the overall lattice. In Fig. 3J, a larger MVE-d, corresponding to a greater number of d-orbital electrons, results in a more symmetrical electron cloud distribution around the metal center. This contributes to a more uniform charge distribution within the crystal, thereby reducing the electric field gradient encountered by Li^+^ in the lattice. Consequently, the migration barrier is lowered, facilitating the migration of Li^+^. In addition, a higher number of unfilled d-orbital electrons could stabilize oxygen vacancies or other point defects by modulating lattice chemical bonds, such as enhancing the flexibility of metal-oxygen bond strengths. The presence of more d electrons improves the adaptability of bond lengths and the dynamic response of the lattice. During migration, the lattice can adjust more efficiently and at a lower energy cost, further reducing the diffusion barrier for Li^+^.

These observations also provide useful guidance for the experimental design of FICs. In particular, the results suggest that the electronic structure of the framework cations plays an important role in regulating Li^+^ transport. Materials containing elements with highly localized f electrons (e.g., certain rare-earth elements) may introduce increased lattice rigidity and local structural distortions resulting from strong f-electron localization. Therefore, when designing SEs, excessive incorporation of f electron–rich elements should be carefully controlled, and compositions that minimize strong f-electron localization may be preferable for maintaining a flexible diffusion lattice. In contrast, a higher average number of unfilled d orbitals can lead to a more symmetric electronic distribution around the metal centers. This can reduce local electric-field gradients experienced by migrating Li^+^ ions and lower migration barriers. Moreover, transition metals with partially filled d orbitals may help stabilize oxygen vacancies or other point defects through flexible metal-oxygen bonding, thereby providing additional Li^+^ migration pathways. Consequently, incorporating suitable transition-metal elements [e.g., Ti (titanium) and Zr (zirconium)] with accessible d orbitals may represent an effective strategy to enhance σ in future experimental designs of FICs.

### Identifying FICs for SEs via IonNet

Global Li-ion battery demand is projected to reach 2000 GW·hour by 2030, with ASSBs emerging as the core technology for next-generation energy storage (*46*, *47*). Traditional liquid electrolytes constrained by safety and performance limitations are unable to meet this demand. Battery accidents such as electrolyte thermal runaway result in economic losses exceeding $1 billion annually, underscoring the urgent need for safe and efficient Li^+^ conductors (*48*, *49*).

**Fig. 4.**
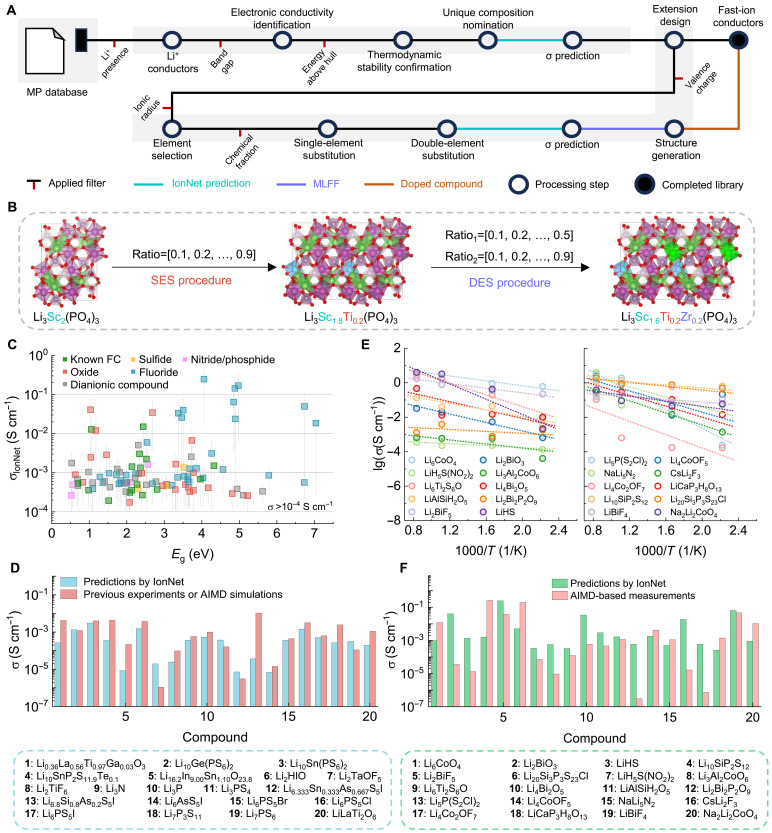
Identifying FICs for SEs via IonNet. (**A**) Screening pipeline for FICs. Compounds were retrieved from the MP database, focusing on those containing Li^+^. To meet SE requirements, compounds with a bandgap >0.5 eV and an *E*_hull_ <0.1 eV per atom were selected. Unique chemical formulas were further screened, retaining only the most thermodynamically stable structure. Next, SES and DES were used to partially substitute the original chemical compositions, considering ionic radius, valence, and element ratios. Subsequently, high-throughput predictions were then performed using the IonNet model. (**B**) Schematic diagram of SES and DES processes, taking Li_3_Sc_2_(PO_4_)_3_ as an example. (**C**) 102 FICs predicted by IonNet, categorized on the basis of their anions. The error bars are based on the cross-validation models (*n* = 10). (**D**) Comparison between the experimentally measured or AIMD-calculated σ of known compounds with the IonNet-predicted σ at 300 K. (**E**) Arrhenius plot of the bulk σ of 20 Li^+^ conductors by AIMD simulations under different temperatures (1200, 900, 600, and 450 K). The *E*_a_ values (Table 2) of Li^+^ migration according to the Arrhenius relationship are also provided (*68*). (**F**) Comparison between the AIMD-calculated σ of compounds with the IonNet-predicted σ at 300 K.

To address this challenge, we tested IonNet to identify potential FICs among a diverse range of inorganic compounds in the MP database (154, 718 entries; Fig. 4A) (*39*). The screening process incorporated key criteria such as thermodynamic stability [energy above hull (*E*_hull_) <0.1 eV per atom] and electronic conductivity (bandgap >0.5 eV), aligned with the operation requirements of SEs (*50*, *51*). High-throughput predictions were performed on the remaining 4583 unique chemical composition–encoded compounds, identifying 102 promising candidates (fig. S10 and table S6). Previous studies have shown that evaluating the σ of a single material via AIMD typically requires on the order of 23,000 to 52,000 CPU hours (*38*, *52*). In contrast, our model can generate predictions within only a few seconds, from feature computation to inference. This marked improvement in efficiency (approximately eight orders of magnitude speedup) enables large-scale screening of FICs and provides a valuable prescreening tool before experimental validation and application.

Building on this, we leveraged the ability of IonNet to perform predictions across any chemical compositions, expanding our search to the broader chemical space of doping compounds. Specifically, SES and DES were used to partially substitute the original chemical compositions (Fig. 4B), with considerations for factors such as ionic radius, valence, and element ratios. For the ionic radius and valence, substitution of elements in the same group in the periodic table was mainly considered (e.g., Ba^2+^: Be^2+^, Mg^2+^, Ca^2+^, and Sr^2+^; Cr^3+^: Mo^3+^ and W^3+^). Some transition metals were also considered given that they exhibit multiple valence electrons (e.g., Co^2+^: Fe^2+^, Mn^2+^, Ni^2+^, Ru^2+^, Rh^2+^, Pd^2+^, and Pt^2+^). The complete element substitution rules are shown in table S7. For the SES procedure, element replacement ratios of 0.05, 0.1, 0.15, 0.2, 0.25, 0.3, 0.35, 0.4, 0.45, and 0.5 were adopted. In contrast, the DES procedure was carried out in two stages: first, selecting the overall replacement ratio (0.1, 0.2, 0.3, 0.4, and 0.5) and, second, assigning the ratio between the two replacing elements (0.1 to 0.9 in increments of 0.1). Substitution cases can be found in fig. S11.

This approach resulted in 624,460 SES candidates and 4,316,850 DES candidates (fig. S12), from which we identified additional 11,403 and 51,532 promising candidates, respectively. The data have been released publicly (see Data, code, and materials availability). It should be noted that the crystal structures of these doped compounds can be preliminarily optimized by MLFFs because of the strict elemental candidate substitutions. Typical MLFFs can achieve speedups ranging from 10^3^ to 10^6^ compared to density functional theory calculations and AIMD simulations, depending on the system size and the specific ML model used (*53*, *54*). This marked improvement in efficiency allows for longer timescales and larger system sizes to be accessed, making it feasible to investigate rare events and defect dynamics that would otherwise be prohibitively expensive with AIMD. In this work, the MLFF script is included in the code, thus saving the computing costs of density functional theory calculations. Examples for structural optimization using MLFFs are provided in fig. S13. Through element substitution, we generated a wide range of previously unknown compositions and used our model to predict their σ, aiming to identify promising FICs. At this stage, we did not impose constraints related to the cost, toxicity, or environmental impact of the substituted elements, although such considerations are essential for practical implementation. By deliberately excluding these factors during the computational stage, we are able to maximize the exploration of the chemical space and uncover potentially high-performing candidates.

Figure 4C presents the 102 FICs predicted by IonNet from the MP database, categorized on the basis of their anions (table S6). The green markers represent known compounds, including those from our training dataset (e.g., Li_3_PS_4_ and Li_6_PS_5_I), as well as others identified in experimental studies but not included in the training data [e.g., Li_7_PS_6_ and Li_10_Ge(PS_6_)_2_]. We then compared the experimentally measured or AIMD-calculated σ of these compounds with the σ values predicted by the IonNet model (Fig. 4D and table S8), demonstrating the model’s high-precision predictive capability (*55*–*61*). Certain compounds, such as Li_3_BiO_4_, Li_3_BiO_3_, and LiBiO_2_, are identified as FICs because of their low ionic activation energy (*E*_a_) barriers (0.37, 0.33, and 0.10 eV, respectively), even though their σ values are not particularly high. This analysis serves two purposes: examining the IonNet’s ability to accurately predict known compounds and assessing its potential to extrapolate and identify unseen conductors.

### Validation of promising FICs

For the unknown 87 compounds (table S6), we further investigated their σ using AIMD simulations. In this step, we prioritized oxides because of their high chemical stability, wide electrochemical stability window, abundance on earth, ease of accessibility, and relatively low synthesis and production costs. Ten oxides that do not contain rare or uncommon elements [e.g., Tl and Hg (mercury)] were selected [Li_6_CoO_4_, Li_2_BiO_3_, LiH_5_S(NO_2_)_2_, Li_3_Al_2_CoO_6_, Li_6_Ti_2_S_6_O, Li_4_Bi_2_O_5_, LiAlSiH_2_O_5_, LiCaP_3_H_8_O_13_, Na_2_Li_2_CoO_4_, and Li_2_Bi_2_P_2_O_9_], along with two sulfides (LiHS and Li_10_SiP_2_S_12_), three halides (CsLi_2_F_3_, LiBiF_4_, and Li_2_BiF_5_), a nitride (NaLi_5_N_2_), and four mixed-anion compounds [Li_4_Co_2_OF_7_, Li_20_Si_3_P_3_S_23_Cl, Li_4_CoOF_5_, and Li_5_P(S_2_Cl)_2_], to ensure balanced representation (Table 2). AIMD simulations were conducted at 1200, 900, 600, and 450 K for 30 ps for each compound, enabling the acceleration of atomic movements and the observation of diffusion behavior within a reasonable simulation time frame. The diffusion coefficients and σ, along with uncertainty quantification, are provided in table S9.

**Table 2.** Summary of properties of screened FICs. The *E*_hull_ and bandgap (*E*_g_) were extracted from the MP database, σ_IonNet_ and σ_IonNet_ + standard deviation (σ_sd_) were predicted by the IonNet model, and σ_AIMD_ was evaluated by the AIMD simulations. The *E*_a_ values were calculated according to the Arrhenius relationship.

Compound	*E*_hull_ (eV/atom)	*E*_g_ (eV)	σ_IonNet_ (S cm^−1^)	σ_sd_ (S cm^−1^)	σ_AIMD_ (S cm^−1^)	*E*_a_ (eV)
Li_6_CoO_4_	0.00	2.40	9.73 × 10^−4^	1.12 × 10^−4^	1.20 × 10^−2^	0.157
Li_2_BiO_3_	0.05	1.04	4.08 × 10^−2^	5.48 × 10^−3^	3.55 × 10^−5^	0.416
LiHS	0.00	3.53	1.35 × 10^−3^	6.53 × 10^−4^	1.32 × 10^−5^	0.523
Li_10_SiP_2_S_12_	0.01	2.56	1.60 × 10^−3^	8.88 × 10^−4^	2.62 × 10^−1^	0.117
Li_2_BiF_5_	0.06	4.06	2.46 × 10^−1^	1.72 × 10^−2^	3.77 × 10^−2^	0.185
Li_20_Si_3_P_3_S_23_Cl	0.03	2.31	5.06 × 10^−3^	3.10 × 10^−3^	1.97 × 10^−1^	0.129
LiH_5_S(NO_2_)_2_	0.09	5.62	3.35 × 10^−4^	1.65 × 10^−4^	7.13 × 10^−5^	0.151
Li_3_Al_2_CoO_6_	0.03	0.83	5.69 × 10^−4^	1.48 × 10^−4^	9.45 × 10^−6^	0.220
Li_6_Ti_2_S_6_O	0.05	1.97	3.29 × 10^−4^	1.18 × 10^−4^	1.22 × 10^−4^	0.552
Li_4_Bi_2_O_5_	0.03	2.68	3.44 × 10^−2^	4.00 × 10^−3^	6.03 × 10^−4^	0.274
LiAlSiH_2_O_5_	0.01	4.81	2.92 × 10^−3^	1.18 × 10^−4^	4.67 × 10^−4^	0.252
Li_2_Bi_2_P_2_O_9_	0.09	3.31	1.65 × 10^−3^	1.13 × 10^−4^	1.14 × 10^−3^	0.077
Li_5_P(S_2_Cl)_2_	0.02	3.23	5.91 × 10^−4^	2.52 × 10^−4^	3.04 × 10^−7^	0.648
Li_4_CoOF_5_	0.08	0.53	1.77 × 10^−3^	2.95 × 10^−4^	4.16 × 10^−3^	0.254
NaLi_5_N_2_	0.08	0.56	4.95 × 10^−4^	1.30 × 10^−4^	1.11 × 10^−3^	0.278
CsLi_2_F_3_	0.06	7.04	1.85 × 10^−2^	2.71 × 10^−4^	1.65 × 10^−5^	0.413
Li_4_Co_2_OF_7_	0.04	1.41	6.05 × 10^−4^	1.29 × 10^−4^	7.39 × 10^−7^	0.400
LiCaP_3_H_8_O_13_	0.04	5.36	2.58 × 10^−4^	1.07 × 10^−4^	1.40 × 10^−3^	0.263
LiBiF_4_	0.00	4.85	6.39 × 10^−2^	4.09 × 10^−3^	4.65 × 10^−2^	0.108
Na_2_Li_2_CoO_4_	0.06	0.63	8.88 × 10^−4^	3.33 × 10^−4^	1.04 × 10^−2^	0.163

In Fig. 4E, the σ values of these 20 compounds at high temperatures, as determined by AIMD simulations, are presented alongside their *E*_a_ derived from the Arrhenius relationship. The σ at 300 K was extrapolated by fitting high-temperature data. Among the 20 compounds analyzed, 13 were confirmed to have σ > 1.0 × 10^−4^ S cm^−1^, and 15 were proved to exhibit *E*_a_ less than the critical threshold of 0.4 eV (Table 2). We also compared the IonNet-predicted σ with the AIMD-based σ (Fig. 4F) to evaluate the prediction accuracy and identify deviations. Only three compounds [Li_2_BiO_3_, LiHS, and Li_5_P(S_2_Cl)_2_] exhibit relatively poor performance in both σ and *E*_a_. While relatively larger errors were observed in the predictions for compounds such as Li_5_P(S_2_Cl)_2_, Li_2_BiO_3_, CsLi_2_F_3_, and Li_4_Co_2_OF_7_, these deviations remain within acceptable limits (see the analysis in Supplementary Text and fig. S14). Furthermore, no systematic bias was found between the IonNet-predicted σ, the experiment-measured σ (Fig. 4E), and the AIMD-based σ (Fig. 4F). These results highlight the high versatility and exceptional accuracy of the IonNet model in identifying FICs from a vast chemical composition space.

**Fig. 5.**
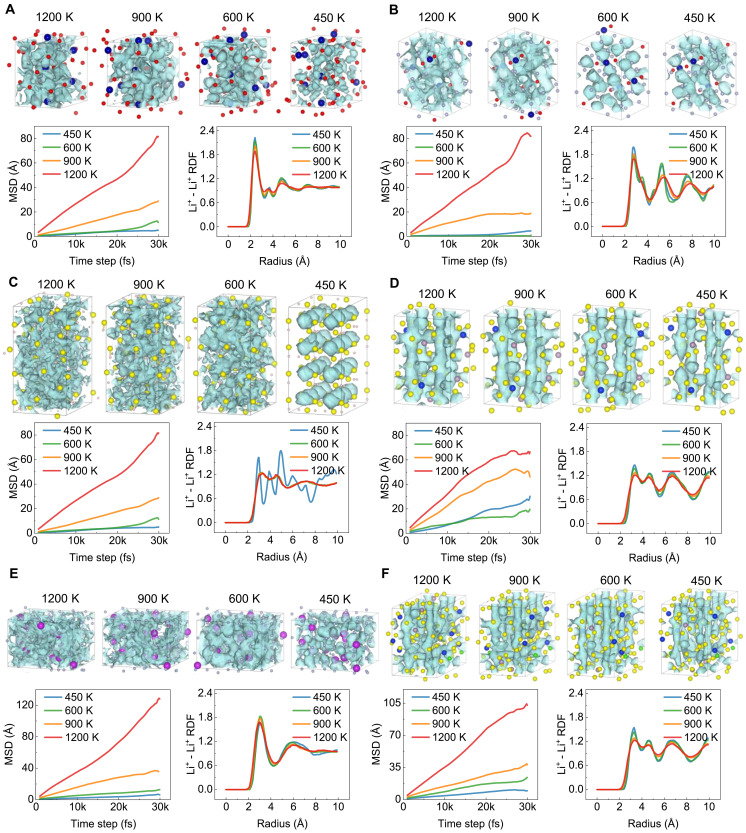
Validations of promising FICs via AIMD simulations. The three-dimensional migration path of Li^+^ and MSD over time at various temperatures is provided, together with the plots of RDF of Li^+^-Li^+^, which help analyze the average distance and short-range ordering between Li^+^ ions and evaluate the crowding within the Li^+^ migration channel. (**A**) Li_6_CoO_4_. (**B**) Li_4_CoOF_5_. (**C**) LiHS. (**D**) Li_10_SiP_2_S_12_. (**E**) Li_2_BiF_5_. (**F**) Li_20_Si_3_P_3_S_23_Cl. For the RDF curves, the cutoff was set to 10 Å and the bin width to 0.1 Å. For each compound, 1000 frames were randomly selected for calculation.

To better understand the temperature-dependent ion transport mechanism, we visualized the migration path of Li^+^ by plotting their mean square displacement (MSD) over time (Fig. 5). For Li_6_CoO_4_, Li_2_BiF_5_, and Li_20_Si_3_P_3_S_23_Cl, Li^+^ exhibits well-connected three-dimensional diffusion pathways across all investigated temperatures. This is further corroborated by the nearly linear increase in the MSD profiles, indicating sustained long-range diffusion behavior and the absence of kinetic trapping. However, for Li_10_SiP_2_S_12_, at 450 K, Li^+^ migration is predominantly confined along the *c* direction, suggesting a highly anisotropic diffusion network. Such one-dimensional transport pathways imply that Li^+^ migration is vulnerable to bottlenecks or blocking effects, which can hinder macroscopic σ despite the presence of locally accessible channels. This highlights the notion that the mere existence of diffusion pathways is insufficient; their dimensional connectivity critically governs effective ion transport. For LiHS, identified as a non-FIC, Li^+^ remains largely localized at 450 K, with migration restricted to short-range oscillations within local coordination environments. This behavior is characteristic of strong ion-framework interactions and high migration barriers, preventing the formation of percolating diffusion networks. These observations emphasize the critical role of diffusion dimensionality and connectivity in determining ionic transport properties. They provide important insights for the rational screening and design of SEs, where three-dimensional, percolating diffusion networks should be prioritized to achieve high σ in practical applications. More details of optimized structures and MSD results can be found in figs. S15 to S29.

Furthermore, the radial distribution function (RDF) of Li^+^-Li^+^ was plotted to analyze the average distance and short-range ordering between Li^+^ ions and to evaluate the crowding within the Li^+^ migration channel (Fig. 5 and figs. S15 to S29). At 450 K, these RDFs exhibit a pronounced first peak centered at ∼3.0 Å, signifying strong short-range ordering between Li^+^ ions. At higher temperatures, the first peak broadens and its intensity decreases slightly, indicating enhanced thermal vibration of Li^+^ and a concomitant weakening of local order. Notably, the RDF curves in some cases exhibit fluctuations or lack smoothness. This behavior can be attributed to multiple factors, including intrinsic structural heterogeneity (e.g., multiple inequivalent Li sites or diffusion pathways) as well as limited statistical sampling in AIMD simulations.

Last, we carefully evaluated the predicted compounds regarding synthesis feasibility and stability. From Fig. 4A, all candidates considered in this work were filtered with an *E*_hull_ below 0.1 eV per atom, a commonly adopted threshold indicating potential synthesizability. In addition, six of the predicted compounds [Li_6_CoO_4_, LiH_5_S(NO_2_)_2_, LiAlSiH_2_O_5_, LiHS, LiCaP_3_H_8_O_13_, and LiBiF_4_] already have entries in the Inorganic Crystalline Structure Database [ICSD (*62*); IDs: 62688, 15359, 40128, 98020, 68518, and 28567, respectively], providing direct experimental evidence of their realizability. For the remaining compounds without ICSD entries, we analyzed their possible decomposition pathways (corresponding to the energetically optimal phase assemblage on the convex hull, rather than a strictly balanced chemical reaction). For instance, Li_6_Ti_2_S_6_O is predicted to decompose into 7/9 Li_4_TiS_4_ (*E*_hull_ = 0), 2/15 Li_2_TiO_3_ (ID: 261238), 2/29 Li(TiS_2_)_2_ (*E*_hull_ = 0), and 1/51 TiS_3_ (ID: 42072), all of which are experimentally reported phases, which further supports its thermodynamic plausibility. Similar decomposition analyses were conducted (see below) for other candidates, suggesting that most of the predicted materials are either stable or metastable within experimentally accessible ranges.

$${\text{Li}}_{2}{\text{BiO}}_{3}\to 2/3\;{\text{Li}}_{3}{\text{BiO}}_{4}\;\left(\text{ID}:109087\right)+1/3\;{\text{LiBiO}}_{2}\;\left(E_{\text{hull}}=0\right)$$

$${\text{Li}}_{3}{\text{Al}}_{2}{\text{CoO}}_{6}\to 1/3\;{\text{LiCoO}}_{2}\;\left(\text{ID}:193442\right)+2/3\;{\text{LiAlO}}_{2}\;\left(\text{ID}:28288\right)$$

$${\text{Li}}_{4}{\text{Bi}}_{2}O_{5}\to 4/11\;{\text{LiBiO}}_{2}\;\left(E_{\text{hull}}=0\right)+7/11\;{\text{Li}}_{3}{\text{BiO}}_{3}\;\left(\text{ID}:40245\right)$$

$$\begin{array}{c} {\text{Li}}_{2}{\text{Bi}}_{2}P_{2}O_{9}\to 11/45\;{\text{Bi}}_{3}{\text{PO}}_{7}\;\left(E_{\text{hull}}=0\right)+2/5\;{\text{BiPO}}_{4}\;\left(\text{ID}:426238\right)+\\ 16/45\;{\text{Li}}_{3}{\text{PO}}_{4}\;\left(\text{ID}:10257\right) \end{array}$$

$${\text{Li}}_{2}{\text{BiF}}_{5}\to 3/4\;{\text{LiBiF}}_{4}\;\left(\text{ID}:65404\right)+1/4\;\text{LiF}\;\left(\text{ID}:52234\right)$$

$${\text{Li}}_{5}P{\left(S_{2}\text{Cl}\right)}_{2}\to 2/3\;{\text{Li}}_{3}{\text{PS}}_{4}\;\left(E_{\text{hull}}=0\right)+1/3\;\text{LiCl}\;\left(\text{ID}:26909\right)$$

$$\begin{array}{c} {\text{Li}}_{20}{\text{Si}}_{3}P_{3}S_{23}\text{Cl}\to 9/25\;{\text{Li}}_{2}{\text{SiS}}_{3}\;\left(E_{\text{hull}}=0\right)+12/25\;{\text{Li}}_{3}{\text{PS}}_{4}\;\left(E_{\text{hull}}=0\right)+\\ 3/25\;{\text{Li}}_{2}S\;\left(\text{ID}:642291\right)+1/25\;\text{LiCl}\;\left(\text{ID}:26909\right) \end{array}$$

$${\text{Na}}_{2}{\text{Li}}_{2}{\text{CoO}}_{4}\to 2/3\;{\text{Na}}_{2}{\text{CoO}}_{3}\;\left(E_{\text{hull}}=0\right)+{\text{Li}}_{2}O\;\left(\text{ID}:57411\right)$$

$$\begin{array}{c} {\text{Li}}_{4}{\text{CoOF}}_{5}\to 3/22\;{\text{CoO}}_{2}\;\left(E_{\text{hull}}=0\right)+8/11\;\text{LiF}\;\left(\text{ID}:52234\right)+\\ 3/22\;{\text{CoF}}_{2}\;\left(\text{ID}:53987\right) \end{array}$$

$${\text{CsLi}}_{2}F_{3}\to 2/3\;{\text{CsLiF}}_{2}\;\left(\text{ID}:245965\right)+1/3\;\text{LiF}\;\left(\text{ID}:52234\right)$$

$$\begin{array}{c} {\text{NaLi}}_{5}N_{2}\to 1/18\;{\text{NaN}}_{3}\;\left(\text{ID}:155167\right)+5/6\;{\text{Li}}_{3}N\;\left(\text{ID}:642177\right)+\\ 4/9\;\text{Na}\;\left(\text{ID}:44758\right) \end{array}$$

$$\begin{array}{c} {\text{Li}}_{4}{\text{Co}}_{2}{\text{OF}}_{7}\to 3/28\;{\text{CoO}}_{2}\;\left(E_{\text{hull}}=0\right)+4/7\;\text{LiF}\;\left(\text{ID}:52234\right)+\\ 9/28\;{\text{CoF}}_{2}\;\left(\text{ID}:53987\right) \end{array}$$

## DISCUSSION

In summary, our study highlights that chemical composition is an important feature of a large class of FICs. Many studies have attempted crystal structure prediction on the basis of chemical composition, underscoring the central role of chemical composition in determining the crystal structure and atomic arrangements. Static and statistical composition descriptors derived from elemental properties, as well as the composition descriptors learned from crystal GNNs, have demonstrated considerable predictive ability, thereby finding tens of thousands of potential FICs from more than 5 million compounds. Our IonNet framework provides insights into the physical properties that enable fast Li^+^ conduction, especially under the condition that the current crystal structure data are relatively scarce, which points to an exciting direction for accelerating the discovery of FICs for ASSBs.

Recent advances in structure-based ML models, such as CHGNet (*28*) and M3GNet (*31*), have improved the accuracy of property prediction by explicitly incorporating atomic coordinates and local bonding environments. However, these models require reliable crystal structures as inputs, which limits their applicability in many experimental settings where structural information is unavailable or uncertain. In contrast, the IonNet framework focuses directly on the composition-property relationship and can operate without prior knowledge of crystal structures. This feature enables rapid screening across vast compositional spaces and makes the approach particularly suitable for early-stage materials exploration, where composition is often the only available descriptor. Therefore, IonNet provides a complementary pathway to traditional structure-based models, bridging the gap between composition-driven discovery and structure-dependent prediction.

In this context, the advantage of IonNet is not limited to improved accuracy in σ prediction but also lies in its potential extensibility. By integrating multiple types of descriptors within a unified representation and leveraging TL, IonNet captures compositional and physicochemical information that is not inherently restricted to a specific property. This suggests that the framework can be naturally extended to other SE properties, such as electronic structure descriptors or thermomechanical properties, without fundamental modification of the model architecture.

Furthermore, IonNet could be improved in several aspects. For instance, structure factors influencing ionic conductivity have been considered; a systematic analysis of structure is still limited because of the scarcity of available structural data. Potential strategies include using MLFFs to predict the crystal structures of substituted compositions or applying generative models to produce atomic coordinates conditioned on chemical compositions. Such approaches are expected to more effectively couple compositions and structure with ion transport properties. Another promising direction is the development of high-accuracy MLFFs to fully or partially replace AIMD simulations, thereby accelerating the validation process. The validated results can be incorporated back into the dataset, enabling a closed-loop active learning cycle.

Overall, our study establishes a high-precision prediction platform for evaluating ionic conductivity directly from chemical compositions. The distinctive network architecture of the proposed descriptors, together with the GBA interpretability method, offers perspectives for property prediction across different modalities and descriptors while also enhancing the chemical interpretability of the results.

## MATERIALS AND METHODS

### Data processing

The computational datasets were obtained from the IonML platform (the standardized tabular dataset containing 8750 samples for model training is provided in data S1), which integrates an efficient classification model achieving a high prediction accuracy of 90.4% for identifying FICs. IonML’s training data combine experimental and AIMD results using features such as structural features (space group and lattice symmetry), chemical composition (chemical fraction), and Li^+^ site attributions (effective ratio). The dataset comprises 19,209 data points, including structural and chemical compositional details as well as classification probabilities for FICs. Given that this study uses chemical composition to describe the Li^+^ conductors, identical chemical formulas were consolidated, with their probability values averaged, resulting in 8750 unique entries. For the computational dataset, because of its large scale, the model’s performance was evaluated by conducting 10 random training iterations and averaging the results. The dataset was split into a training set and a test set with a ratio of 9:1 (random seed: 42) and a training batch size of 256.

The experimental data were sourced from literature reports, focusing on ionic conductivity measurements at room temperature (15° to 35°C) for model training (*40*). As accurate crystal structure data are not mandatory, nearly all experimental data are usable, requiring only unique chemical compositions. Similarly, the average principle was applied, resulting in a dataset of 398 experimental entries (table S1 and data S2). For the experimental dataset, 10-fold cross-validation with a batch size of 32 and a random seed of 42 was used to assess the model’s performance comprehensively. We shuffled the training data to ensure that the model does not learn any unintended, order-dependent patterns. This helps eliminate potential biases and enhances the model’s generalization capabilities.

### Descriptor-specialized NN

Inspired by the chemical potential energy surface model, which treats potential energy as the sum of the contributions from the local chemical environment around each atom (*35*), here, we similarly interpret the ionic conductivity as the aggregated contribution of multiple descriptors. These descriptors are individually processed through independent self-attention network modules to extract high-order feature representations, which are then summed to produce the final ionic conductivity. This approach is more robust than traditional voting or ensemble models, as the integrated loss function of the entire network effectively captures the interdependencies between different descriptors. In this study, three representative descriptors, *m*_g_, *m*_e_, and *m*_t_, were selected to compose the descriptors and construct the models. See more details in Supplementary Text.

1) *m*_g_ descriptor: The *m*_g_ descriptor is a 120-dimensional vector (*36*), with an atomic fraction of each of the first 103 elements, in order of atomic number, and 17 statistics of elemental properties: mean atomic weight of constituent elements (one dimension), mean periodic table raw and column number (two dimensions), mean and range of atomic number (two dimensions), mean and range of atomic radius (two dimensions), mean and range of electronegativity (two dimensions), mean number of valence electrons in each orbital (four dimensions), and fraction of total valence electrons in each orbital (four dimensions).

2) *m*_e_ descriptor: *m*_e_, the materials-agnostic platform for informatics and exploration, is a versatile tool designed to streamline the development of ML models from materials data (*37*). Its broad scope enables the representation of a diverse range of physical and chemical properties, facilitating the creation of accurate models for various materials-related challenges. In this study, 22 elemental property attributes (i.e., melting temperature, covalent radius, volume, bandgap energy, magnetic moment, and space group) combined with six statistical operations (minimum, maximum, range, mean, mean absolute deviation, and mode) were used, yielding a 132-dimensional *m*_e_ descriptor vector for each compound.

3) *m*_t_ descriptor: We further incorporated nonlinear element embeddings generated using the materials graph network (MEGNet) (*31*). These embeddings were trained on ∼60,000 structures from the MP database to predict formation energies. Consequently, the *m*_t_ descriptor should not be interpreted as extracting compound-specific structural information directly from composition. Rather, it provides a composition-level representation enriched with structure-informed elemental priors derived from large-scale crystal structure learning. Each element in the *m*_t_ is represented by a 16-dimensional vector. To derive compound-level descriptors, five statistical operations (minimum, maximum, range, mean, and standard deviation) were applied to these vectors, resulting in an 80-dimensional vector for each compound.

### Self-attention NN

The self-attention mechanism is a fundamental component of the Transformer model in deep learning (*63*, *64*). It captures global information by calculating the correlations among different elements using the input *V*, *K*, and *Q*. Here, descriptor encoding is essentially a sequence feature vector. To capture long-range dependencies, this study uses a fully connected linear layer to project the input features into the *Q*, *K*, and *V* spaces. The mechanism computes the similarity between the *Q* and *K* through dot products to derive attention weights, which are then used to weight the *V* and produce the final output

$$\text{Attention}\left(Q,K,V\right)=\text{softmax}\left(\frac{QK^{T}}{\sqrt{d_{k}}}\right)V$$ (1)

where $\sqrt{d_{k}}$ is the scaling factor used for normalization.

Before the self-attention mechanism module, three fully connected hidden layers are used to progressively project and nonlinearly transform the inputs, generating higher-dimensional features with enhanced expressiveness for subsequent processing. Nonlinear rectified linear unit activation functions are applied between layers to further boost the model’s representational capacity. As a preprocessing step of the self-attention mechanism, this module can be optimized independently, allowing easier debugging and testing. Following testing, the finalized network architectures are as follows: (120-48-16-16) for the *m*_g_ NN, (132-48-16-16) for the *m*_e_ NN, and (80-48-16-16) for the *m*_t_ NN.

The generated *Q*, *K*, and *V* are fed into a single-head attention module to compute the weighted feature output, preserving the global dependency information of the input features. Given that *m*_g_, *m*_e_, and *m*_t_ are independently processed by three NNs, the use of single-head attention enables lightweight handling of small-scale feature data, making it well suited for tasks with smaller embedding dimensions. We further evaluated the effect of the number of attention heads (two, four, and eight; fig. S30). The results show that the performance differences on the source dataset are marginal. On the target experimental dataset, however, the single-head attention achieves the best performance. This may be attributed to the relatively small embedding dimension (*16*) and the limited size of the target dataset, where excessive head splitting may reduce the expressive capacity of each head and introduce additional parameters that lead to overfitting. In addition, the interactions among the input features are relatively weak in terms of long-range contextual dependency, making single-head attention sufficient to capture the dominant feature relationships.

### TL procedure

The TL strategy was adopted on the experimental dataset to improve the model’s performance and reduce the risk of overfitting. The parameters from the best-performing source model among the 10 trained on the computational dataset were selected as the initialization parameters for the target model. To reduce the number of parameters requiring optimization, we fixed the parameters of the third fully connected layer on the basis of testing results, allowing only the first two layers to be optimized. The source model was trained with a learning rate of 0.002 for 1000 epochs. For the target model, the learning rate was reduced to 0.0015, and the batch size was set to 16 to enable fine-tuning, with training conducted over 300 epochs. Checkpoints were established at each epoch to save the parameters corresponding to the best model performance. These strategies all help to reduce the impact of overfitting on model performance. To observe these results more clearly, we supplemented the performance on the training sets (see table S3).

### Gradient-based attribution

Owing to the implementation of the IonNet model, a three-parallel attention NN framework, visualizing the decision-making process is more challenging compared to tree-based models. Interpretability approaches can be broadly categorized into model-specific methods and model-agnostic methods (such as Shapley additive explanations). Model-agnostic methods estimate feature contributions by perturbing inputs or approximating Shapley values, which provides a more global and theoretically grounded attribution. However, these approaches typically incur substantial computational cost, especially for high-dimensional inputs, as the number of model evaluations scales with the number of features. In contrast to model-agnostic interpretation methods and permutation importance, GBA directly leverages the internal structure of the trained model and can be efficiently evaluated via backpropagation. Therefore, to identify the key chemical factors influencing the σ, a GBA method was used to evaluate feature importance (*45*). For each subnetwork, the gradient (Δσ/Δ**X***_i_*) of the network output with respect to a specific input feature vector **X***_i_* was computed. The element-wise product of the gradient and the feature value was then calculated as an indicator of the feature importance.

Nevertheless, GBA remains a local attribution method, as it depends on gradients computed at specific inputs and may be sensitive to model smoothness and gradient saturation. Therefore, the feature importance derived from GBA should be interpreted as reflecting local sensitivities rather than globally averaged contributions. By balancing computational efficiency with interpretability, we adopted GBA as a practical and scalable approach for analyzing feature contributions within high-dimensional descriptors.

### AIMD simulation

Geometric structures were first optimized using generalized gradient approximation before performing AIMD simulations (see the information on supercell size in Supplementary Text) (*65*). The AIMD simulations used gamma-only *k*-point sampling with a plane-wave energy cutoff of 400 eV. The system was heated to the target temperature within 5.0 ps using velocity scaling, followed by equilibration at the target temperature for an additional 5.0 ps in the NVT ensemble (i.e., constant atomic number, volume, and temperature) controlled by a Nosé-Hoover thermostat (*66*, *67*). For the unknown FICs, fully converged AIMD simulations sampled diffusion events over 30.0 ps (with a time step of 2.0 fs) per temperature in the NVT ensemble until the diffusion coefficients stabilized. Simulations were conducted at three distinct temperatures (i.e., 1200, 900, 600, and 450 K) for each compound, enabling a linear fit to the Arrhenius relation (*68*). The diffusion coefficient was determined from the MSD following established methodologies, while Li^+^ conductivity was calculated using the Nernst-Einstein relation. Both structure relaxation and AIMD simulations were conducted with the Vienna Ab initio Simulation Package (*69*).
