# Supplementary material for: Decoding the chemical space of fast-ion conductors via a descriptor-guided transfer learning framework
Source: Sci Adv. 2026 Aug 7;12(32):eaee4959. doi: 10.1126/sciadv.aee4959 (PMC13450367; doi:10.1126/sciadv.aee4959)
Supplement: Supplementary file 1 [file sciadv.aee4959_sm.pdf]

Science Advances
AAAS

Supplementary Materials for

Decoding the chemical space of fast-ion conductors via a descriptor-guided transfer learning framework

Zhilong Wang and Fengqi You

Corresponding author: Fengqi You, fengqi.you@cornell.edu

Sci. Adv. **12**, eaee4959 (2026)
DOI: 10.1126/sciadv.aee4959

The PDF file includes:

Supplementary Text
Figs. S1 to S30
Tables S1 to S9
Legends for data S1 and S2

Other Supplementary Material for this manuscript includes the following:

Data S1 and S2

Supplementary Text

Complementary in nature to the three descriptors

Theoretically, as illustrated in the **fig. S1**, m_g is constructed from a 103-dimensional vector corresponding to the elements in the periodic table, which directly encodes the chemical composition of a compound. This representation is further supplemented by 17 statistical descriptors derived from elemental properties. In contrast, m_e is entirely composed of statistical descriptors of elemental properties and incorporates a broader range of physicochemical attributes, such as melting temperature, covalent radius, and atomic volume, thereby complementing the information captured by m_g.

Although a small number of elemental properties may overlap between m_g and m_e, their influence on the model is limited because the two descriptors are processed by separate subnetworks. This multi-channel architecture enables the model to preserve modality-specific representations while mitigating potential information redundancy or dilution. The descriptor m_t, however, differs fundamentally from m_g and m_e. It was derived from a pre-trained graph neural network, providing structure-informed elemental representations that were learned from large crystal structure datasets. Therefore, m_t captures a distinct type of information compared with the composition- and property-based descriptors.

From the perspective of IonNet's predictive performance, particularly the results of the 16 ablation experiments (**Fig. 2** and **Fig. 3a–b**), combining the three descriptors significantly improves model accuracy. This result highlights the strong complementarity among m_g, m_e, and m_t in capturing different aspects of materials information.

Inspiration for designing multi-channel networks

Predicting the properties of materials based solely on chemical composition has broad application prospects and is even a necessary research method at present. On the one hand, chemical composition has been proven to be able to predict crystal structure, which shows the potential mapping relationship between chemical composition and crystal structure, which is similar to predicting three-dimensional structure from protein sequence information; on the other hand, if we want to establish a database and build an AI model through experimental technology, the most scarce information at present is the specific information of the crystal structure, that is, the atomic coordinates, and a large amount of chemical composition information has not been fully utilized.

There are some studies on descriptors based on chemical composition, such as new material mining in spinel, garnet, and other systems. In this study, we selected three mainstream descriptors: meredig (m_g)(*36*), magpie (m_e)(*37*), and MEGNet (m_t)(*31*). The first two start from chemical composition and can calculate the static and statistical properties of elements, fully mining the information between the elements and chemical proportions that make up the compound; while the latter comes from the graph embedding expression trained on a large crystal structure database. The calculated features are more nonlinear and can have structure-informed elemental priors.

As for how to fuse these three features, we were inspired by a neural network model based on the atomic-centered potential energy surface (PES)(*35*), which regards the potential energy as the sum of the contributions of the local chemical environment of all atoms. We also introduced the three features into three self-attention network models, respectively, and fused them at the output (**fig. S2**). On the one hand, the three features were fully nonlinearly calculated before fusion to obtain high-order chemical information; on the other hand, this architecture is highly flexible and extensible, that is, new chemical descriptors can be easily added to improve the learning ability of

the model. Importantly, such a network structure can also ensure that each type of feature can be independently analyzed for interpretability, which is crucial for further investigating the impact of chemical composition on ionic conductivity. Compared to simple feature concatenation (see the figure above), the multi-channel architecture preserves modality-specific representations and avoids information dilution, thereby enabling synergistic interactions across features that are crucial for accurate property prediction.

Theoretical rationale of m_t for incorporating structure-informed elemental priors

In the MEGNet framework(*31*), each element Z is represented by a learnable embedding vector:

$$\mathbf{e}_Z \in \mathbb{R}^d \tag{S1}$$

These embeddings are optimized during the training of the graph neural network on a dataset of crystal structures S and their target properties y:

$$\min_{\theta,\mathbf{e}} \sum_{(S,y)} L\big(f_\theta\big(G(S,\mathbf{e})\big), y\big) \tag{S2}$$

where $G(S, \mathbf{e})$ denotes the graph representation of a crystal structure with node features initialized by elemental embeddings. Because the MEGNet model is trained on a large number of crystal structures with diverse bonding environments and coordination motifs, the learned elemental embeddings $\mathbf{e}_Z$ implicitly encode statistical correlations associated with the typical chemical environments of each element, including coordination preferences, bonding characteristics, and common structural roles across many compounds.

For a compound with composition $A_xB_yC_z$, the pretrained 16-dimensional m_t descriptors can be denoted as:

$$\mathbf{e}_A, \mathbf{e}_B, \mathbf{e}_C \in \mathbb{R}^{16} \tag{S3}$$

For the j-th embedding dimension:

$$\mathbf{e}_{A,j}, \mathbf{e}_{B,j}, \mathbf{e}_{C,j} \tag{S4}$$

The stoichiometry-weighted mean is:

$$m_j = \frac{x\mathbf{e}_{A,j} + y\mathbf{e}_{B,j} + z\mathbf{e}_{C,j}}{x+y+z} \tag{S5}$$

The minimum value is:

$$min_j = \mathbf{min}(\mathbf{e}_{A,j}, \mathbf{e}_{B,j}, \mathbf{e}_{C,j}) \tag{S6}$$

The maximum value is:

$$max_j = \mathbf{max}(\mathbf{e}_{A,j}, \mathbf{e}_{B,j}, \mathbf{e}_{C,j}) \tag{S7}$$

The range is:

$$range_j = max_j - min_j \tag{S8}$$

The unweighted standard deviation is:

$$s_j = \sqrt{\frac{(\mathbf{e}_{A,j} - \bar{e}_j)^2 + (\mathbf{e}_{B,j} - \bar{e}_j)^2 + (\mathbf{e}_{C,j} - \bar{e}_j)^2}{3}}, \bar{e}_j = \frac{\mathbf{e}_{A,j} + \mathbf{e}_{B,j} + \mathbf{e}_{C,j}}{3} \tag{S9}$$

Finally, the five statistical vectors are concatenated to an 80-dimensional vector. Importantly, this representation does not encode the actual crystal structure of a specific compound. In particular, the m_t descriptor does not contain explicit information about atomic coordinates, lattice geometry, local migration pathways, or Li-ion transport topology. Instead, the descriptor should be interpreted as incorporating structure-informed elemental priors learned statistically from large structural datasets during MEGNet pretraining.

For instance, Li frequently appears in highly coordinated ionic environments, while O or S commonly serves as framework-forming anions in many crystal structures. Such recurring structural tendencies are implicitly embedded in the learned elemental vectors through exposure to diverse structural datasets during training. Consequently, even when only compositional

information is available, the aggregated embedding representation can still capture meaningful statistical correlations related to likely chemical environments and bonding behaviors of the constituent elements.

Computational dataset

The Computational dataset with 8,750 data points was collected from the IonML platform developed in our previous work(*38*). IonML predictions are based on a new set of materials representations, which combine structural, Li^+, and component features. Structural features consist of crystal systems (cubic, hexagonal, trigonal, tetragonal, orthorhombic, monoclinic, and triclinic), centrosymmetric property, mean atomic volume, and mean number of atoms per volume. Site features are closely associated with the specificity of Li^+, including the mean number of neighboring atoms, mean two-atom length (MTL), and mean three-atom angle (MTA) between neighboring atoms and Li^+ for all Li^+ within a structure. Moreover, from the migration law of Li^+ within the structure, an appropriate channel size to accommodate Li^+ is necessary; thus, IonML uses the ratio of effective sites (RES) to evaluate the capacity to hold Li^+–that is, the probability that Li^+ placed randomly in a cell will not come into close contact with any known atoms in the cell, and the sum of the radii is less than their distance. Compositional features are calculated using the atomic number and fraction of metal and non-metal elements.

Since IonML is based on a small amount of experimental data, its primary function is to predict the probability that a material is a fast-ion conductor, making it a binary classification model. When performing high-throughput predictions on the MP database, IonML generates a large number of probability values for unknown materials. Compared to experimental techniques and DFT techniques, this prediction process is low-cost, scalable, and easily accessible, making it an excellent source of data for pre-trained models. A significant advantage is that the predicted data includes "knowledge" of the material's structure, Li^+ sites, and chemical composition, which can effectively inform downstream chemical composition-based predictions.

Details for calculating the mean and deviation of valence in orbitals

Here, taking compound $Sm_{0.52}Li_{0.38}TiO_{2.97}$ as an example, we show how to calculate the mean value and deviation of unfilled *f* valence orbitals. These two properties were used to describe the degree to which the properties of each compositional element in a compound deviate from their average values. First, the number of unfilled *f* valence orbitals (u) for each element should be calculated: [Sm: 8, Li: 0, Ti: 0, O: 0]. Then, the weighted average of all elements in the chemical formula by their mole fractions can be calculated:

$$mean = \frac{\sum(\omega \times u)}{W} \tag{S10}$$

where ω is the elemental mole fraction, and W is the total mole fraction. Therefore, the mean of (0.52×8+0.38×0+1×0+2.97×0)/(0.52+0.38+1+2.97)=0.854 can be calculated for $Sm_{0.52}Li_{0.38}TiO_{2.97}$. Deviation is the weighted average of the deviations of the feature values of each element from the average value according to its mole fraction:

$$deviation = \frac{\sum(\omega \times |u - mean|)}{W} \tag{S11}$$

As a result, the deviation of (0.52×7.146+0.38×0.854+1×0.854+2.97×0.854)/4.87=1.526 can be calculated for $Sm_{0.52}Li_{0.38}TiO_{2.97}$.

Ablation experiments

Based on different combinations of features, we conducted ablation experiments on computational and experimental datasets. For the three features, there are multiple combinations: single feature (m_g, m_e, or m_t) and two features ([m_g, m_e], [m_g, m_t], or [m_e, m_t]). In each experiment, the parameters of the NN were kept unchanged, i.e., (120-48-16-16) for the m_g NN, (132-48-16-16) for the m_e NN, and (80-48-16-16) for the m_t NN, in order to compare the performance under the same conditions. On the experimental dataset, ablation experiments were performed in both the from-scratch training and transfer learning modes (**Fig. 2**). These results all demonstrate the complementarity of the three features in expressing chemical composition and their excellent performance on the current dataset.

Details of theoretical validations

Both structure optimization and AIMD simulations were performed based on density functional theory using the VASP(*69*). In AIMD, supercells are used to simulate a larger portion of the material to capture more realistic interactions and properties, in particular, capturing long-wavelength vibrations and reducing periodic boundary artifacts. The number of atoms for the simulated systems are: Li_6CoO_4 (80 atoms), Li_2BiO_3 (96 atoms), $LiH_5S(NO_2)_2$ (104 atoms), $Li_3Al_2CoO_6$ (96 atoms), $Li_6Ti_2S_6O$ (120 atoms), $Li_4Bi_2O_5$ (132 atoms), $LiAlSiH_2O_5$ (120 atoms), $Li_2Bi_2P_2O_9$ (120 atoms), LiHS (96 atoms), Li_2BiF_5 (128 atoms), $Li_5P(S_2Cl)_2$ (96 atom), $CsLi_2F_3$ (46 atoms), $Li_4Co_2OF_7$ (56 atoms), $LiBiF_4$ (96 atoms), $Li_{10}SiP_2S_{12}$ (60 atoms), $Li_{20}Si_3P_3S_{23}Cl$ (100 atoms), $LiCaP_3H_8O_{13}$ (52 atoms), $Na_2Li_2CoO_4$ (72 atoms), Li_4CoOF_5 (120 atoms), $NaLi_5N_2$ (64 atoms). Geometric structures were first optimized using the generalized gradient approximation (GGA) before performing AIMD simulations.

Bond valence site energy analysis

We observed that the compounds exhibiting relatively large discrepancies (the ionic conductivity differs by approximately three orders of magnitude) between IonNet predictions and AIMD results include: $Li_5P(S_2Cl)_2$, Li_2BiO_3, $CsLi_2F_3$, and $Li_4Co_2OF_7$. We further analyzed the bond valence site energies (BVSE) for them (**fig. S15**).

For $Li_5P(S_2Cl)_2$ and $CsLi_2F_3$ the calculated 3D percolation barriers are (0.459, 0.488, 0.576 eV), and (0.703, 0.703, 1.182 eV), respectively, suggesting that Li^+ migration is effectively blocked in all three directions. Such extremely high barriers indicate the absence of viable diffusion pathways, consistent with the very low σ obtained from AIMD.

For Li_2BiO_3, the calculated 3D percolation barriers are (0.254, 0.254, 0.850 eV), indicating that Li^+ migration is significantly hindered along the *c* direction. The corresponding energy landscape further confirms the lack of a fully connected 3D diffusion network. This anisotropic and partially disconnected migration pathway leads to limited long-range diffusion, which explains why the AIMD-computed σ is lower than the model prediction.

For $Li_4Co_2OF_7$, the calculated 3D percolation barriers are (0.313, 0.352, 0.361 eV). Although the migration barriers in all three directions are relatively low, the corresponding energy landscape reveals distinct gaps along the *a* and *c* directions, indicating that Li^+ migration is hindered.

In addition, we also noted that compounds containing strong magnetism or heavy elements (such as Co and Cs) may further contribute to the observed discrepancies. These atoms can introduce complex electronic environments, including variable oxidation states and stronger covalent interactions with surrounding anions, influencing the AIMD simulations at high temperatures. These effects are not fully captured by the current model descriptors, potentially leading to reduced prediction accuracy for such systems.

Overall, the average deviation between the AIMD simulations and the IonNet predictions is only 1.415 orders of magnitude, which is well within the commonly accepted uncertainty range for σ calculations. This level of agreement highlights the robustness of IonNet and demonstrates its reliability for practical applications, particularly in high-throughput screening and comparative evaluation of candidate materials.

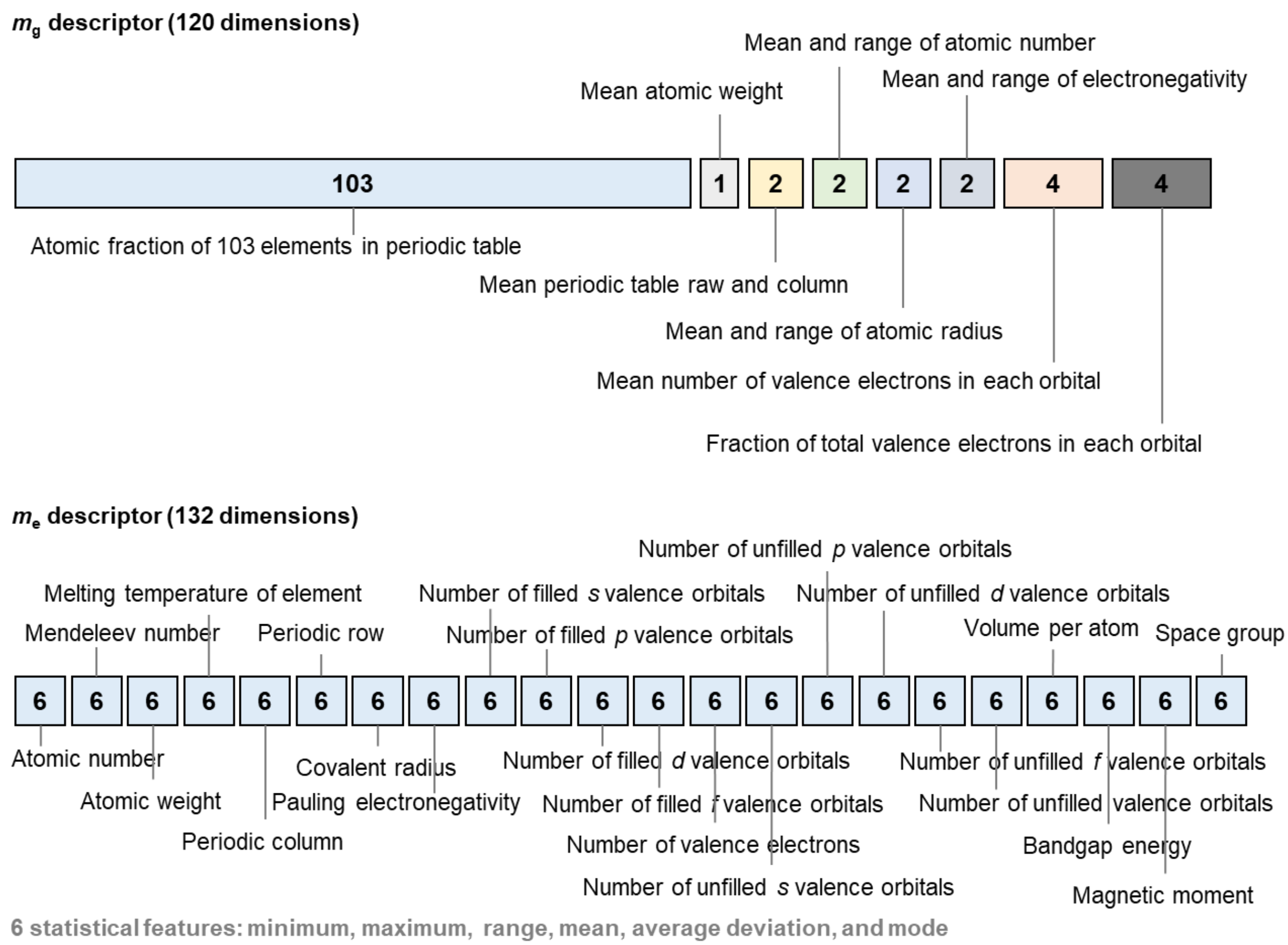

Fig. S1. Chemical meanings of m_g and m_e descriptors.

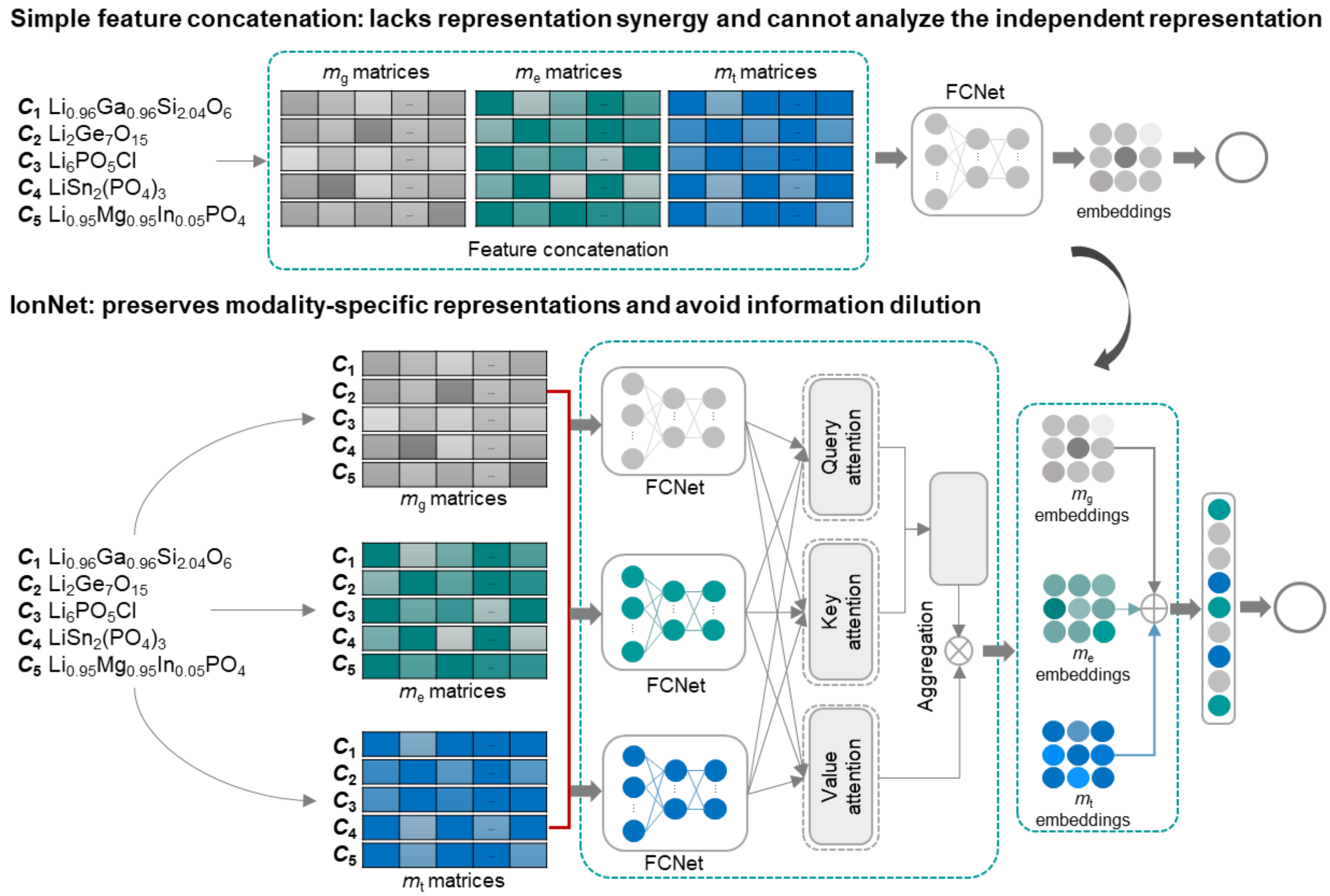

Fig. S2. Comparison between simple concatenation and multi-channel fusion. The FCNet means the fully-connected network.

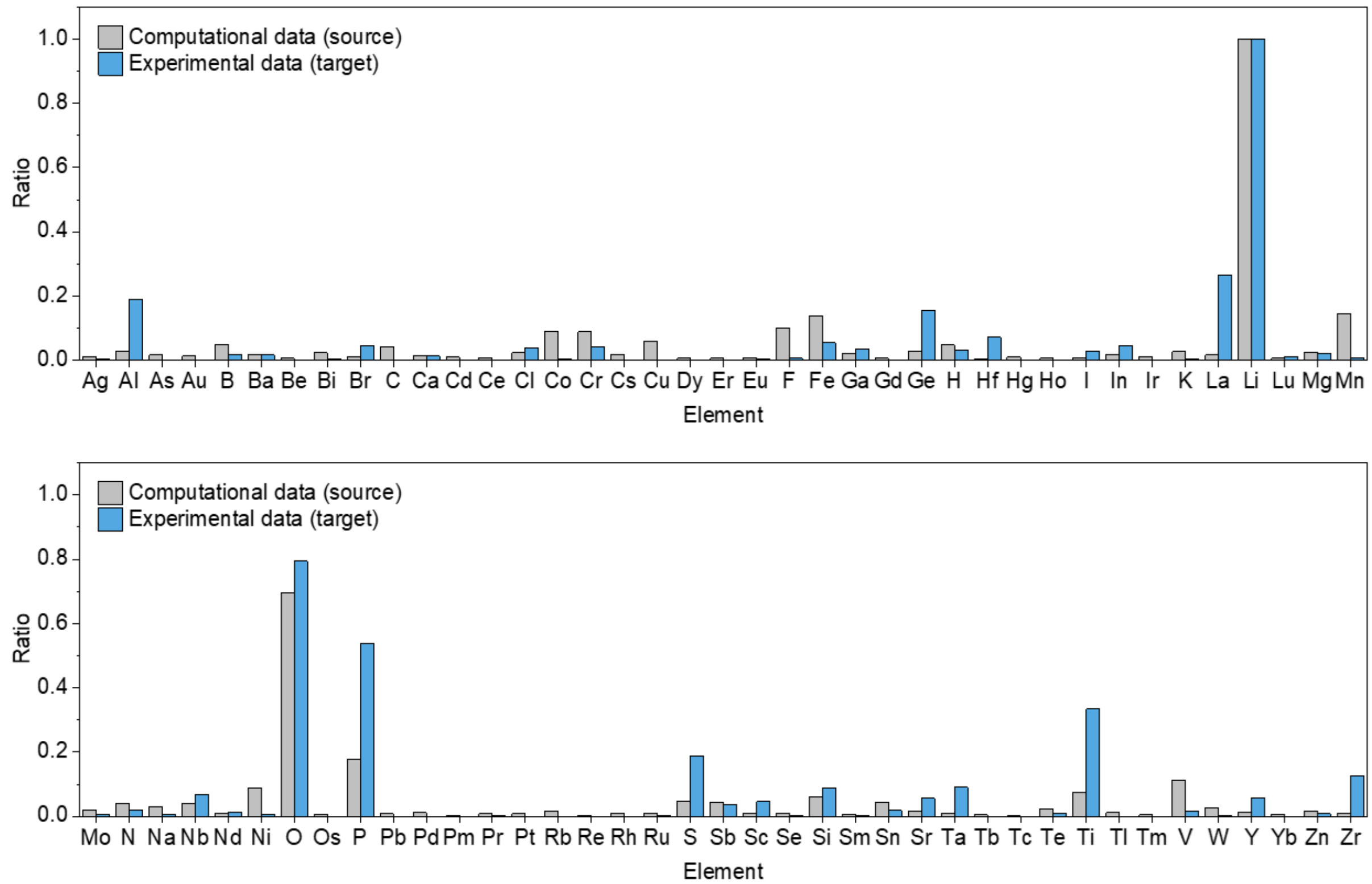

Fig. S3. Elemental frequency in the computational (source) and experimental data (target). As all the compounds contain Li^+, the ratio of Li is 100%. This indicates that the source and target datasets exhibit both structural similarities and systematic differences. On the one hand, both datasets are dominated by O-containing compounds, and P-containing compounds also represent a substantial fraction in both datasets, suggesting partial overlap in compositional categories. On the other hand, notable distinctions are evident. The target dataset contains a significantly higher proportion of La-containing compounds (such as $Li_{5.5}La_{2.75}K_{0.25}Nb_2O_{12}$ and $Li_{0.36}La_{0.55}TiO_3$), likely reflecting current experimental efforts that preferentially employ rare-earth elements for materials modification. In addition, the source dataset spans a broader elemental diversity. Several elements present in the source dataset, such as As, Au, and Be, are absent from the target dataset, indicating that the source data cover a wider chemical space. This broader compositional coverage may facilitate more effective model pretraining and provide a richer representation basis for subsequent fine-tuning on the target system.

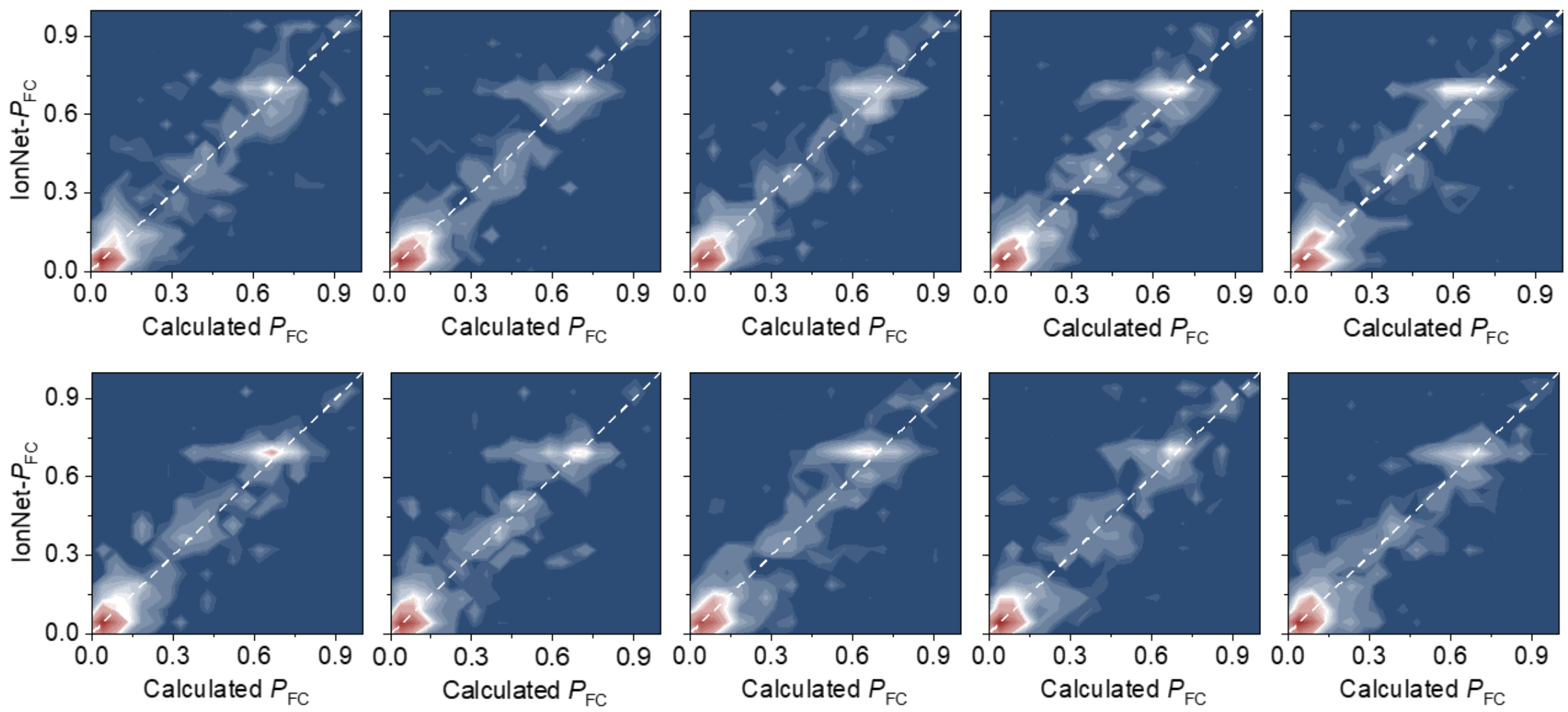

Fig. S4. Correlations of the computationally measured P_{FC} and model predictions. The validation sets of ten model trainings are shown here, and due to the large size of the data, a density map is used for presentation. Red areas represent high density, while dark blue areas represent low density.

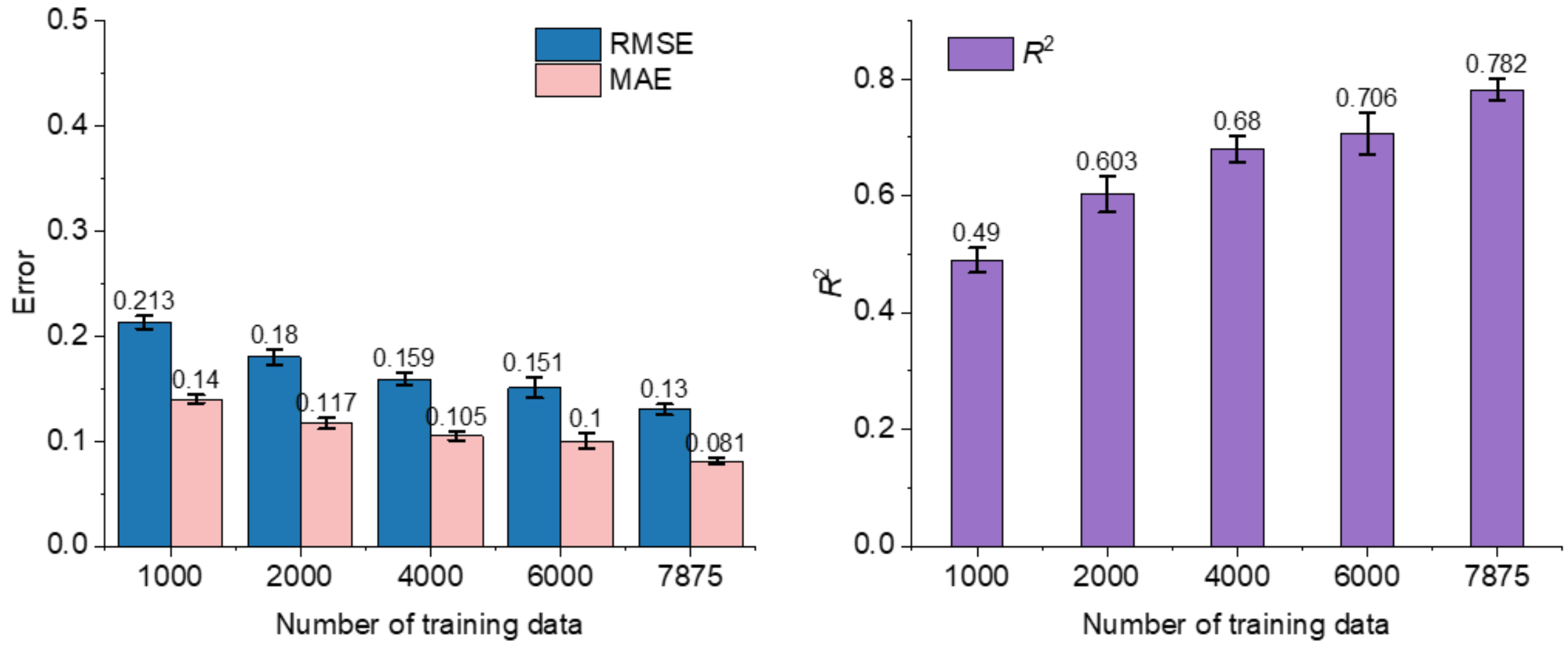

Fig. S5. Model performance with different computational data scales. Additional experiments using subsets of the source dataset with 1,000, 2,000, 4,000, and 6,000 samples were conducted. The model trained on the full dataset (7,875 for training and 875 for validation) achieves the best performance among all tested settings.

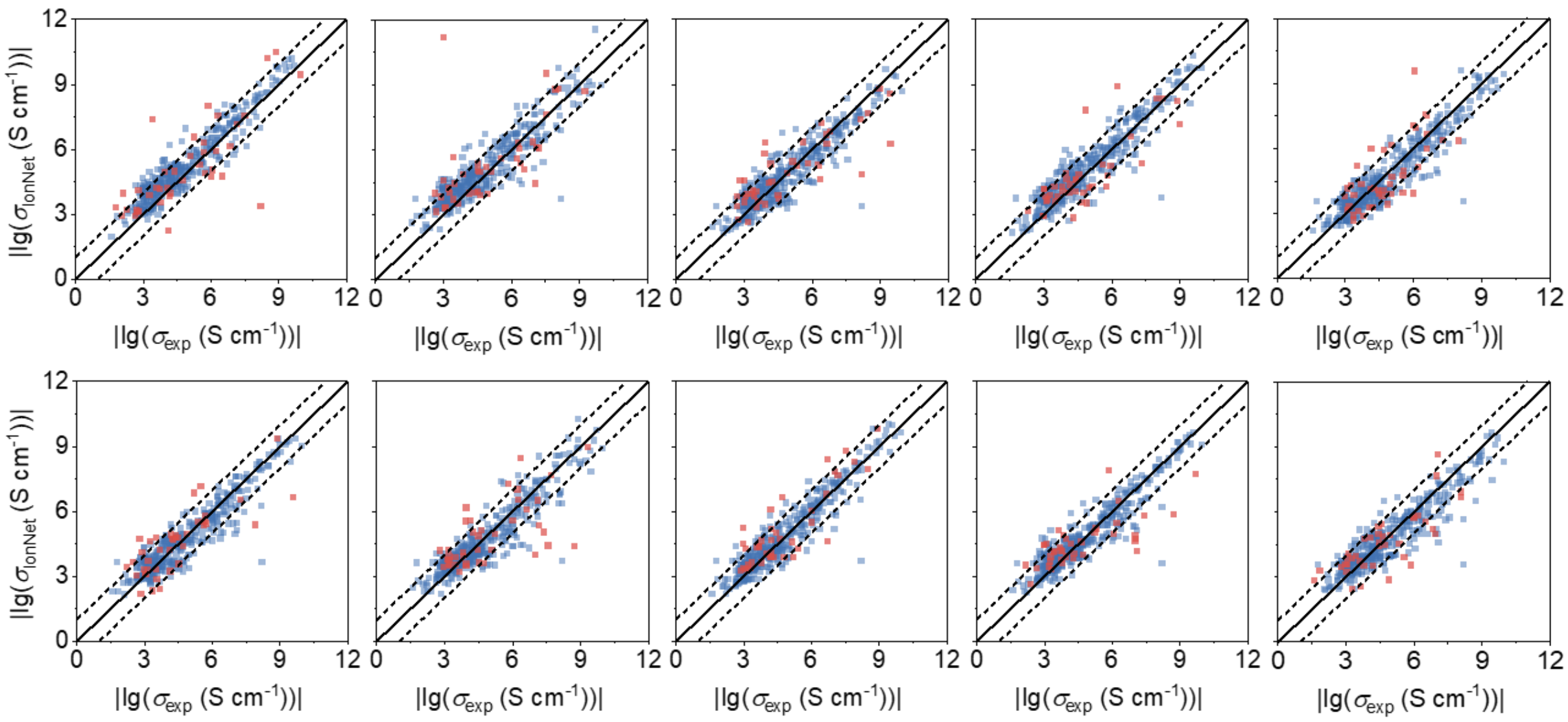

Fig. S6. Correlations of the experimentally measured σ and IonNet-TL predictions. The model is evaluated using ten-fold cross-validation, with blue representing the training set and red representing the validation set. The diagonal solid line indicates that the predicted value is equal to the experimental measurement value, while the dotted line indicates that the |lg(σ)| bias is 1.

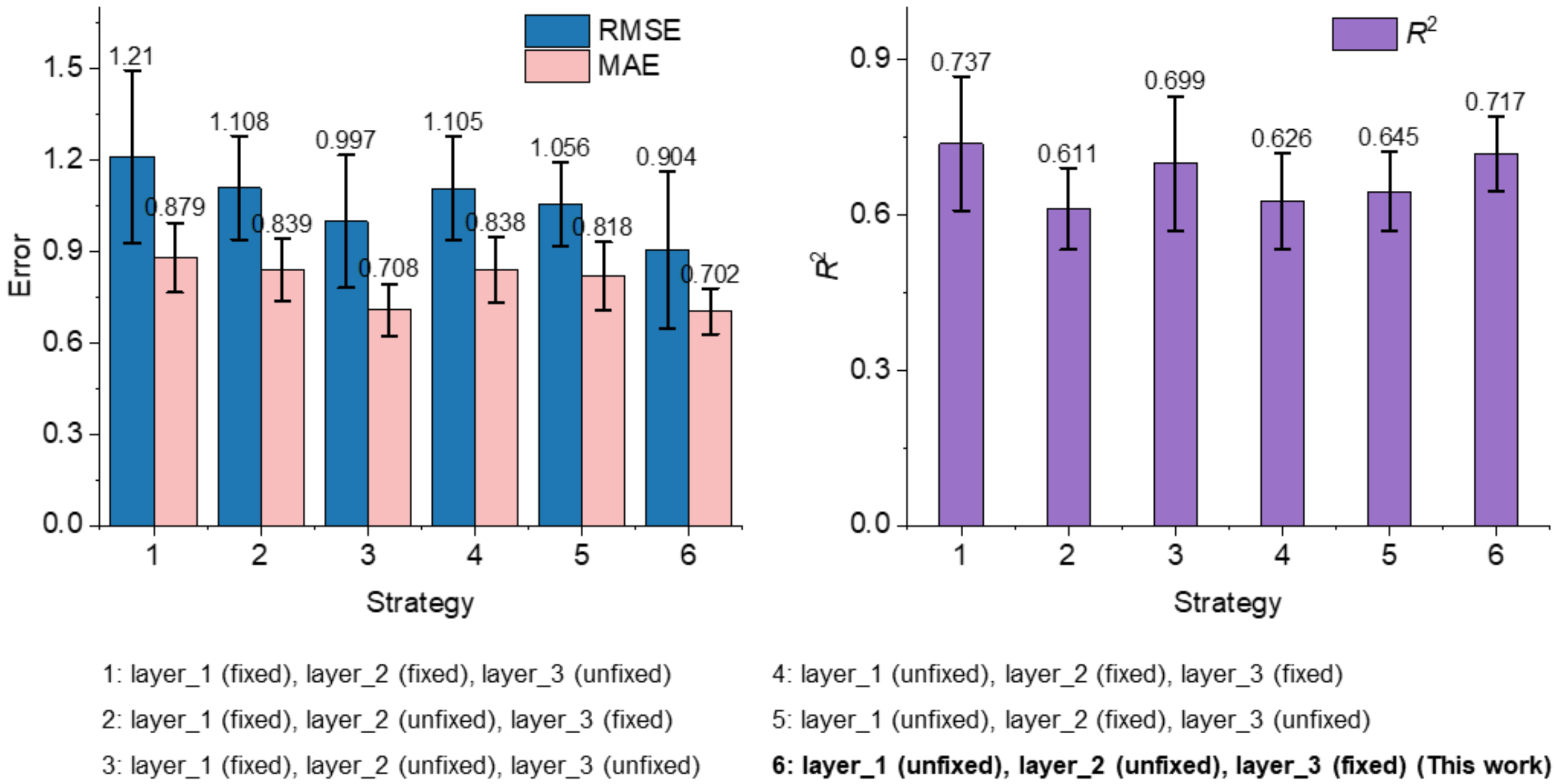

Fig. S7. Performance comparison of the IonNet-TL model under different network layer freezing strategies. The error bar means the standard deviation based on ten-fold cross-validation. In this work, there are three fully-connected layers (layer_1, layer_2, and layer_3). The relatively small performance differences among different freezing strategies suggest that the learned representations are not strictly separated into “general” and “task-specific” layers. Instead, higher layers appear to encode shared structure-property relationships that remain partially transferable across related tasks. This observation is consistent with the physics-driven nature of the problem, where different target properties are governed by overlapping underlying mechanisms.

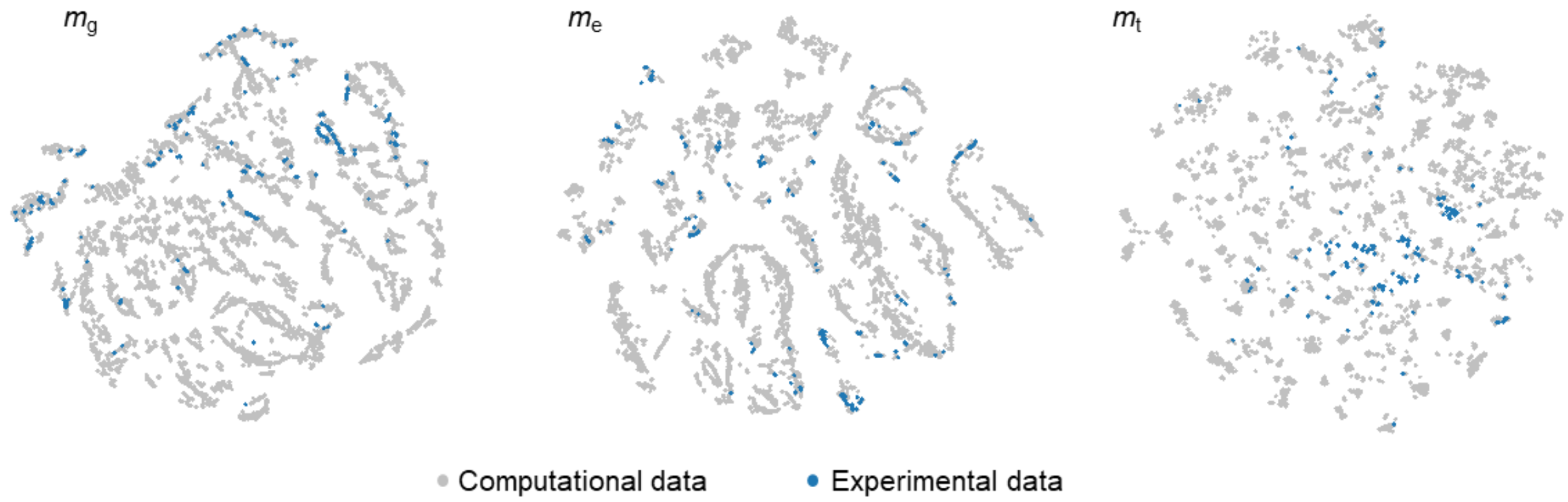

Fig. S8. t-SNE visualization of the combined computational and experimental data using descriptors (m_g, m_e, and m_t). The descriptor distributions of the computational and experimental domains exhibit a high degree of overlap overall. This indicates that the global distribution differences between the two domains in the high-level feature space are small, and the model has a good transfer foundation. In other words, fine-tuning in the source domain has a high probability of achieving good performance in the target domain, with minimal global shift. A small number of outliers exist in the experimental domain, independent of the computational domain distribution. This may stem from the fact that many experimentally synthesized materials contain elemental doping, but overall, the noise introduced is relatively small.

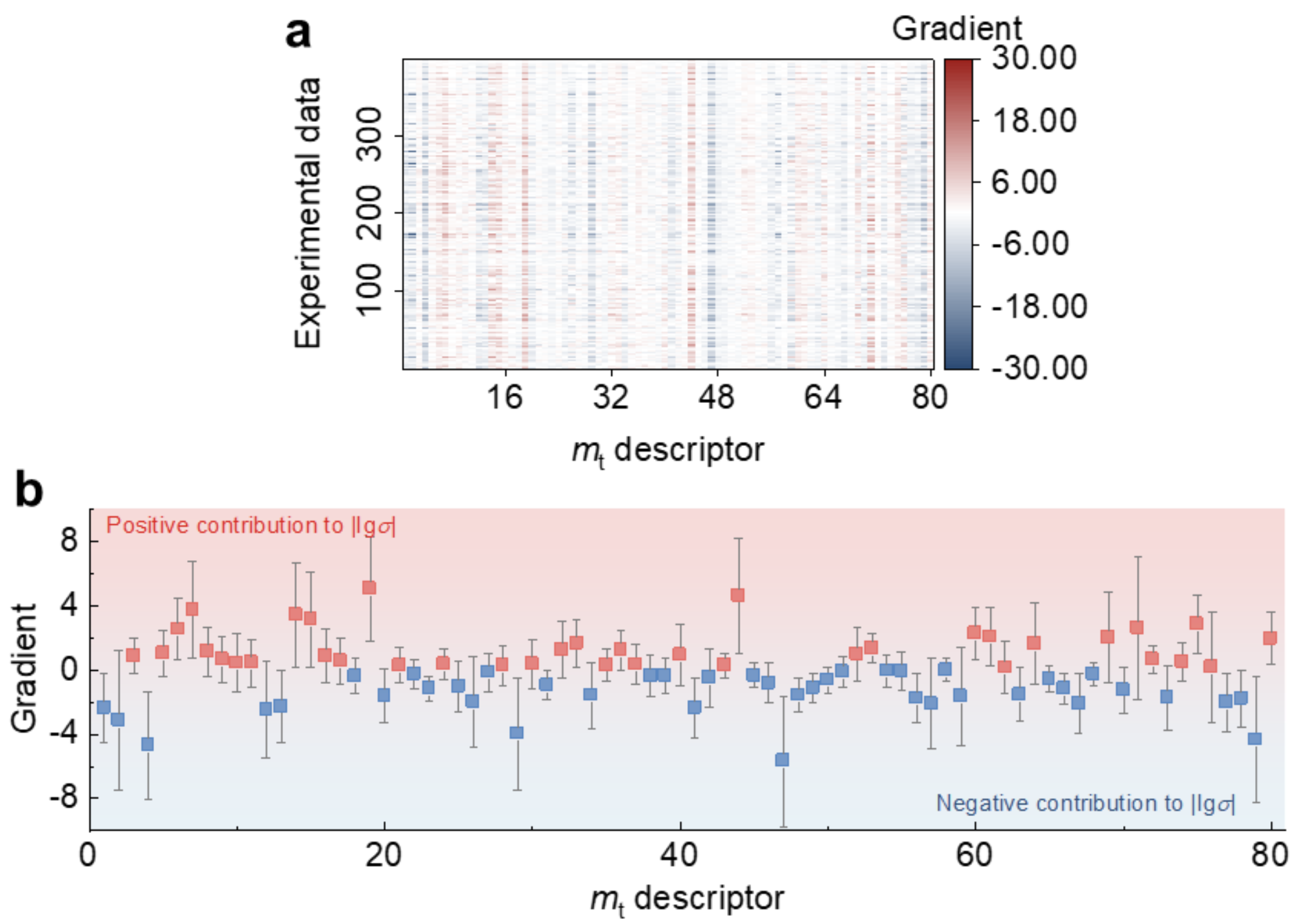

Fig. S9. Analysis of m_t descriptor by gradient-based attribution (GBA). a Gradient visualization of IonNet using the 80-dimensional m_t descriptor. **b** Average gradient (AG) of each feature of m_t, highlighting the features that IonNet prioritizes during the decision-making process.

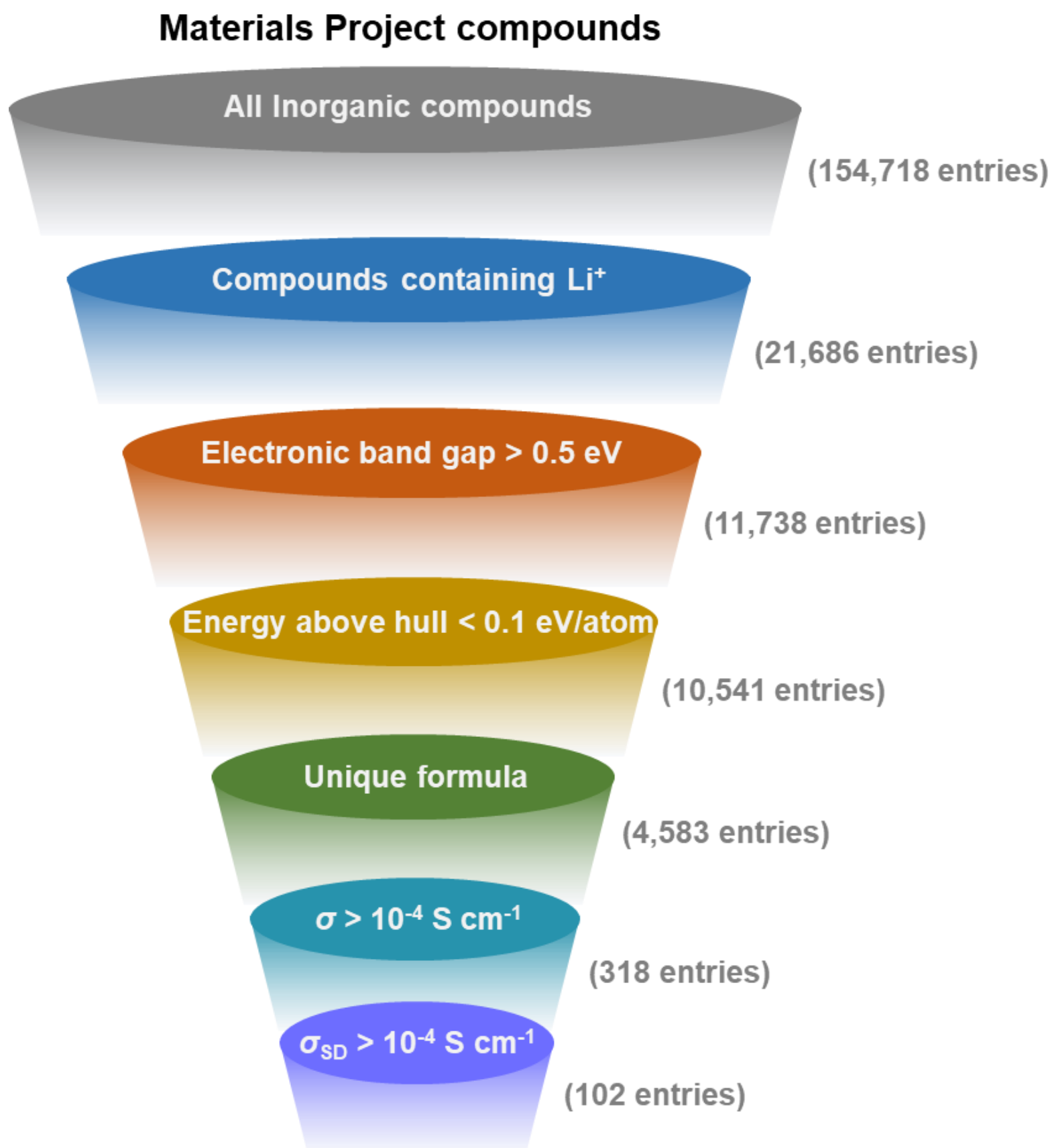

Fig. S10. Identifying fast-ion conductors from Materials Project via IonNet. A total of 154,718 compounds were retrieved from the Materials Project database (as of Oct/01/2024), among which 21,686 compounds containing Li^+ were identified. Considering the requirement for solid-state electrolytes to be electronically insulating, 11,738 compounds with a band gap greater than 0.5 eV were selected. Additionally, the thermodynamic stability of these compounds was evaluated, and 10,541 compounds with an energy above hull less than 0.1 eV/atom were chosen. To address the issue of polymorphism, 4,583 compounds with unique chemical formulas were further screened, ensuring that only the most thermodynamically stable structure was retained for each chemical formula. Subsequently, using the trained IonNet-TL model for high-throughput prediction, 318 compounds exhibiting ionic conductivity (σ) greater than 10^{-4} S cm^{-1} were obtained. To incorporate the uncertainty of model predictions and provide a direct reference for experimental validation, 102 compounds, whose predicted σ plus standard deviation (σ_{SD}) exceeded 10^{-4} S cm^{-1} were retained.

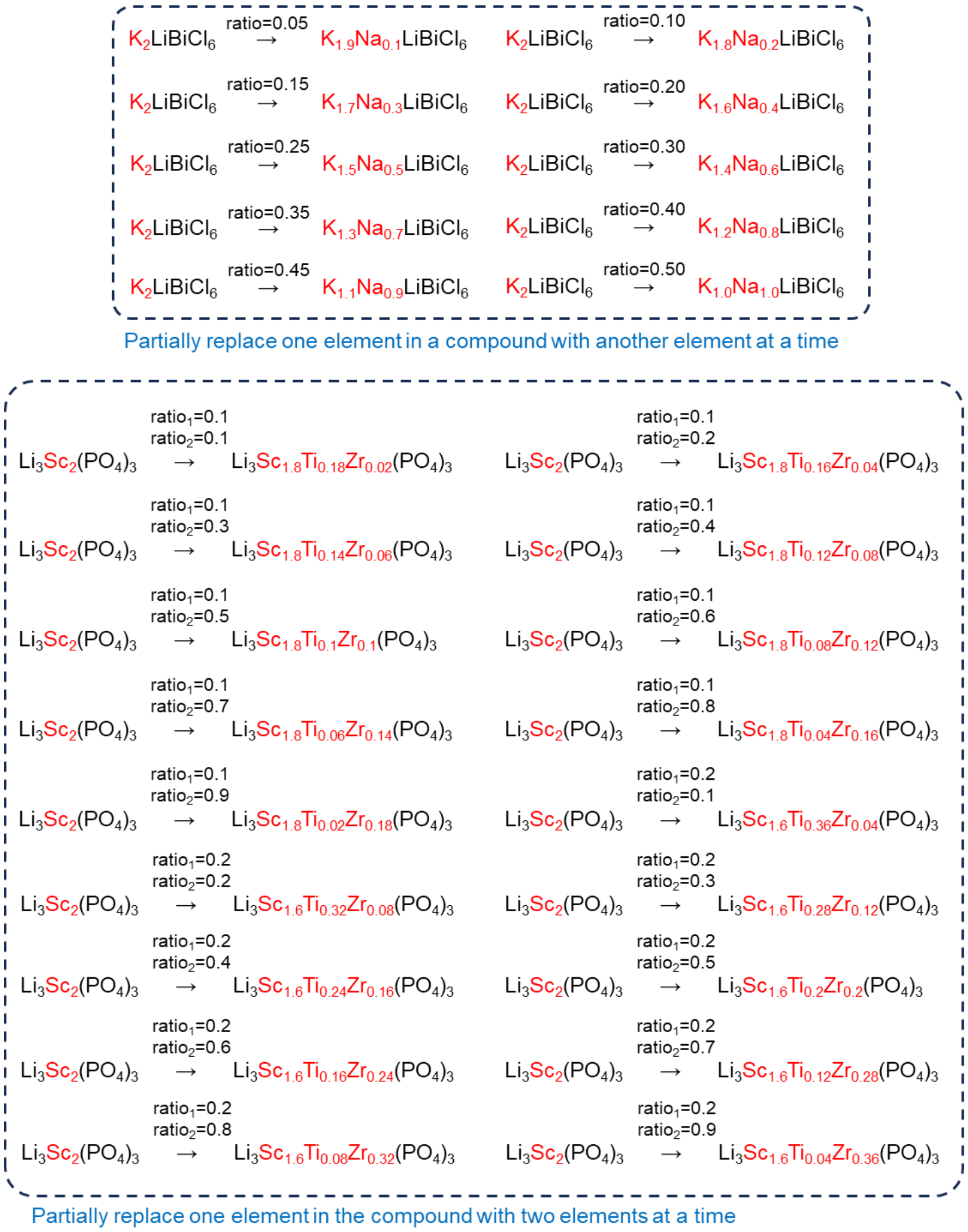

Fig. S11. Cases for single-element replacement and double-element replacement. We performed element replacement based on 4,583 data points selected from MP. Since the replacement was done with similar elements (similar radius or same group), we assumed that the crystal structure would not be significantly distorted. Two replacement methods were used in this study: single-element substitution (SES) - only one similar element was used to replace one element in the original chemical composition at a time, and double-element substitution (DES) - only two similar elements were used to replace one element in the original chemical composition at a time. For the SES procedure, the ratio of element replacement is 0.05, 0.1, 0.15, 0.2, 0.25, 0.3, 0.35, 0.4, 0.45, 0.5. For the DES procedure, the replacement step is divided into two steps. The first step is to select the ratio of elements to be replaced as 0.1, 0.2, 0.3, 0.4, 0.5. The second step is to assign the ratio of the two elements as 0.1, 0.2, 0.3, 0.4, 0.5, 0.6, 0.7, 0.8, 0.9.

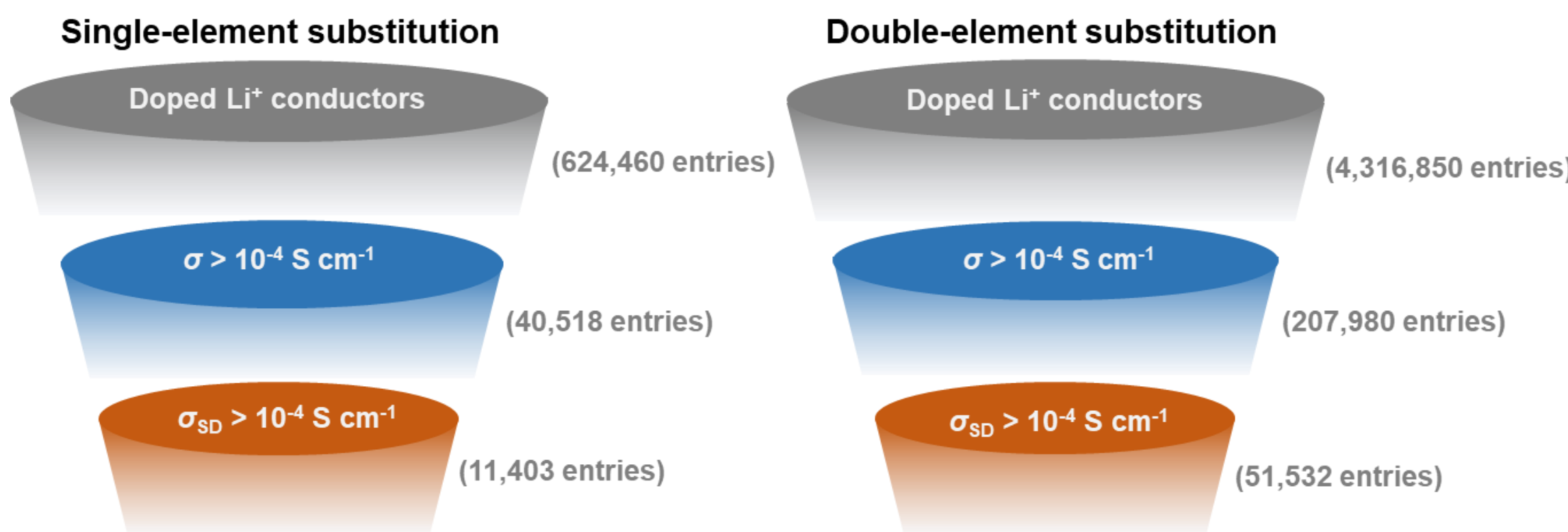

Fig. S12. Identifying fast-ion conductors from doped compounds via IonNet. A total of 4,583 compounds were generated from the Materials Project database after the screening of band gap and thermodynamic stability. Then, single-element substitution (partial substitution with one other element at a time) and double-element substitution (partial substitution with two other elements at a time), resulting in 624,460 and 4,316,805 compounds, respectively. Using the trained IonNet-TL for prediction and incorporating the standard deviation to account for uncertainty, we identified 11,403 and 51,532 potential fast-ion conductors from the single- and double-element substitution sets, respectively.

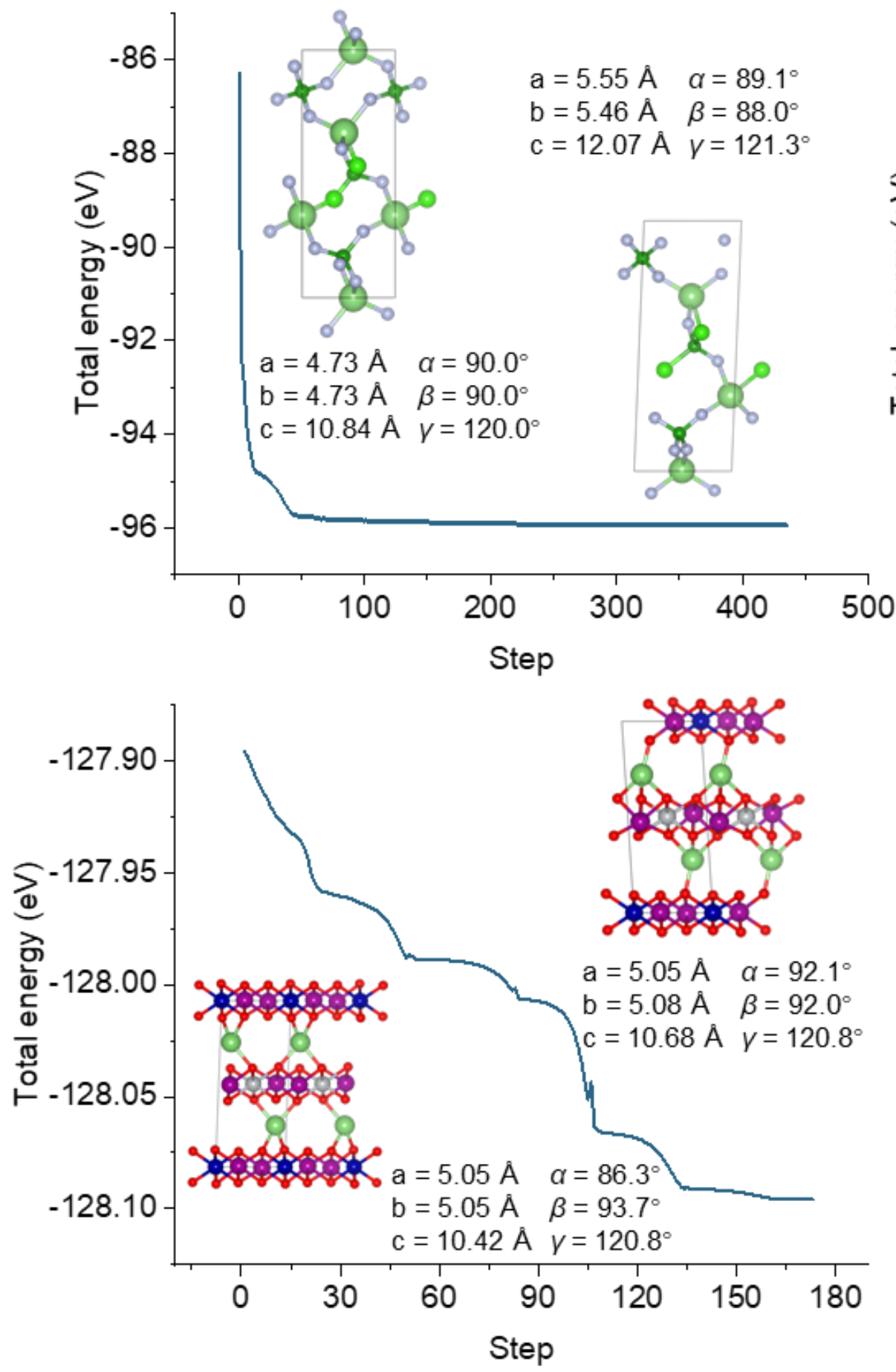

Fig. S13. Examples for structural optimization using MLFFs. Four compounds (mp-12403: $LiBF_4$ → $LiBF_2Cl_2$, mp-15999: Li_3AuSe_2 → Li_3AuSeS, mp-25284: $LiMn_2NiO_6$ → $LiMn_2Ni_{0.5}Co_{0.5}O_6$, mp-38280: $LiTiO_2$ → $LiTi_{0.5}Sr_{0.5}O_2$) were used to demonstrate the advantage of MLFFs. The M3GNet model trained on the Materials Project database (2021. 02) was employed as the MLFF backend. Users can directly utilize the MLFF scripts to perform preliminary structural optimizations, particularly for compounds predicted to be fast-ion conductors. For the 21,686 optimized structures compounds with well-defined crystal structures (i.e., non-doped, experimentally, or computationally established structures), structural optimization is not required, as the inputs are already structurally reliable. The data is available at https://doi.org/10.6084/m9.figshare.30141922.

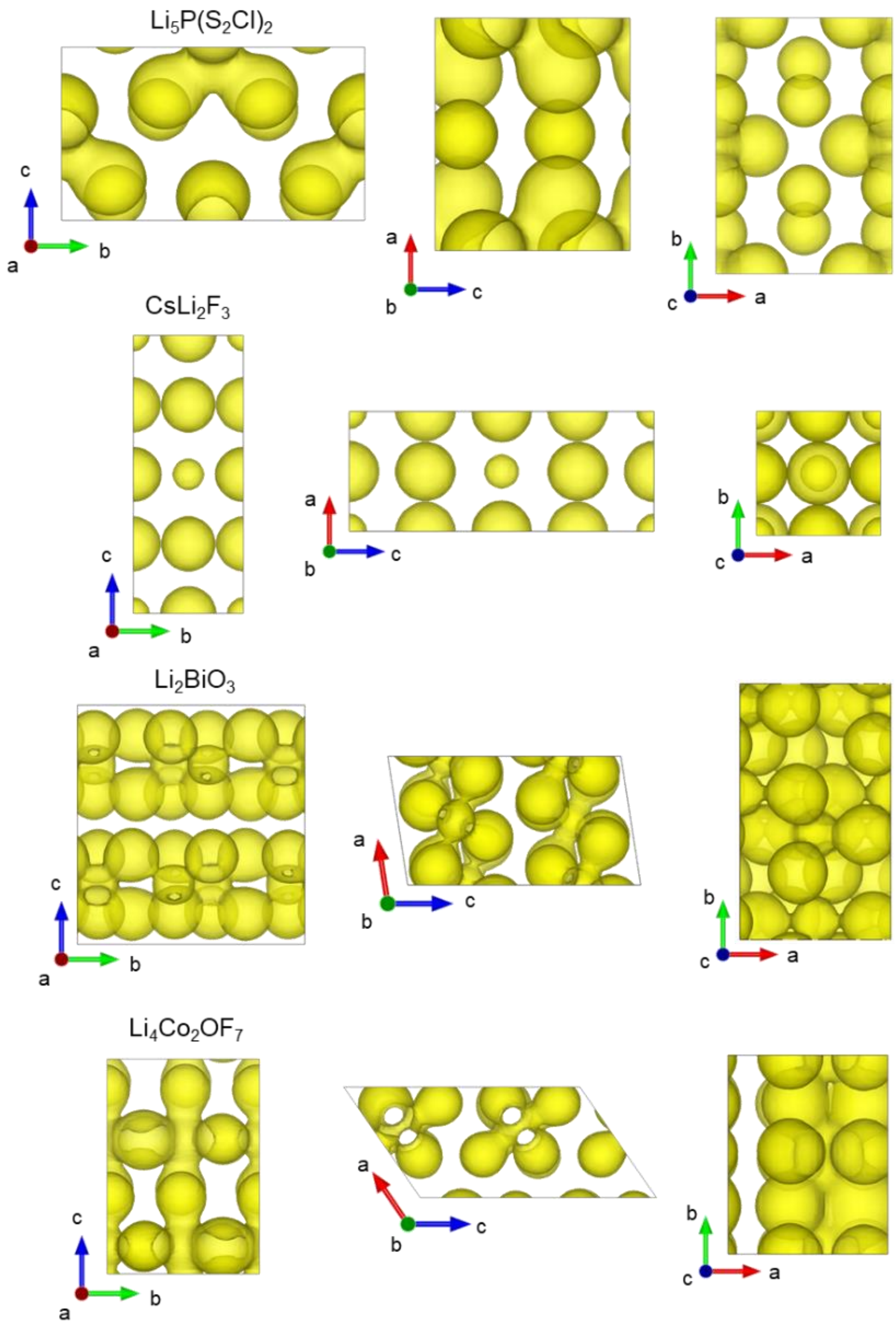

Fig. S14. BVSE analysis on four systems with relatively larger errors. For $Li_5P(S_2Cl)_2$, $CsLi_2F_3$, Li_2BiO_3, and $Li_4Co_2OF_7$, the calculated 3D percolation barriers are (0.459, 0.488, 0.576 eV), (0.703, 0.703, 1.182 eV), (0.254, 0.254, 0.850 eV), and (0.313, 0.352, 0.361 eV), respectively.

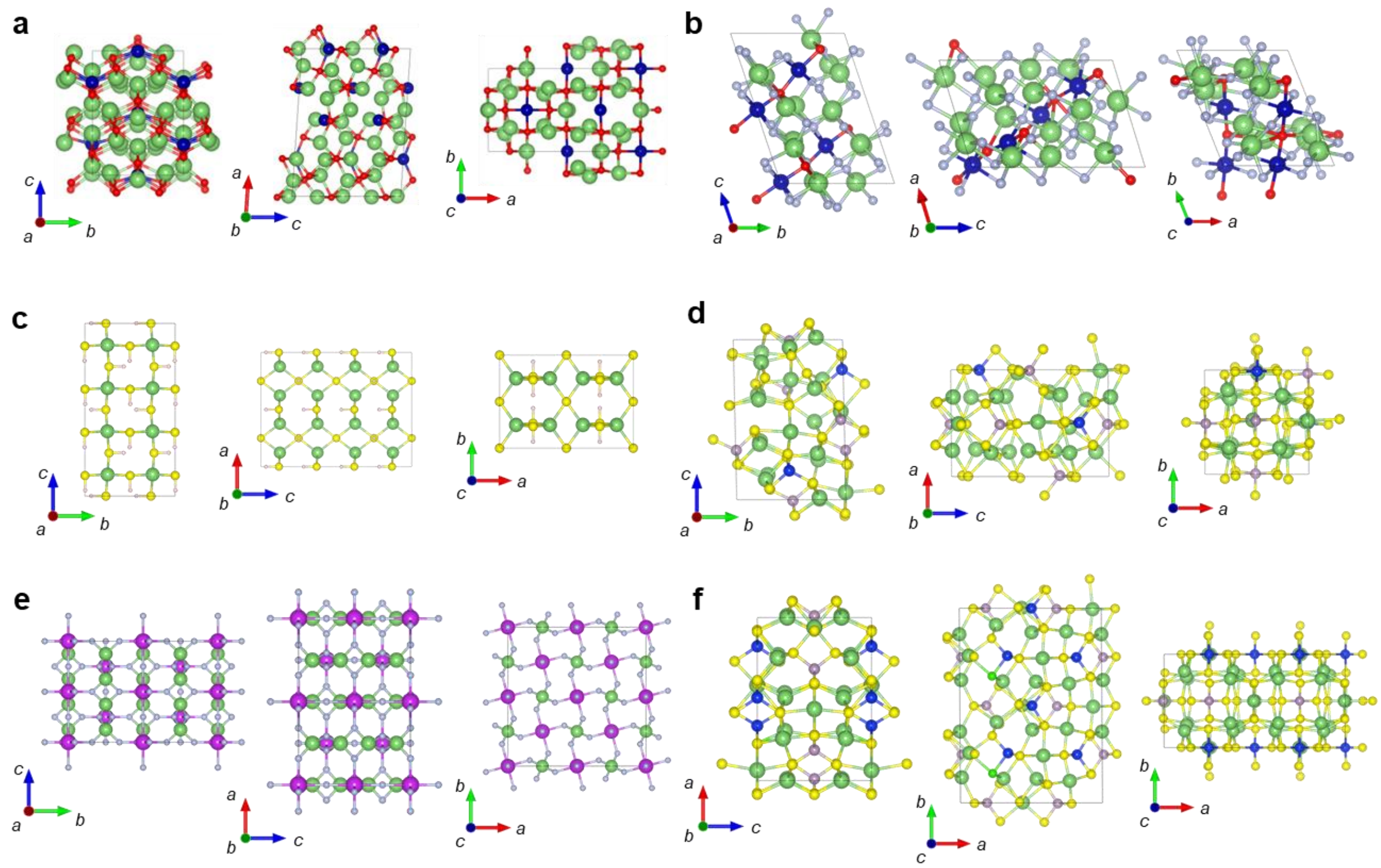

Fig. S15. Visualization of optimized structures. **a** Tetragonal Li_6CoO_4 (mp-18925) with a space group of $P4_2/nmc$. The optimized crystal constants: *a* (12.41498 Å), *b* (6.71336 Å), *c* (9.14606 Å), *α* (90.0004°), *β* (86.1862°), *γ* (89.9991°). **b** Triclinic Li_4CoOF_5 (mp-1278660) with a space group of P1. The optimized crystal constants: *a* (6.67760 Å), *b* (7.54104 Å), *c* (10.13662 Å), *α* (103.5880°), *β* (101.4079°), *γ* (108.0437°). **c** Orthorhombic LiHS (mp-644419) with a space group of Pmc2_1. The optimized crystal constants: *a* (11.96318 Å), *b* (8.26963 Å), *c* (15.79642 Å), *α* (90.0000°), *β* (90.0000°), *γ* (90.0000°). **d** Orthorhombic $Li_{10}SiP_2S_{12}$ (mp-720509) with a space group of Pna2_1. The optimized crystal constants: *a* (8.73534 Å), *b* (8.78623 Å), *c* (12.88887 Å), *α* (91.2636°), *β* (90.5096°), *γ* (90.2060°). **e** Tetragonal Li_2BiF_5 (mp-760419) with a space group of I4/m. The optimized crystal constants: *a* (13.81347 Å), *b* (13.81346 Å), *c* (9.32669 Å), *α* (90.0000°), *β* (90.0000°), *γ* (90.0000°). **f** Monoclinic $Li_{20}Si_3P_3S_{23}Cl$ (mp-1097035) with a space group of P1m1. The optimized crystal constants: *a* (17.43889 Å), *b* (8.95785 Å), *c* (12.71443 Å), *α* (90.0000°), *β* (90.4057°), *γ* (90.0000°).

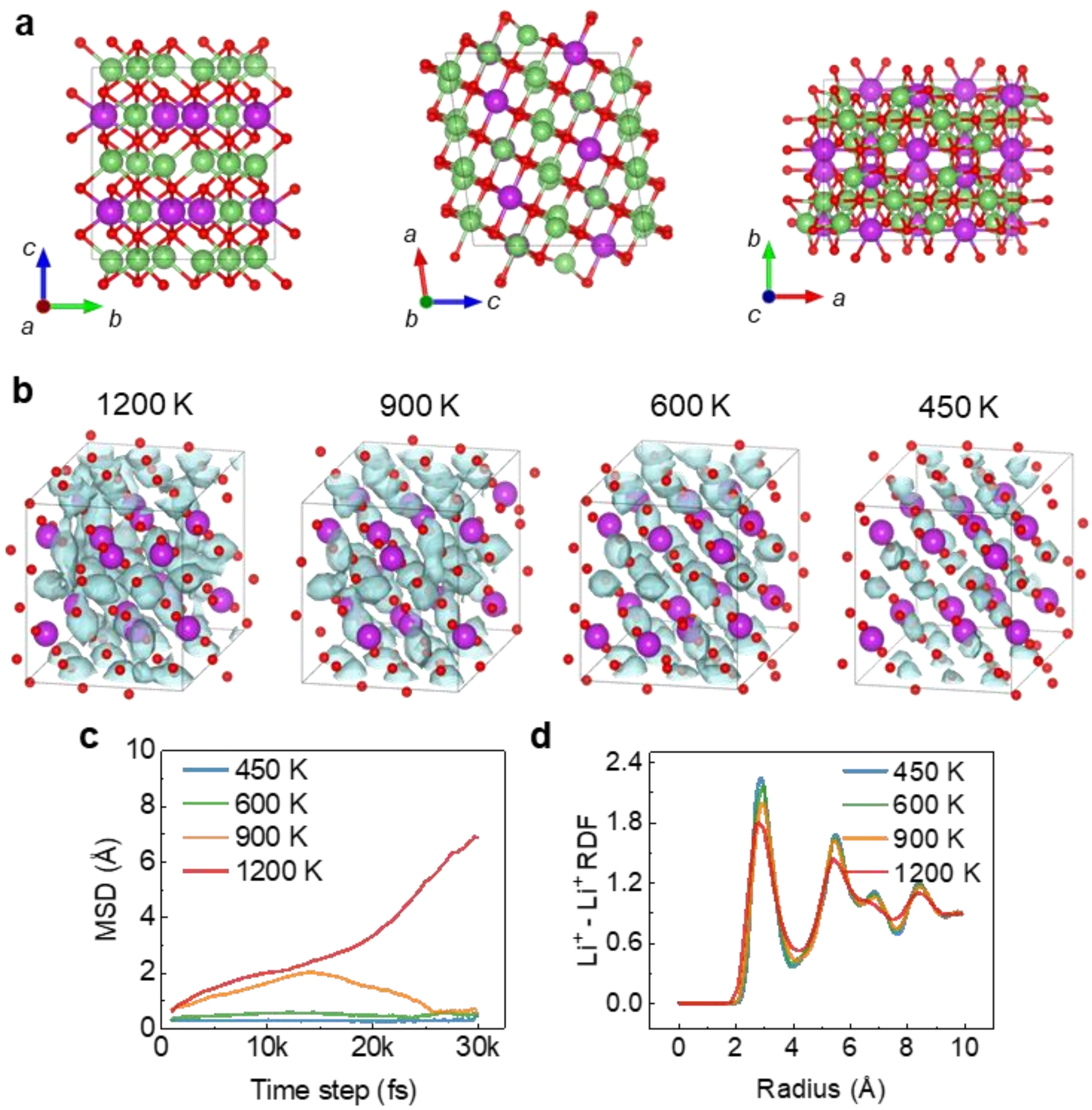

Fig. S16. Theoretical validation of Li_2BiO_3 (mp-769026). a Visualization of the optimized structure. The crystal system is monoclinic, and the space group is C12/c1. The optimized crystal constants: *a* (11.51547 Å), *b* (9.64757 Å), *c* (10.21760 Å), *α* (89.9999°), *β* (98.2851°), *γ* (90.0003°). **b** Three-dimensional migration path of Li^+. **c** Mean square displacement (MSD) over time at various temperatures (1200 K, 900 K, 600 K, and 450 K). **d** Plots of radial distribution function (RDF) of Li^+-Li^+.

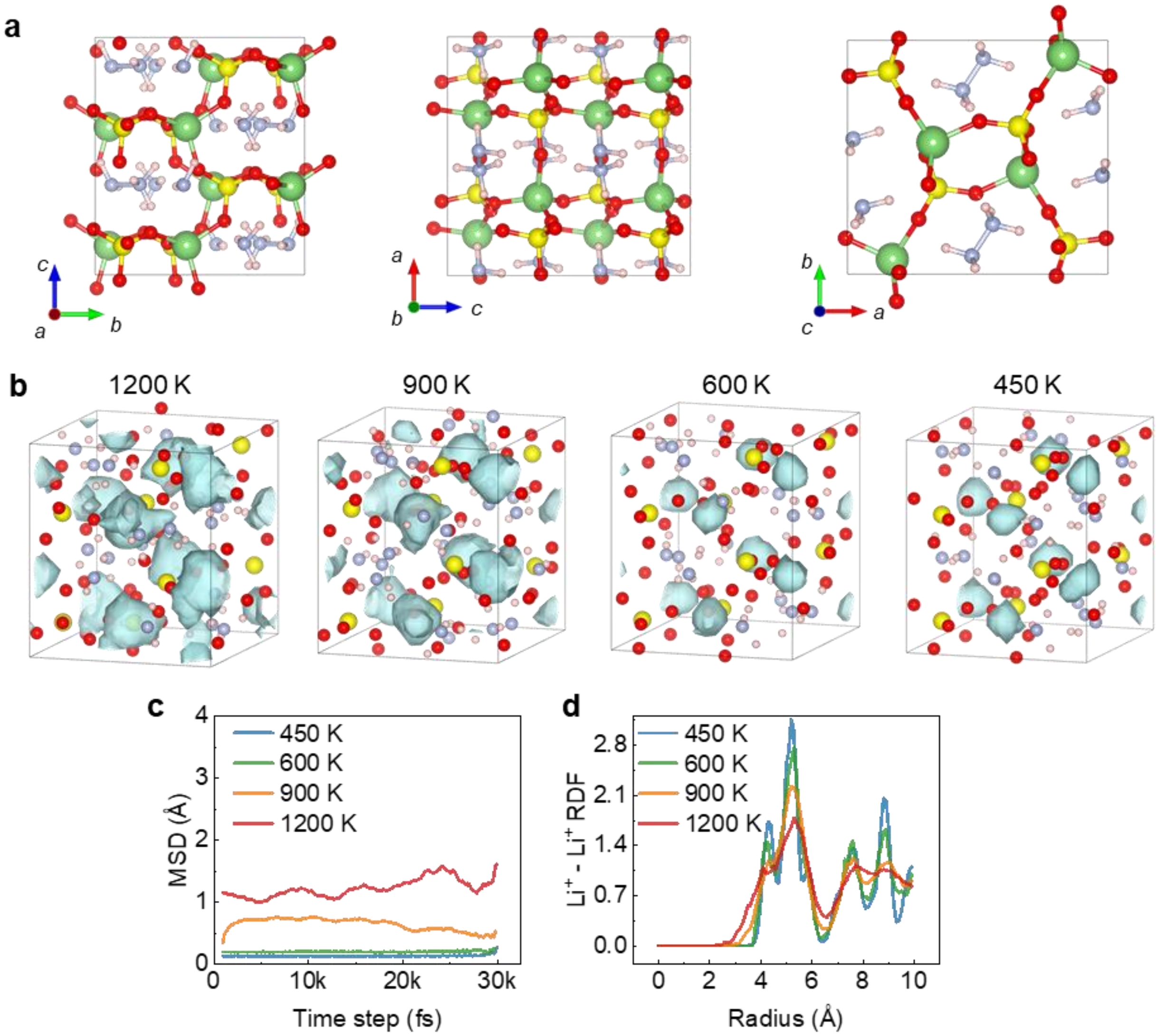

Fig. S17. Theoretical validation of $LiH_5S(NO_2)_2$ (mp-504426). a Visualization of the optimized structure. The crystal system is orthorhombic, and the space group is Pna2_1. The optimized crystal constants: *a* (9.88025 Å), *b* (8.86672 Å), *c* (10.04970 Å), *α* (90.0001°), *β* (90.0190°), *γ* (90.0000°). **b** Three-dimensional migration path of Li^+. **c** Mean square displacement (MSD) over time at various temperatures (1200 K, 900 K, 600 K, and 450 K). **d** Plots of radial distribution function (RDF) of Li^+-Li^+.

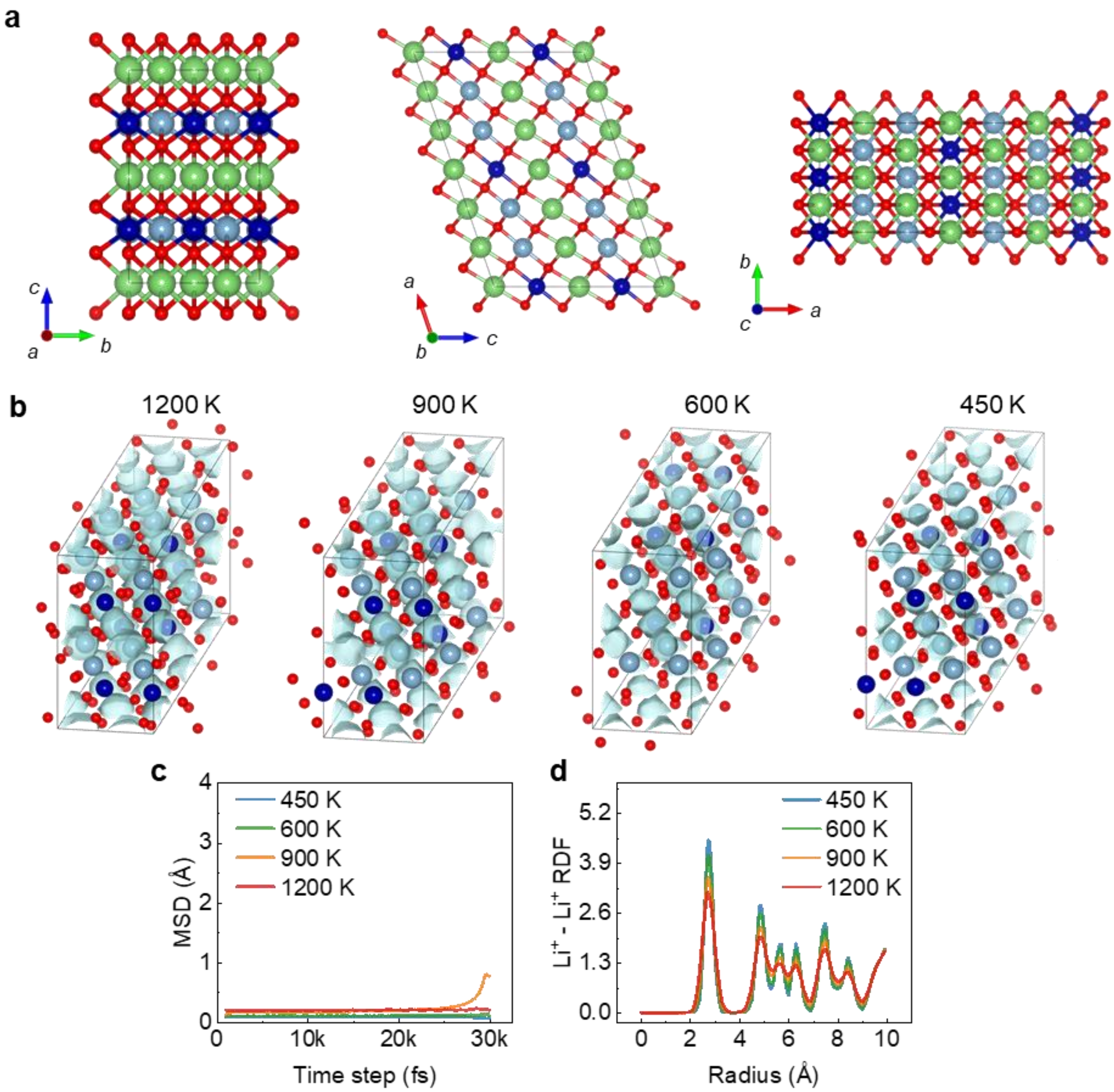

Fig. S18. Theoretical validation of $Li_3Al_2CoO_6$ (mp-1222601). a Visualization of the optimized structure. The crystal system is monoclinic, and the space group is C12/m1. The optimized crystal constants: *a* (14.47337 Å), *b* (5.72132 Å), *c* (9.90448 Å), *α* (90.0000°), *β* (108.9344°), *γ* (90.0000°). **b** Three-dimensional migration path of Li^+. **c** Mean square displacement (MSD) over time at various temperatures (1200 K, 900 K, 600 K, and 450 K). **d** Plots of radial distribution function (RDF) of Li^+-Li^+.

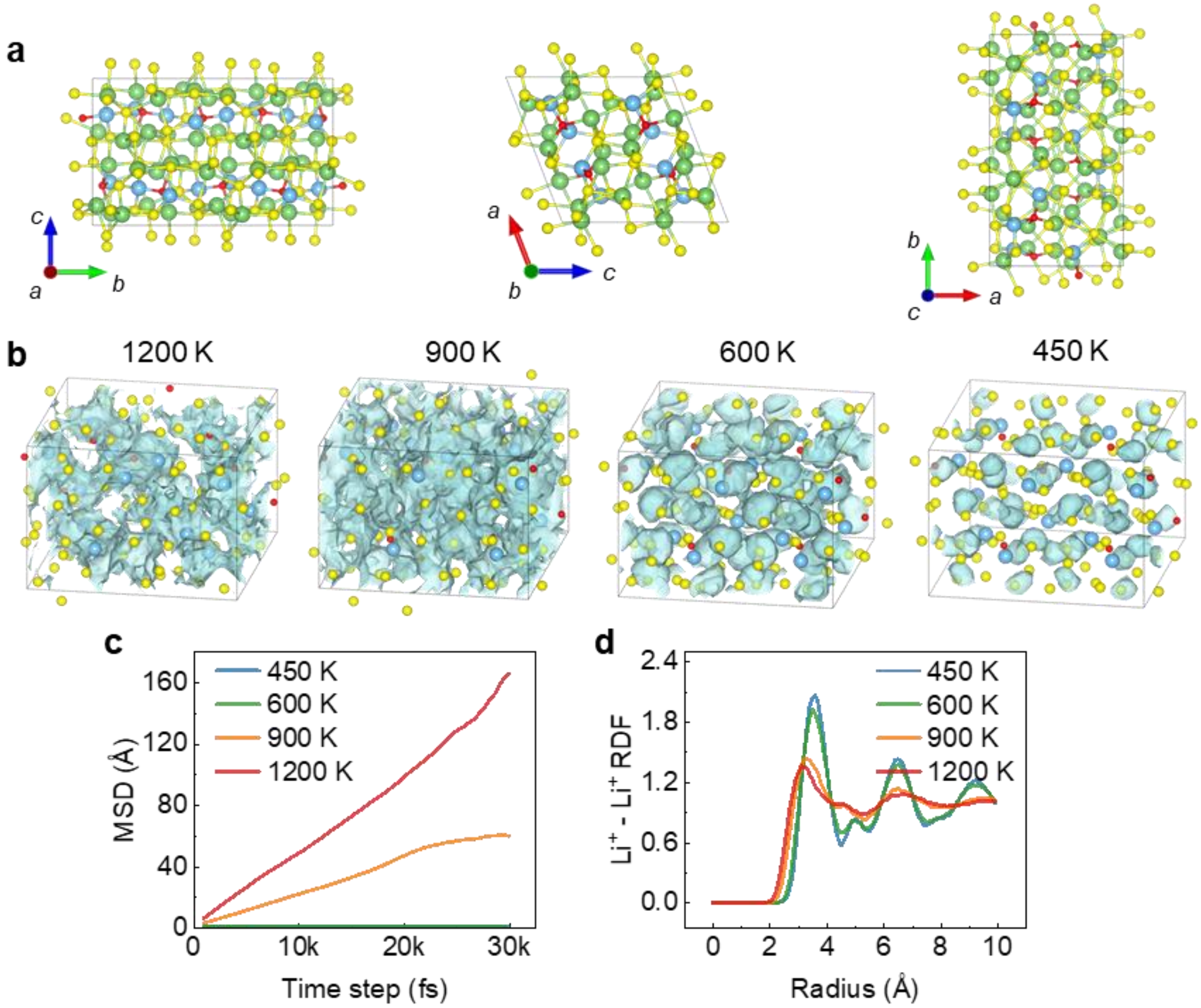

Fig. S19. Theoretical validation of $Li_6Ti_2S_6O$ (mp-770212). a Visualization of the optimized structure. The crystal system is monoclinic, and the space group is $P12_1/c1$. The optimized crystal constants: *a* (11.18259 Å), *b* (18.08815 Å), *c* (11.85592 Å), *α* (89.9905°), *β* (111.1274°), *γ* (90.0038°). **b** Three-dimensional migration path of Li^+. **c** Mean square displacement (MSD) over time at various temperatures (1200 K, 900 K, 600 K, and 450 K). **d** Plots of radial distribution function (RDF) of Li^+-Li^+.

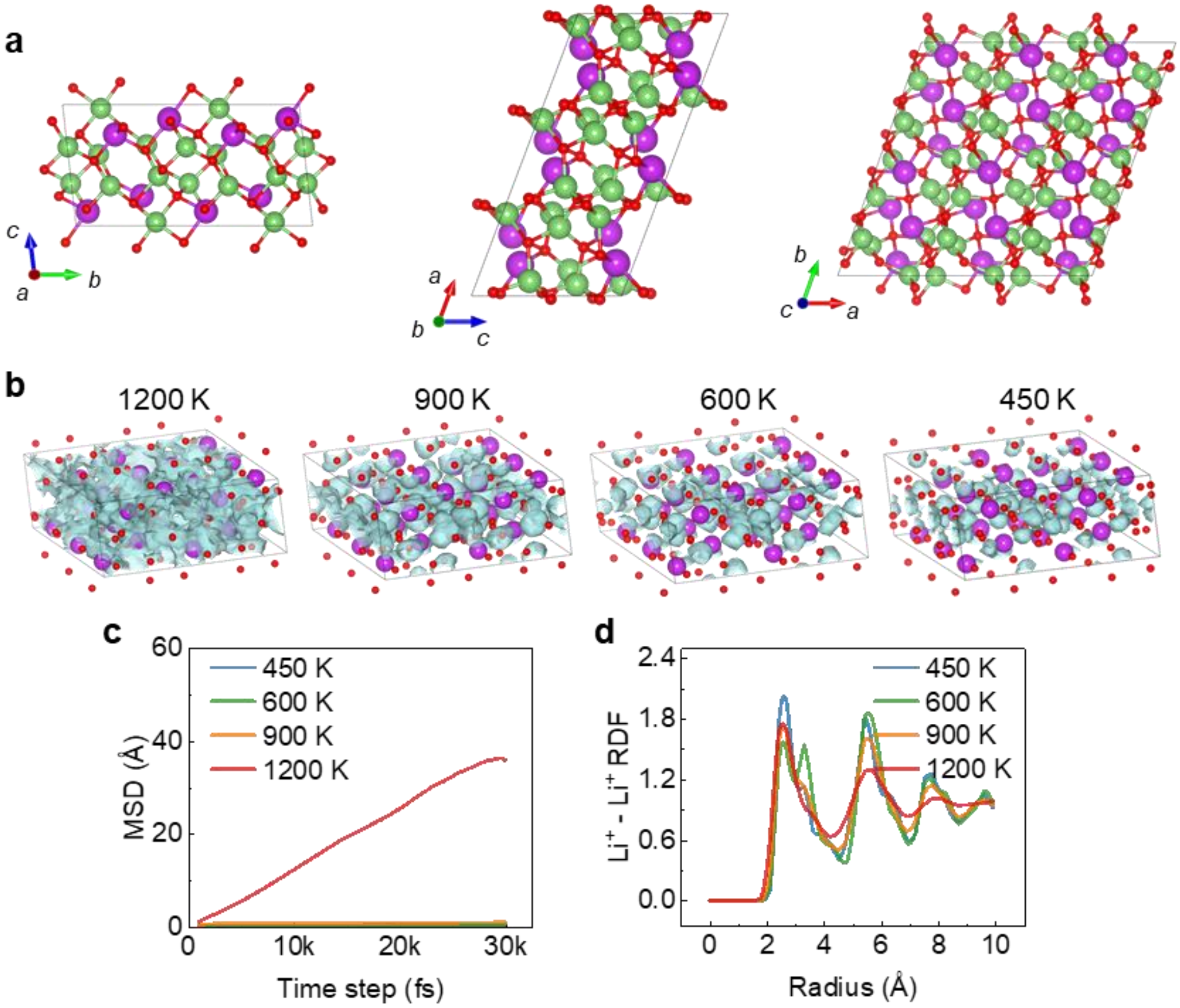

Fig. S20. Theoretical validation of $Li_4Bi_2O_5$ (mp-756718). a Visualization of the optimized structure. The crystal system is Triclinic, and the space group is P1. The optimized crystal constants: *a* (16.48151 Å), *b* (15.09145 Å), *c* (7.76493 Å), *α* (89.4803°), *β* (70.3269°), *γ* (71.2397°). **b** Three-dimensional migration path of Li^+. **c** Mean square displacement (MSD) over time at various temperatures (1200 K, 900 K, 600 K, and 450 K). **d** Plots of radial distribution function (RDF) of Li^+-Li^+.

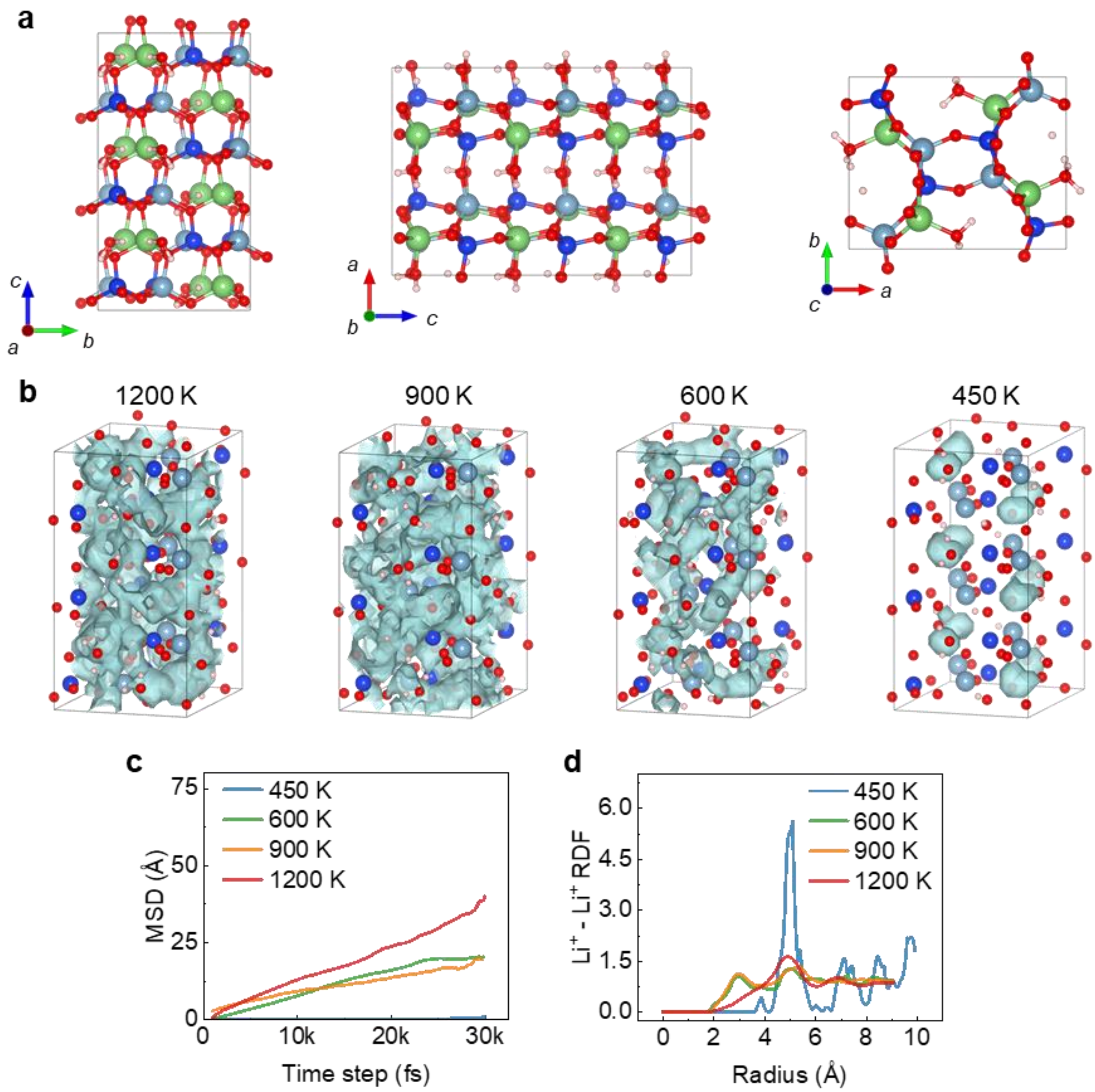

Fig. S21. Theoretical validation of $LiAlSiH_2O_5$ (mp-697020). **a** Visualization of the optimized structure. The crystal system is orthorhombic, and the space group is Pna2_1. The optimized crystal constants: *a* (10.27820 Å), *b* (8.16271 Å), *c* (14.79214 Å), *α* (90.0000°), *β* (90.0000°), *γ* (90.0000°). **b** Three-dimensional migration path of Li^+. **c** Mean square displacement (MSD) over time at various temperatures (1200 K, 900 K, 600 K, and 450 K). **d** Plots of radial distribution function (RDF) of Li^+-Li^+.

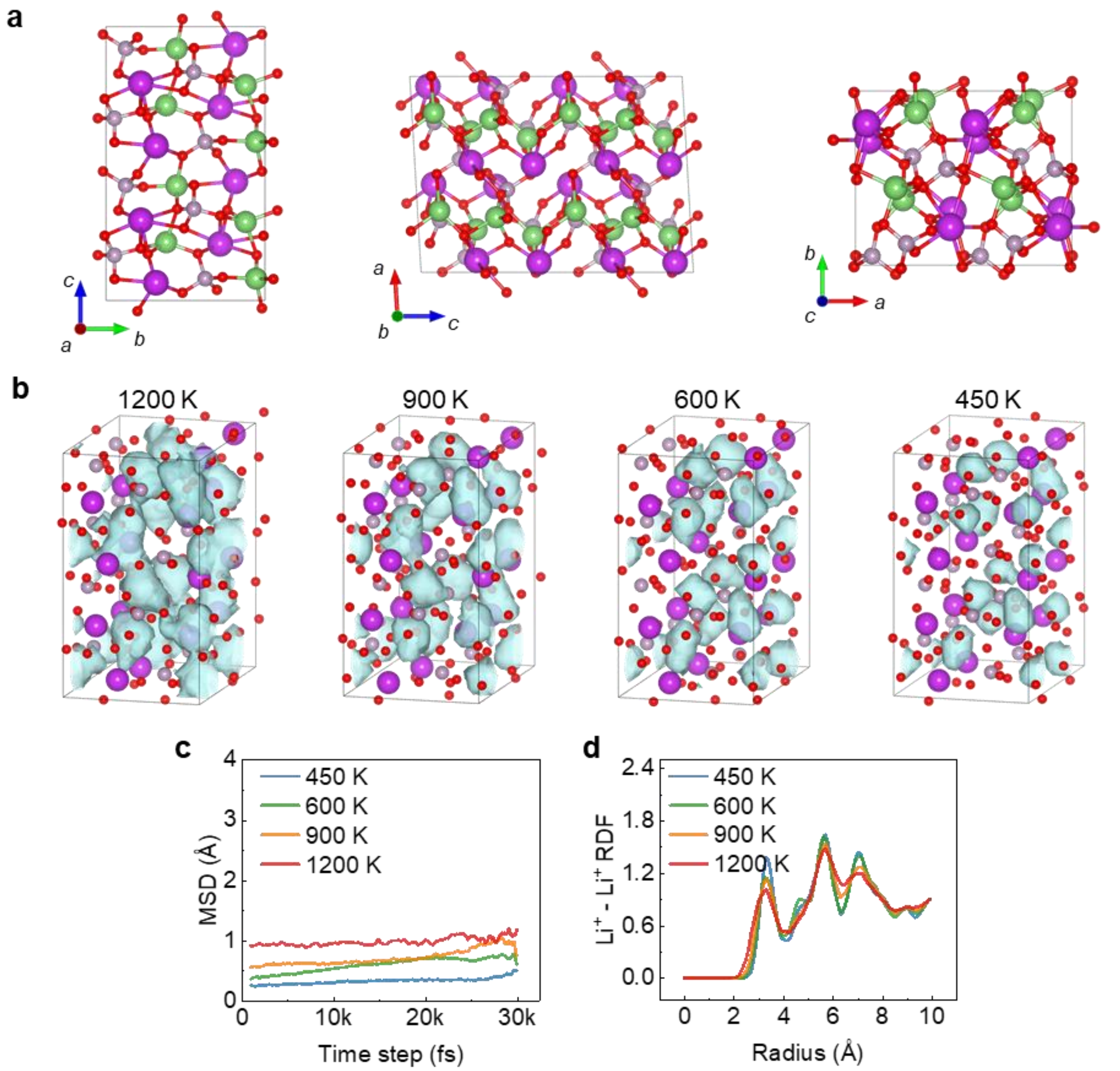

Fig. S22. Theoretical validation of $Li_2Bi_2P_2O_9$ (mp-759784). a Visualization of the optimized structure. The crystal system is monoclinic, and the space group is P12_11. The optimized crystal constants: *a* (11.18023 Å), *b* (8.99680 Å), *c* (15.54191 Å), *α* (89.9994°), *β* (93.3111°), *γ* (90.0000°). **b** Three-dimensional migration path of Li^+. **c** Mean square displacement (MSD) over time at various temperatures (1200 K, 900 K, 600 K, and 450 K). **d** Plots of radial distribution function (RDF) of Li^+-Li^+.

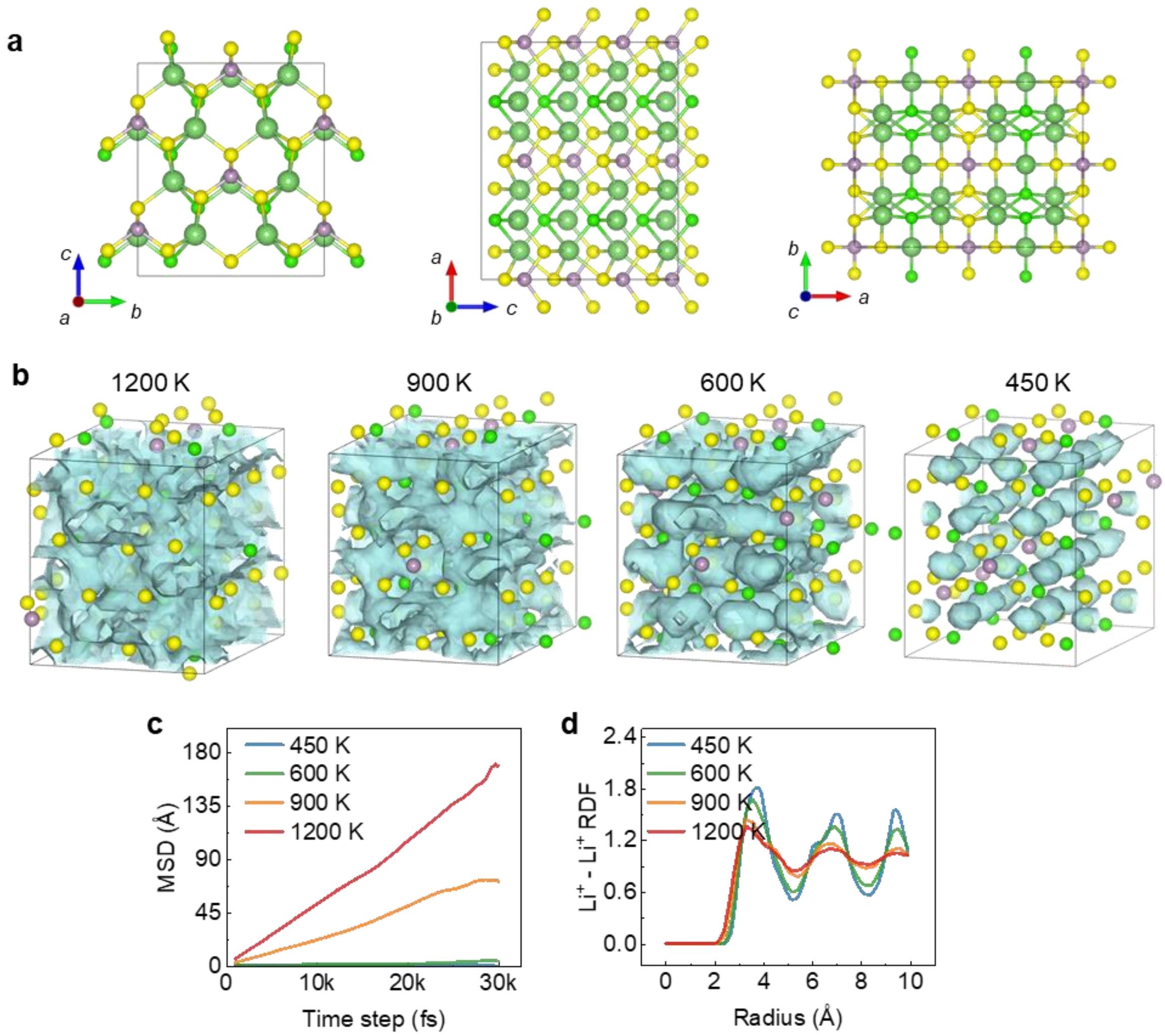

Fig. S23. Theoretical validation of $Li_5P(S_2Cl)_2$ (mp-1040450). a Visualization of the optimized structure. The crystal system is orthorhombic, and the space group is Amm2. The optimized crystal constants. *a* (14.45234 Å), *b* (10.51306 Å), *c* (12.01031 Å), *α* (90.0000°), *β* (90.0000°), *γ* (90.0000°). **b** Three-dimensional migration path of Li^+. **c** Mean square displacement (MSD) over time at various temperatures (1200 K, 900 K, 600 K, and 450 K). **d** Plots of radial distribution function (RDF) of Li^+-Li^+.

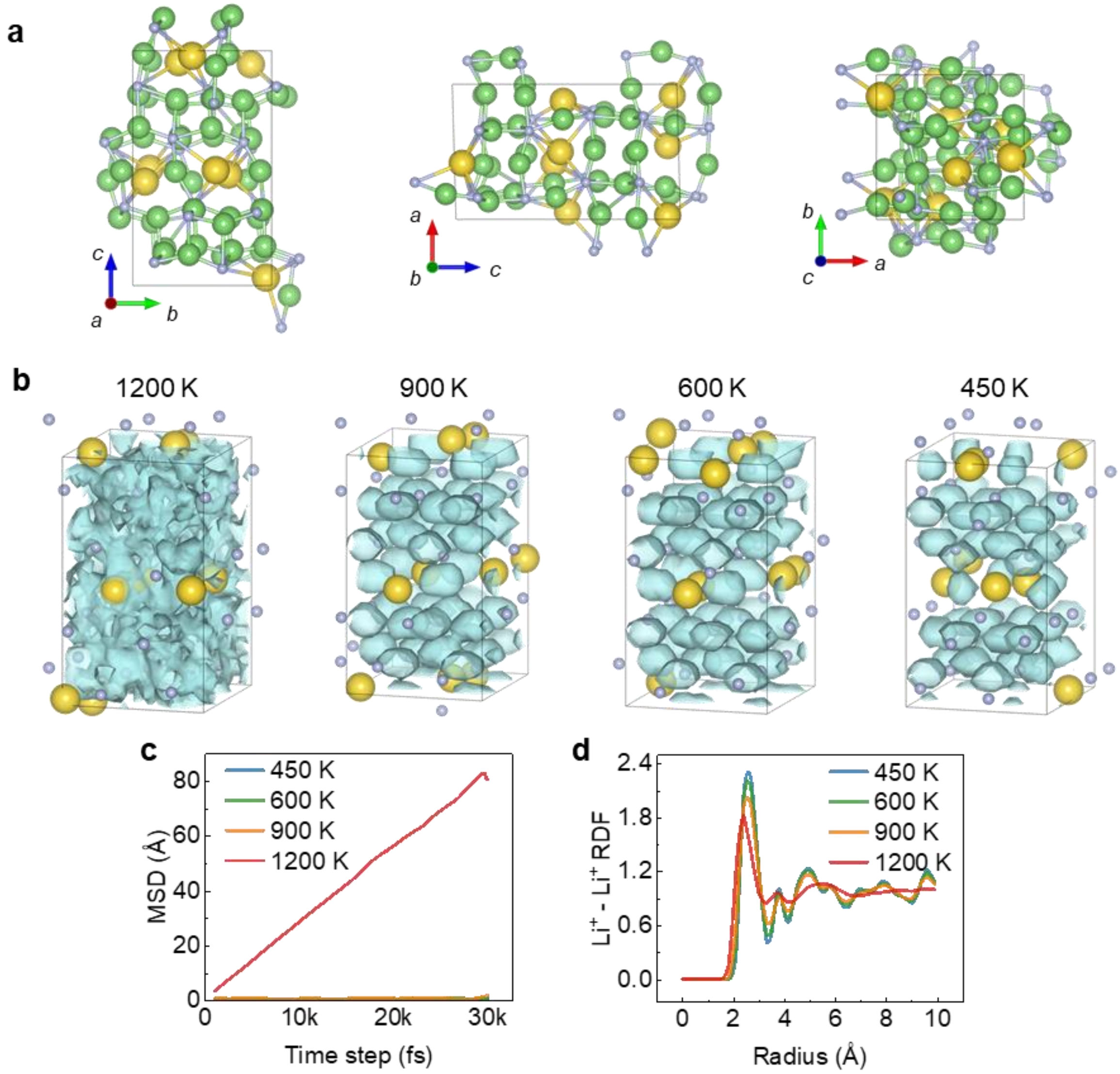

Fig. S24. Theoretical validation of $NaLi_5N_2$ (mp-1186148). **a** Visualization of the optimized structure. The crystal system is monoclinic, and the space group is P1m1. The optimized crystal constants. *a* (7.64756 Å), *b* (7.50464 Å), *c* (12.69633 Å), *α* (90.0000°), *β* (91.5913°), *γ* (90.0000°). **b** Three-dimensional migration path of Li^+. **c** Mean square displacement (MSD) over time at various temperatures (1200 K, 900 K, 600 K, and 450 K). **d** Plots of radial distribution function (RDF) of Li^+-Li^+.

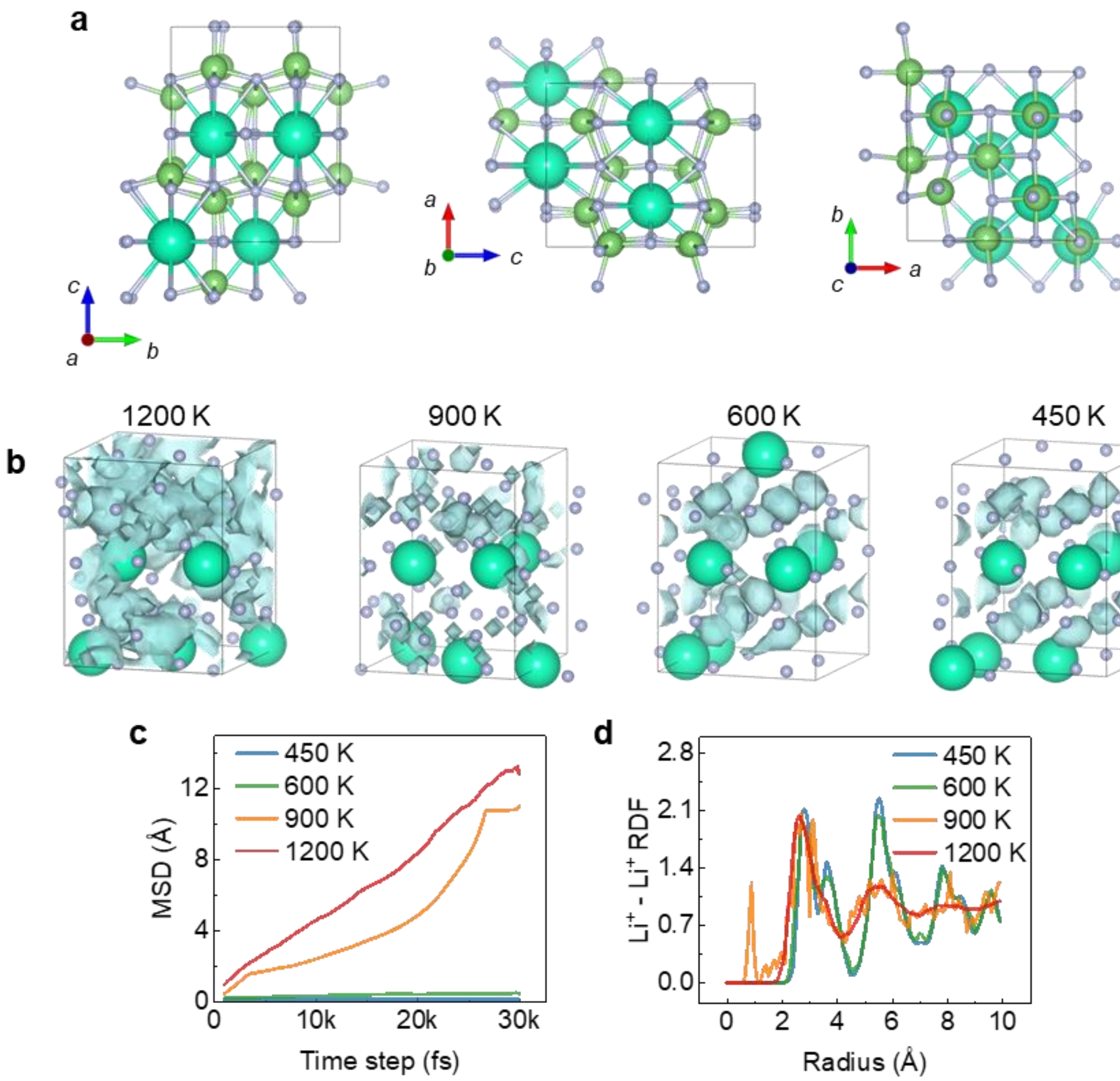

Fig. S25. Theoretical validation of $CsLi_2F_3$ (mp-606604). a Visualization of the optimized structure. The crystal system is tetragonal, and the space group is I4/mmm. The optimized crystal constants. *a* (7.72957 Å), *b* (7.72957 Å), *c* (9.82570 Å), *α* (90.0000°), *β* (90.0000°), *γ* (90.2177°). **b** Three-dimensional migration path of Li^+. **c** Mean square displacement (MSD) over time at various temperatures (1200 K, 900 K, 600 K, and 450 K). **d** Plots of radial distribution function (RDF) of Li^+-Li^+.

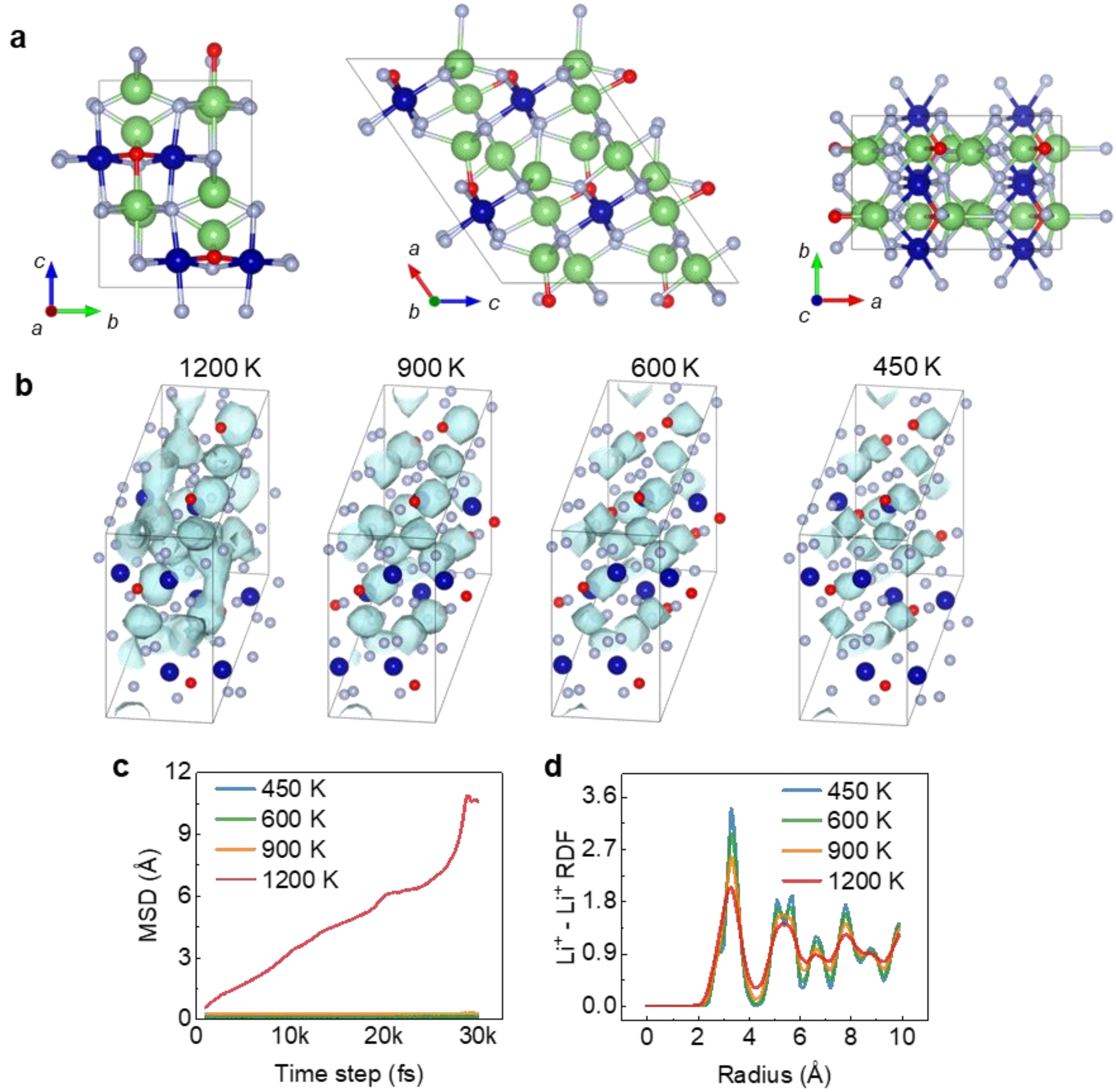

Fig. S26. Theoretical validation of $Li_4Co_2OF_7$ (mp-1311449). a Visualization of the optimized structure. The crystal system is monoclinic, and the space group is P1c1. The optimized crystal constants. *a* (11.30609 Å), *b* (5.92700 Å), *c* (9.79956 Å), *α* (90.0000°), *β* (124.7935°), *γ* (90.0000°). **b** Three-dimensional migration path of Li^+. **c** Mean square displacement (MSD) over time at various temperatures (1200 K, 900 K, 600 K, and 450 K). **d** Plots of radial distribution function (RDF) of Li^+-Li^+.

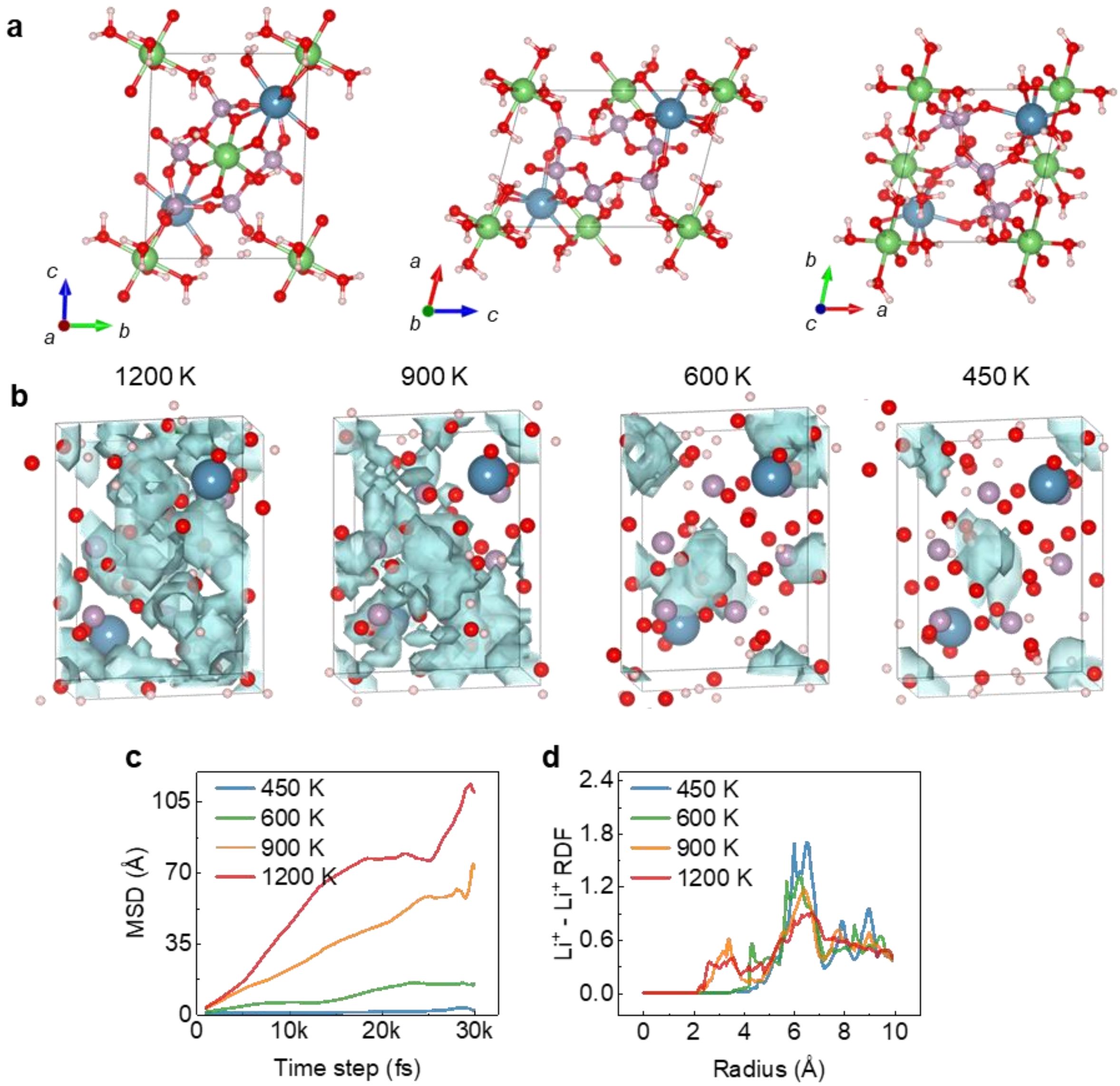

Fig. S27. Theoretical validation of $LiCaP_3H_8O_{13}$ (mp-721067). a Visualization of the optimized structure. The crystal system is triclinic, and the space group is P1. The optimized crystal constants. *a* (7.18618 Å), *b* (7.58794 Å), *c* (10.02666 Å), *α* (84.8181°), *β* (74.7666°), *γ* (77.3288°). **b** Three-dimensional migration path of Li^+. **c** Mean square displacement (MSD) over time at various temperatures (1200 K, 900 K, 600 K, and 450 K). **d** Plots of radial distribution function (RDF) of Li^+-Li^+.

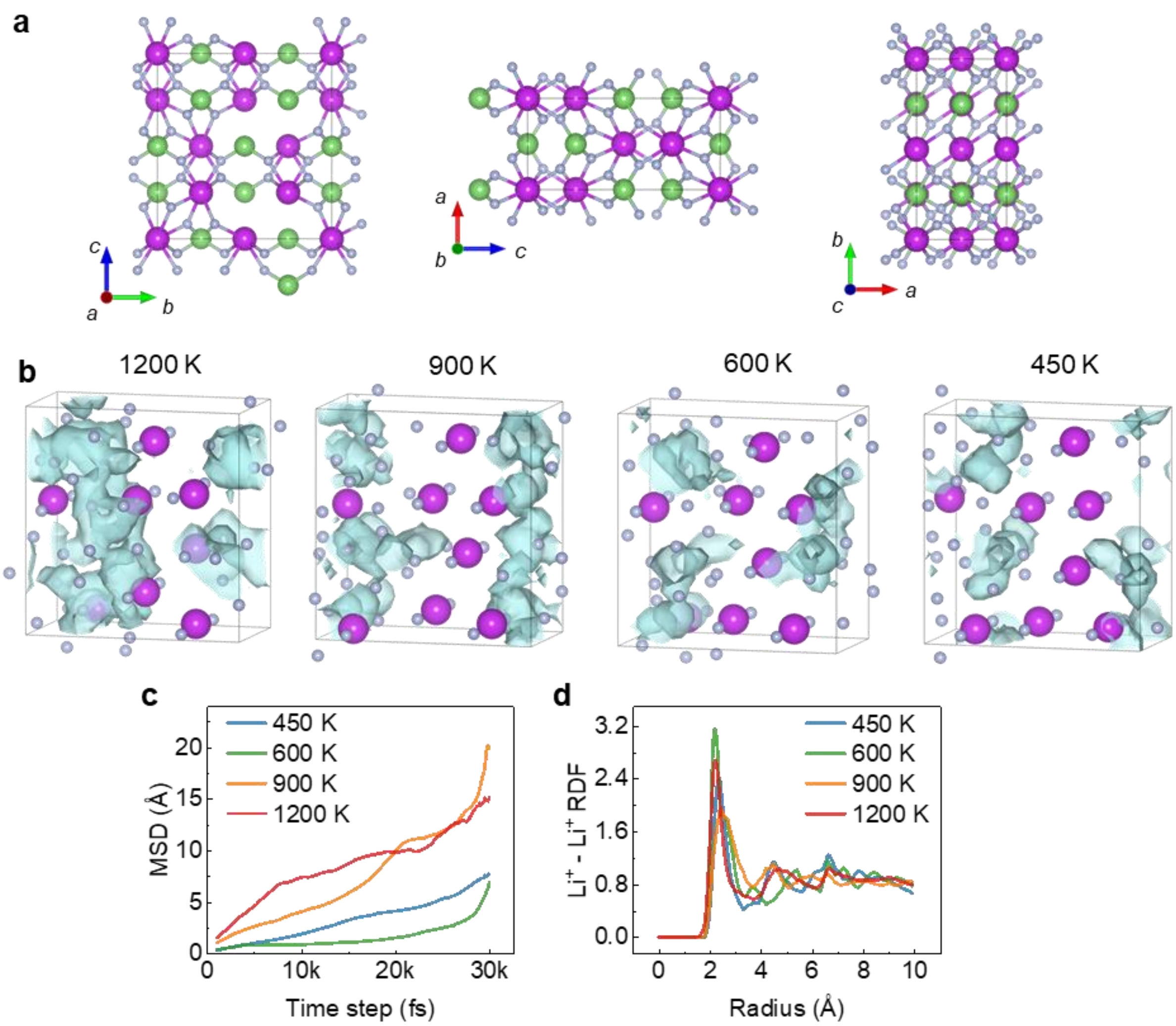

Fig. S28. Theoretical validation of $LiBiF_4$ (mp-28567). **a** Visualization of the optimized structure. The crystal system is tetragonal, and the space group is $I4_1/a$. The optimized crystal constants. *a* (4.44147 Å), *b* (10.81450 Å), *c* (11.11084 Å), *α* (88.8853°), *β* (90.2506°), *γ* (91.4444°). **b** Three-dimensional migration path of Li^+. **c** Mean square displacement (MSD) over time at various temperatures (1200 K, 900 K, 600 K, and 450 K). **d** Plots of radial distribution function (RDF) of Li^+-Li^+.

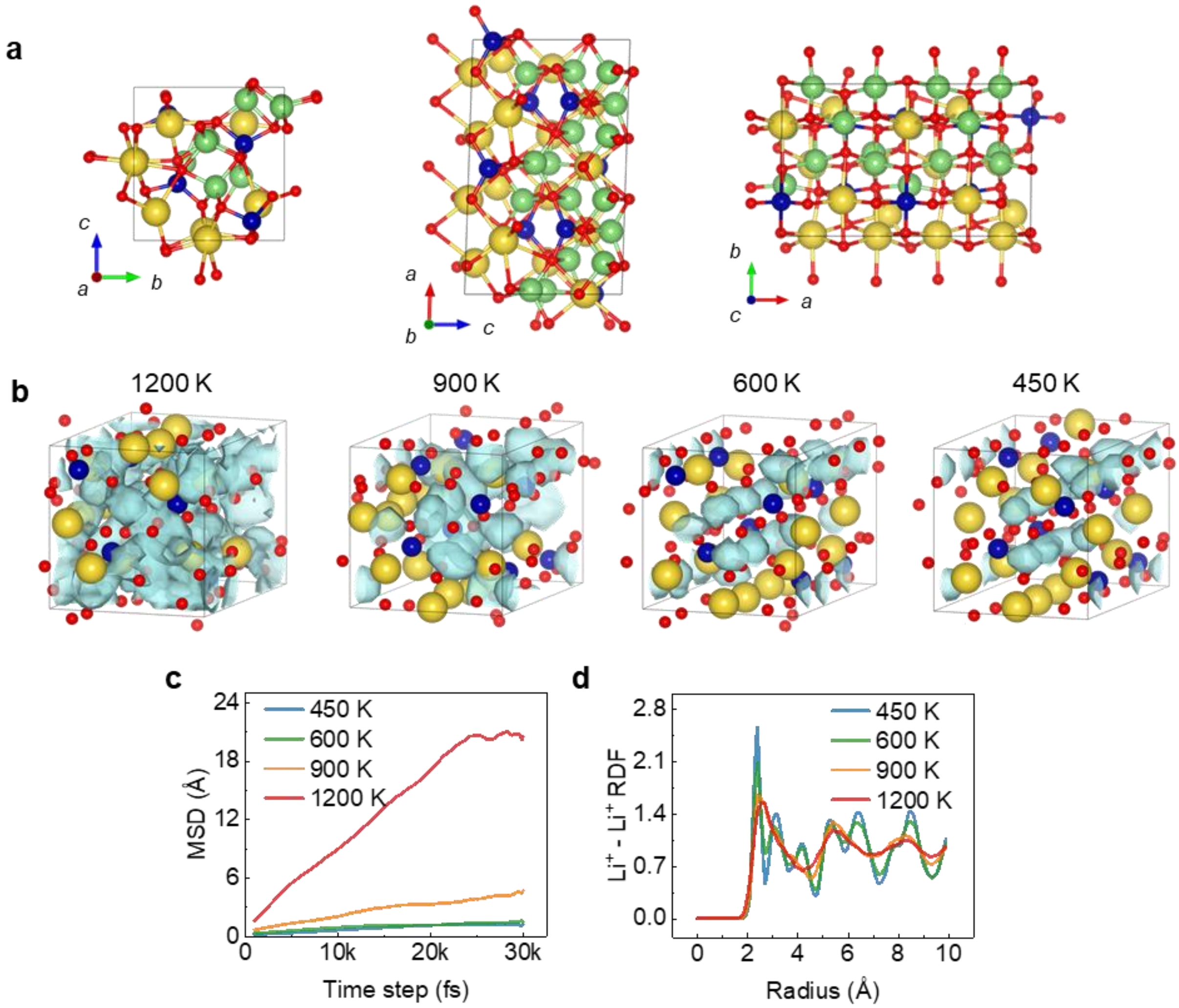

Fig. S29. Theoretical validation of $Na_2Li_2CoO_4$ (mp-861533). a Visualization of the optimized structure. The crystal system is triclinic, and the space group is P1. The optimized crystal constants. *a* (12.99721 Å), *b* (7.91128 Å), *c* (8.04270 Å), *α* (89.9411°), *β* (88.1327°), *γ* (89.3375°). **b** Three-dimensional migration path of Li^+. **c** Mean square displacement (MSD) over time at various temperatures (1200 K, 900 K, 600 K, and 450 K). **d** Plots of radial distribution function (RDF) of Li^+-Li^+.

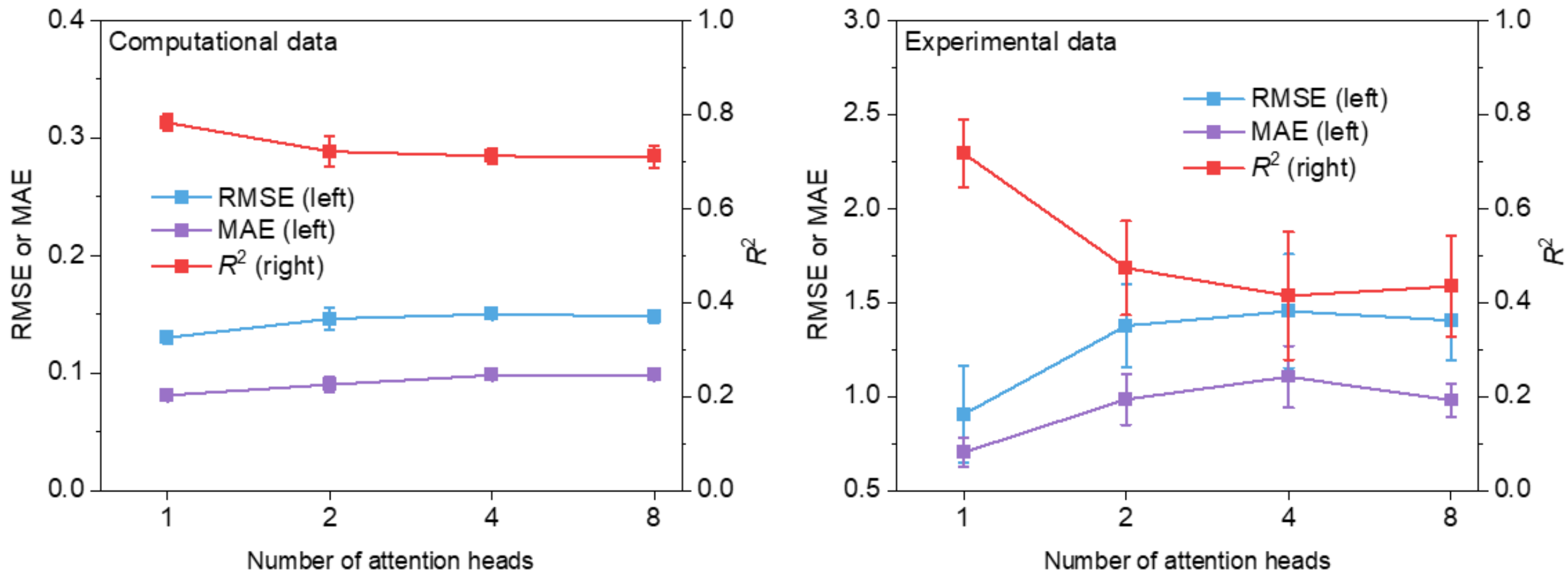

Fig. S30. Effect of the number of attention heads. Additional experiments with different numbers of attention heads (2, 4, and 8) were conducted. Given that IonNet-TL, which integrates three descriptors, has been confirmed to exhibit optimal predictive performance, all models presented here were trained using these three descriptors.

Table S1. Experimental data around room temperature. Chemical composition, temperature (*T*: °C), and ionic conductivity (*σ*: (S cm^{-1})) of 398 experimental data points are listed. The range of *σ* is 1.12E-10~2.50E-02 S cm^{-1}. The *T* is rounded to integer.

Composition	*T*	*σ*	Composition	*T*	*σ*	Composition	*T*	*σ*
$((Li_2S)_{0.6}(SiS_2)_{0.4})_{95}(Li_4SiO_4)_5$	25	5.88E-03	$Li_{1.6}Al_{0.6}Ge_{1.0}Ti_{0.4}(PO_4)_3$	25	5.00E-05	$Li_{4.4}Ga_{0.4}Sn_{0.6}S_4$	30	3.20E-06
$(AgI)_{0.5}(LiPO_3)_{0.5}$	30	4.10E-08	$Li_{1.6}Al_{0.6}Ge_{1.4}(PO_4)_3$	25	2.60E-04	$Li_{4.8}Si_{0.2}Al_{0.8}S_4$	25	1.07E-06
$(La_{0.5}Li_{0.5})_{0.75}(La_{0.5}Na_{0.5})_{0.25}TiO_3$	27	1.58E-04	$Li_{1.6}Cr_{0.6}Ti_{1.4}(PO_4)_3$	25	1.47E-04	$Li_4Al_{0.33}Si_{0.17}Ge_{0.17}P_{0.33}O_4$	25	1.61E-06
$(La_{0.5}Li_{0.5})TiO_3$	25	1.00E-03	$Li_{1.6}Ga_{0.6}Ti_{1.4}(PO_4)_3$	25	1.30E-05	$Li_4Al_{0.33}Si_{0.33}P_{0.33}O_4$	25	3.25E-06
$(Li_{0.5}La_{0.5}TiO_3)_{94}(SiO_2)_6$	15	6.30E-06	$Li_{1.6}Ti_{1.7}Mg_{0.3}P_3O_{12}$	25	2.80E-06	$Li_4B_7O_{12}Cl$	25	1.00E-07
$(Li_{0.5}La_{0.5}TiO_3)_{95}(SiO_2)_5$	15	1.25E-05	$Li_{1.7}Al_{0.7}Ge_{1.3}(PO_4)_3$	25	1.50E-04	$Li_4BN_3H_{10}$	25	2.00E-04
$(Li_{0.5}La_{0.5}TiO_3)_{97}(SiO_2)_3$	15	1.00E-05	$Li_{1.7}Al_{0.7}Ti_{1.3}(PO_4)_3$	25	3.70E-05	Li_4GeS_4	25	2.51E-07
$(Li_2O)_{16.6}(SiO_2)_{83.4}$	25	1.30E-09	$Li_{1.7}Fe_{0.7}Ge_{1.3}(PO_4)_3$	25	2.37E-05	$Li_4Nd_3TeSbO_{12}$	25	8.70E-09
$(Li_2O)_{50}((SeO_2)_{0.5}(B_2O_3)_{0.5})_{50}$	27	7.54E-07	$Li_{1.7}Ga_{0.7}Ge_{1.3}(PO_4)_3$	25	2.43E-05	$Li_4P_2S_6$	25	1.29E-07
$(Li_2O)_{50}(Al_2O_3)_2(P_2O_5)_{48}$	25	3.16E-09	$Li_{1.8}Al_{0.8}Ge_{0.8}Ti_{0.4}(PO_4)_3$	25	7.00E-05	Li_4PS_4I	25	6.26E-05
$(Li_2O)_{50}(Al_2O_3)_3(P_2O_5)_{47}$	25	5.75E-09	$Li_{1.5}Fe_{0.5}Ti_{1.5}(PO_4)_3$	25	1.75E-04	Li_4SiO_4	25	2.00E-10
$(Li_2O)_{50}(Al_2O_3)_4(P_2O_5)_{46}$	25	9.12E-09	$Li_{1.5}Ga_{0.5}Ge_{1.5}(PO_4)_3$	25	1.17E-04	Li_4SiS_4	25	4.93E-08
$(Li_2O)_{50}(Al_2O_3)_5(P_2O_5)_{45}$	25	1.15E-08	$Li_{1.5}Hf_{1.5}Al_{0.5}P_3O_{12}$	25	1.10E-04	Li_4SnS_4	25	7.00E-05
$(Li_2O)_{50}(LiF)_{20}(P_2O_5)_{30}$	25	4.40E-07	$Li_{1.5}In_{0.5}Ge_{1.5}(PO_4)_3$	25	5.75E-06	$Li_{5.4}La_3Nb_{1.8}Y_{0.75}O_{12}$	25	2.51E-05
$(Li_2O)_{50}(P_2O_5)_{50}$	25	1.32E-09	$Li_{1.5}In_{0.5}Ti_{1.5}(PO_4)_3$	25	2.04E-04	$Li_{5.5}La_{2.75}K_{0.25}Nb_2O_{12}$	25	1.06E-05
$(Li_2S)(LaOI)$	30	2.75E-07	$Li_{1.5}La_{0.5}Ti_{1.5}(PO_4)_3$	25	2.82E-04	$Li_{5.5}La_3Nb_{1.75}In_{0.25}O_{12}$	25	2.40E-05
$(Li_2S)_{0.3}((Ga_2S_3)_{0.1}(GeS_2)_{0.9})_{0.7}$	25	1.89E-06	$Li_{1.5}Sc_{0.5}Ge_{1.5}(PO_4)_3$	25	2.51E-05	Li_5AlS_4	25	1.26E-08
$(Li_2S)_{0.3}((P_2S_5)_{0.4}(Sb_2S_3)_{0.6})_{0.7}$	31	2.98E-08	$Li_{1.5}Y_{0.5}Ti_{1.5}(PO_4)_3$	25	5.44E-05	$Li_5La_3Bi_2O_{12}$	25	2.00E-05
$(Li_2S)_{0.4}((Ga_2S_3)_{0.1}(GeS_2)_{0.9})_{0.6}$	21	2.87E-06	$Li_{1.6}Al_{0.6}Ge_{0.8}(PO_4)_3$	25	7.00E-04	$Li_5La_3Nb_2O_{12}$	15	8.00E-06
$(Li_2S)_{0.4}((P_2S_5)_{0.4}(Sb_2S_3)_{0.6})_{0.6}$	25	1.60E-07	$Li_{1.6}Al_{0.6}Ge_{0.8}Ti_{0.6}(PO_4)_3$	25	1.10E-04	$Li_5La_3Sb_2O_{12}$	25	2.06E-06
$(Li_2S)_{0.5}((Ga_2S_3)_{0.1}(GeS_2)_{0.9})_{0.5}$	28	6.70E-05	$Li_{1.4}Sc_{0.4}Ti_{1.6}(PO_4)_3$	25	5.15E-04	$Li_5La_3Ta_2O_{12}$	25	2.66E-06
$(Li_2S)_{0.5}((P_2S_5)_{0.4}(Sb_2S_3)_{0.6})_{0.5}$	34	2.26E-07	$Li_{1.4}Ti_{1.6}In_{0.4}P_3O_{12}$	25	1.90E-04	$Li_5Nd_3Sb_2O_{12}$	25	5.58E-07
$(Li_2S)_{0.7}(P_2S_5)_{0.3}$	25	3.20E-03	$Li_{1.4}Y_{0.4}Hf_{1.6}(PO_4)_3$	25	2.09E-05	Li_5NI_2	25	1.50E-07
$(Li_2S)_{0.75}(P_2S_5)_{0.25}$	31	3.93E-04	$Li_{1.4}Y_{0.4}Ti_{1.6}(PO_4)_3$	25	6.10E-04	$Li_5PS_4(BH_4)_2$	25	7.96E-03
$(Li_2S)_{0.8}(P_2S_5)_{0.2}$	25	1.12E-03	$Li_{1.50}Al_{0.50}Ge_{0.2}Ti_{1.3}(PO_4)_3$	25	7.37E-04	$Li_{6.02}P_{0.98}Si_{0.02}S_5Br$	25	8.74E-04
$(Li_2S)_{0.875}(P_2S_5)_{0.125}$	25	2.10E-04	$Li_{1.55}Al_{0.55}Ge_{0.2}Ti_{1.25}(PO_4)_3$	25	7.02E-05	$Li_{6.05}P_{0.95}Si_{0.05}S_5Br$	25	4.41E-04
$(Li_2S)_2(LaOI)_5$	30	6.92E-08	$Li_{1.5}Al_{0.5}Ge_{1.5}(PO_4)_3$	26	3.30E-03	$Li_{6.08}P_{0.92}Si_{0.08}S_5Br$	25	8.64E-04
$(Li_2S)_{50}(P_2S_5)_{17}(LiBH_4)_{33}$	25	1.43E-03	$Li_{1.5}Al_{0.5}Ti_{1.5}(PO_4)_3$	25	1.10E-05	$Li_{6.10}P_{0.90}Si_{0.10}S_5Br$	25	8.05E-04
$(Li_2S)_7(LaOI)_3$	30	2.88E-06	$Li_{1.5}Cr_{0.5}Hf_{1.5}(PO_4)_3$	25	1.18E-07	$Li_{6.12}P_{0.90}Si_{0.12}S_5Br$	25	9.00E-04
$(Li_2S)_{70}(P_2S_5)_{30}$	25	1.70E-02	$Li_{1.5}Cr_{0.5}Ti_{1.5}(PO_4)_3$	25	3.91E-04	$Li_{6.13}P_{0.87}Si_{0.13}S_5Br$	25	9.01E-04
$(Li_2S)_9(LaOI)$	30	8.13E-09	$Li_{1.4}Al_{0.4}Ge_{1.6}(PO_4)_3$	25	1.77E-04	$Li_{6.17}P_{0.83}Si_{0.17}S_5Br$	25	9.29E-04
$(LiCl)_{0.25}((Li_2O)_{0.6}(P_2O_5)_{0.4})_{0.75}$	24	2.45E-06	$Li_{1.4}Al_{0.4}Ti_{1.6}(PO_4)_3$	23	3.27E-03	$Li_{6.20}P_{0.80}Si_{0.20}S_5Br$	25	1.98E-03
$(LiCl)_{0.5}(P_2O_5)_{0.5}$	30	6.83E-08	$Li_{1.4}Cr_{0.4}Ti_{1.6}(PO_4)_3$	25	1.50E-04	$Li_{6.22}P_{0.78}Si_{0.22}S_5Br$	25	1.08E-03
$(LiI)_{0.3}((Li_2S)_{0.3}((Ga_2S_3)_{0.1}(GeS_2)_{0.9})_{0.7})_{0.7}$	25	2.91E-04	$Li_{1.4}Fe_{0.4}Ti_{1.6}(PO_4)_3$	25	3.86E-04	$Li_{6.25}Ba_{1.25}La_{1.75}Ta_2O_{12}$	26	7.80E-05
$(YPO_4)_{0.7}(Li_3PO_4)_{0.3}$	25	6.57E-09	$Li_{1.4}In_{0.4}Hf_{1.6}(PO_4)_3$	25	1.42E-04	$Li_{6.25}La_3Zr_2Ga_{0.25}O_{12}$	25	3.50E-04
$(YPO_4)_{0.8}(Li_3PO_4)_{0.2}$	25	1.41E-05	$Li_{1.4}In_{0.4}Ti_{1.6}(PO_4)_3$	25	3.00E-04	$Li_{6.25}P_{0.75}Si_{0.25}S_5Br$	25	1.50E-03
$(YPO_4)_{0.9}(Li_3PO_4)_{0.1}$	25	4.64E-07	$Li_{1.4}La_{0.4}Ti_{1.6}(PO_4)_3$	25	4.97E-04	$Li_{6.25}PS_{5.25}Cl_{0.75}$	25	1.40E-02
$La_{0.273}Li_{0.18}NbO_3$	25	3.82E-05	$Li_{1.4}Lu_{0.4}Hf_{1.6}(PO_4)_3$	25	2.06E-05	$Li_{6.2}La_3Zr_{1.2}Sb_{0.8}O_{12}$	25	4.50E-04
$La_{0.283}Li_{0.15}NbO_3$	25	3.85E-05	$Li_{1.4}S_{2.2}P_{0.6}$	25	6.97E-04	$Li_{6.2}La_3Zr_{1.2}Ta_{0.8}O_{12}$	25	3.20E-04
$La_{0.29}Li_{0.12}NbO_3$	25	4.25E-05	$Li_{1.4}Sc_{0.4}Hf_{1.6}(PO_4)_3$	25	6.17E-05	$Li_{6.30}P_{0.70}Si_{0.30}S_5Br$	25	1.83E-03
$La_{0.303}Li_{0.09}NbO_3$	25	3.52E-05	$Li_{1.3}Y_{0.3}Hf_{1.7}(PO_4)_3$	25	3.19E-05	$Li_{6.35}P_{0.65}Si_{0.35}S_5Br$	25	2.30E-03
$La_{0.313}Li_{0.06}NbO_3$	25	2.33E-05	$Li_{1.3}Y_{0.3}Ti_{1.7}(PO_4)_3$	25	5.54E-04	$Li_{6.46}Al_{0.15}La_3Zr_{1.95}O_{11.86}$	25	8.30E-05
$La_{0.51}Li_{0.36}Ti_{0.95}W_{0.05}O_3$	25	7.30E-04	$Li_{1.40}Al_{0.4}Ge_{0.2}Ti_{1.4}(PO_4)_3$	25	1.00E-03	$Li_{6.4}Al_{0.19}La_3Zr_2O_{11.8}$	25	3.10E-04
$La_{0.54}Li_{0.36}TiO_3$	25	8.90E-04	$Li_{1.45}Al_{0.45}Ge_{0.2}Ti_{1.35}(PO_4)_3$	25	1.01E-03	$Li_{6.4}La_3Zr_{1.4}Sb_{0.6}O_{12}$	30	6.60E-04
$La_{0.55}Li_{0.361}Ti_{0.995}Al_{0.005}O_3$	35	1.10E-03	$Li_{1.45}Al_{0.45}Ti_{1.55}(PO_4)$	25	2.90E-04	$Li_{6.4}La_3Zr_{1.4}Ta_{0.6}O_{12}$	34	2.06E-03
$La_{0.55}Li_{0.36}Ti_{0.95}Ge_{0.05}O_3$	25	3.60E-04	$Li_{1.4}Al_{0.4}(Ge_{0.67}Ti_{0.33})_{1.6}(PO_4)_3$	21	6.76E-04	$Li_{6.4}La_3Zr_2Ga_{0.2}O_{12}$	24	9.00E-04
$La_{0.55}Li_{0.36}Ti_{0.95}Mn_{0.05}O_3$	25	1.90E-04	$Li_{1.4}Al_{0.4}Ge_{0.2}Ti_{1.4}(PO_4)_3$	25	1.29E-03	$Li_{6.50}P_{0.50}Si_{0.50}S_5Br$	25	2.30E-03
$La_{0.55}Li_{0.36}Ti_{0.95}Ru_{0.05}O_3$	25	5.20E-05	$Li_{1.4}Al_{0.4}Ge_{0.8}Ti_{0.8}(PO_4)_3$	25	6.00E-05	$Li_{6.55}La_3Zr_2Ga_{0.9}O_{12}$	25	1.30E-03

$La_{0.55}Li_{0.36}Ti_{0.992}Al_{0.008}O_3$	25	6.54E-04	$Li_{1.4}Al_{0.4}Ge_{1.0}Ti_{0.6}(PO_4)_3$	25	8.00E-05	$Li_{6.5}La_{2.9}Ba_{0.1}Zr_{1.4}Ta_{0.6}O_{12}$	27	8.30E-04
$La_{0.55}Li_{0.36}Ti_{0.995}Al_{0.005}O_3$	25	1.10E-03	$Li_{1.4}Al_{0.4}Ge_{1.2}Ti_{0.4}(PO_4)_3$	25	3.43E-04	$Li_{6.5}La_3Nb_{1.25}Y_{0.75}O_{12}$	25	2.70E-04
$La_{0.56}Li_{0.36}Ti_{0.95}Al_{0.05}O_3$	25	6.40E-04	$Li_{1.3}Eu_{0.3}Ti_{1.7}(PO_4)_3$	25	6.00E-05	$Li_{6.5}La_3Zr_{1.5}Ta_{0.5}O_{12}$	26	8.57E-04
$La_{0.57}Li_{0.29}TiO_3$	27	1.60E-03	$Li_{1.3}Fe_{0.3}Ge_{1.7}(PO_4)_3$	25	3.67E-05	$Li_{6.5}La_3Zr_{1.75}Te_{0.25}O_{12}$	30	1.07E-04
$La_{0.58}Li_{0.36}Ti_{0.95}Mg_{0.05}O_3$	25	2.10E-04	$Li_{1.3}Fe_{0.3}Hf_{1.7}(PO_4)_3$	25	5.40E-05	$Li_{6.6}La_{2.5}Y_{0.5}Zr_{1.6}Ta_{0.4}O_{12}$	27	2.26E-04
$Li_{0.022}La_{0.115}Ti_{0.165}Al_{0.055}O_{0.618}$	30	5.25E-04	$Li_{1.3}In_{0.3}Ge_{1.7}(PO_4)_3$	25	9.22E-06	$Li_{6.6}La_{2.75}Y_{0.25}Zr_{1.6}Ta_{0.4}O_{12}$	27	4.36E-04
$Li_{0.022}La_{0.117}Ti_{0.208}Al_{0.001}O_{0.626}$	30	1.26E-03	$Li_{1.3}In_{0.3}Hf_{1.7}(PO_4)_3$	25	7.60E-05	$Li_{6.6}La_{2.875}Y_{0.125}Zr_{1.6}Ta_{0.4}O_{12}$	27	3.17E-04
$Li_{0.022}La_{0.118}Ti_{0.2}Al_{0.004}O_{0.631}$	30	8.91E-04	$Li_{1.3}La_{0.3}Ti_{1.7}(PO_4)_3$	25	5.28E-04	$Li_{6.6}La_3Zr_{1.6}Sb_{0.4}O_{12}$	30	7.70E-04
$Li_{0.067}La_{0.64}TiO_{2.99}$	27	7.90E-05	$Li_{1.3}Lu_{0.3}Hf_{1.7}(PO_4)_3$	25	3.56E-05	$Li_{6.6}La_3Zr_{1.6}Ta_{0.4}O_{12}$	25	7.30E-04
$Li_{0.06}La_{0.66}Ti_{0.93}Al_{0.06}O_3$	27	1.68E-06	$Li_{1.3}Sc_{0.3}Ge_{1.7}(PO_4)_3$	25	2.98E-05	$Li_{6.75}La_3Zr_{1.75}Nb_{0.25}O_{12}$	25	8.00E-04
$Li_{0.10}La_{0.63}TiO_3$	27	7.90E-05	$Li_{1.3}Sc_{0.3}Hf_{1.7}(PO_4)_3$	25	8.32E-05	$Li_{6.75}La_3Zr_{1.75}Ta_{0.25}O_{12}$	25	7.63E-04
$Li_{0.10}La_{0.66}Ti_{0.90}Al_{0.10}O_3$	27	7.34E-06	$Li_{1.3}Sc_{0.3}Ti_{1.7}(PO_4)_3$	25	6.81E-04	$Li_{6.75}La_3Zr_{1.875}Te_{0.125}O_{12}$	30	3.77E-05
$Li_{0.15}La_{0.51}Sr_{0.15}TiO_3$	25	5.30E-05	$Li_{1.3}Al_{0.2}Y_{0.1}Ti_{1.7}(PO_4)_3$	25	1.20E-04	$Li_{6.7}La_3Zr_{1.7}Ta_{0.3}O_{12}$	30	1.03E-03
$Li_{0.15}La_{0.66}Ti_{0.85}Al_{0.15}O_3$	25	9.66E-06	$Li_{1.3}Al_{0.3}Ge_{1.7}(PO_4)_3$	25	3.90E-05	$Li_{6.8}La_3Zr_{1.8}Sb_{0.2}O_{12}$	30	5.90E-05
$Li_{0.1}Na_{0.1}La_{0.6}TiO_3$	34	6.15E-05	$Li_{1.3}Al_{0.3}Ti_{1.7}(PO_4)_3$	24	2.10E-03	$Li_{6.8}La_3Zr_{1.8}Ta_{0.2}O_{12}$	25	2.80E-04
$Li_{0.1}Sr_{0.8}Ti_{0.7}Nb_{0.3}O_3$	25	1.00E-08	$Li_{1.3}Cr_{0.3}Ge_{1.7}(PO_4)_3$	25	3.07E-05	$Li_6BaLa_2Nb_2O_{12}$	22	6.00E-06
$Li_{0.20}La_{0.66}Ti_{0.80}Al_{0.20}O_3$	25	4.28E-05	$Li_{1.3}Cr_{0.3}Hf_{1.7}(PO_4)_3$	25	5.26E-07	$Li_6BaLa_2Ta_2O_{12}$	22	2.88E-05
$Li_{0.245}La_{0.592}Ti_{0.98}Mn_{0.02}O_3$	25	1.00E-03	$Li_{1.3}Cr_{0.3}Ti_{1.7}(PO_4)_3$	25	3.53E-05	$Li_6CaLa_2Nb_2O_{12}$	22	1.60E-06
$Li_{0.24}La_{0.59}TiO_3$	26	1.21E-04	$Li_{1.8}Al_{0.8}Ge_{1.0}Ti_{0.2}(PO_4)_3$	25	3.00E-05	$Li_6CaLa_2Ta_2O_{12}$	27	2.24E-06
$Li_{0.25}La_{0.25}TaO_3$	25	3.66E-04	$Li_{1.8}Cr_{0.8}Ti_{1.2}(PO_4)_3$	25	2.40E-04	Li_6CoCl_8	25	1.51E-06
$Li_{0.25}La_{0.41}Sr_{0.25}TiO_3$	25	7.64E-05	$Li_{1.8}Sc_{0.8}Ti_{1.2}(PO_4)_3$	25	8.80E-04	Li_6FeCl_8	25	8.97E-08
$Li_{0.25}La_{0.66}Ti_{0.75}Al_{0.25}O_3$	25	7.66E-05	$Li_{1.9}Mg_{0.1}TiO_{3.05}$	25	1.96E-07	$Li_6La_2Ca(Nb)_2O_{12}$	25	2.11E-06
$Li_{0.27}La_{0.59}TiO_3$	25	5.80E-04	$Li_{10.35}Si_{1.35}P_{1.65}S_{12}$	25	6.50E-03	$Li_6La_2SrNb_2O_{12}$	25	6.21E-07
$Li_{0.2}Zr_{1.2}Nb_{0.8}(PO_4)_3$	25	2.38E-07	$Li_{10}Ge_{0.95}Si_{0.05}P_2S_{12}$	25	8.60E-03	$Li_6La_3Nb_{1.5}Y_{0.5}O_{12}$	24	1.88E-04
$Li_{0.30}La_{0.66}Ti_{0.70}Al_{0.30}O_3$	25	1.72E-05	$Li_{10}GeP_2S_{12}$	25	7.02E-03	$Li_6La_3ZrSbO_{12}$	30	2.60E-04
$Li_{0.33}La_{0.56}Ti_{0.96}Al_{0.04}O_{2.96}F_{0.04}$	25	6.22E-04	$Li_{10}SiP_2S_{12}$	27	2.30E-03	$Li_6La_3ZrTaO_{12}$	25	1.60E-04
$Li_{0.34}La_{0.51}TiO_{2.94}$	27	1.02E-03	$Li_{10}SnP_2S_{12}$	25	4.00E-03	$Li_6MgLa_2Ta_2O_{12}$	21	1.92E-07
$Li_{0.34}La_{0.56}TiO_3$	25	1.11E-03	$Li_{11}AlP_2S_{12}$	25	8.02E-04	Li_6NiBr_3	25	1.50E-07
$Li_{0.34}Pr_{0.56}TiO_3$	25	1.00E-06	$Li_{13.6}Si_{2.8}S_{1.2}O_{16}$	25	6.00E-07	Li_6NiCl_8	25	2.69E-10
$Li_{0.36}La_{0.55}TiO_3$	30	2.00E-05	$Li_{14}ZnGe_4O_{16}$	25	1.00E-06	Li_6PO_5Cl	25	1.00E-09
$Li_{0.3}Sr_{0.6}Ti_{0.20}Fe_{0.30}Ta_{0.5}O_3$	25	1.00E-07	$Li_{2.1}OH_{0.9}Cl$	26	8.05E-08	Li_6PS_5Br	25	4.42E-04
$Li_{0.3}Sr_{0.6}Ti_{0.35}Fe_{0.15}Ta_{0.5}O_3$	25	2.80E-05	$Li_{2.2}Zn_{0.1}Zr_{0.9}S_3$	30	1.20E-04	Li_6PS_5Cl	22	4.57E-04
$Li_{0.3}Sr_{0.6}Ti_{0.40}Fe_{0.10}Ta_{0.5}O_3$	25	3.60E-05	$Li_{2.4}OH_{0.6}Cl$	26	8.90E-08	Li_6PS_5I	25	6.30E-04
$Li_{0.3}Sr_{0.6}Ti_{0.45}Fe_{0.05}Ta_{0.5}O_3$	30	6.00E-05	$Li_{2.7}OH_{0.3}Cl$	26	7.30E-09	$Li_6Sr_{0.5}Ba_{0.5}La_2Ta_2O_{12}$	27	7.27E-06
$Li_{0.3}Sr_{0.6}Ti_{0.5}Nb_{0.5}O_3$	30	5.40E-06	$Li_{2.9}Sc_{1.9}Zr_{0.1}(PO_4)_3$	25	7.48E-05	$Li_6SrLa_2Nb_2O_{12}$	22	4.20E-06
$Li_{0.3}Sr_{0.6}Ti_{0.5}Ta_{0.5}O_3$	30	1.70E-04	$Li_2Ca(NH)_2$	25	6.40E-06	$Li_6SrLa_2Ta_2O_{12}$	28	9.27E-06
$Li_{0.5}(La_{0.4}Nd_{0.1})TiO_3$	25	1.00E-03	$Li_2CrTi(PO_4)_3$	25	4.41E-07	$Li_{7.06}La_3Zr_{1.94}Y_{0.06}O_{12}$	25	9.56E-04
$Li_{0.5}La_{0.5}TiO_3$	21	2.99E-05	$Li_2CrZr(PO_4)_3$	25	5.91E-07	$Li_7Ge_3PS_{12}$	25	1.10E-04
$Li_{0.5}Sr_{0.5}Cr_{0.25}Ta_{0.75}O_3$	25	6.31E-05	$Li_2FeTi(PO_4)_3$	25	3.34E-07	$Li_7La_3Hf_2O_{12}$	27	9.73E-07
$Li_{0.5}Sr_{0.5}Fe_{0.25}Ta_{0.75}O_3$	25	1.26E-04	$Li_2FeZr(PO_4)_3$	25	1.44E-07	$Li_7La_3Ta_2O_{13}$	30	2.65E-06
$Li_{0.7}Al_{0.3}Ti_{1.7}(PO_4)_3$	22	3.33E-04	$Li_2InHf(PO_4)_3$	25	2.74E-08	$Li_7La_3Zr_{1.89}Al_{0.15}O_{12}$	25	3.40E-04
$Li_{0.83}Mg_{0.83}In_{0.17}PO_4$	25	1.41E-09	$Li_2InTi(PO_4)_3$	25	8.30E-06	$Li_7La_3Zr_2O_{12}$	26	1.34E-04
$Li_{0.85}Mg_{0.85}In_{0.15}PO_4$	25	1.26E-09	$Li_2InZr(PO_4)_3$	25	8.22E-08	$Li_7P_3S_{11}$	25	2.42E-03
$Li_{0.8}La_{0.066}Zr_2(PO_4)_3$	25	8.20E-06	Li_2NH	25	2.54E-04	$Li_{9.54}Si_{1.74}P_{1.44}S_{11.7}Cl_{0.3}$	25	2.50E-02
$Li_{0.95}Mg_{0.95}In_{0.05}PO_4$	25	1.12E-10	Li_2OHBr	22	5.40E-07	$Li_{9.6}P_3S_{12}$	25	1.58E-03
$Li_{0.98}Zr_{1.98}Nb_{0.02}(PO_4)_3$	25	2.50E-07	Li_2OHCl	26	7.11E-08	$Li_{9.81}Sn_{0.81}P_{2.19}S_{12}$	25	5.50E-03
$Li_{0.9}Mg_{0.9}In_{0.1}PO_4$	25	5.01E-10	Li_2SiS_3	25	2.00E-06	Li_9SiAlO_8	30	2.30E-07
$Li_{0.9}Zr_{1.9}Nb_{0.1}(PO_4)_3$	25	1.35E-06	Li_2SnS_3	25	1.36E-05	$LiAlH_4$	25	2.00E-09
$Li_{1.02}Zr_{1.98}Y_{0.02}(PO_4)_3$	25	1.15E-07	Li_2ZrS_3	30	7.30E-06	$LiAlSb(PO_4)_3$	30	3.17E-08
$Li_{1.05}Al_{0.05}Ti_{1.95}(PO_4)$	25	1.40E-06	$Li_{3.09}BO_{2.53}N_{0.52}$	25	8.65E-07	$LiAlSi_2O_6$	25	3.49E-09
$Li_{1.076}Al_{0.6923}Ti_{1.462}(PO_4)_3$	21	8.25E-04	$Li_{3.25}Ge_{0.25}P_{0.75}S_4$	25	2.12E-03	$LiAlTa(PO_4)_3$	30	6.50E-07
$Li_{1.15}Fe_{0.15}Ti_{1.85}(PO_4)_3$	25	5.22E-04	$Li_{3.2}Ge_{0.2}P_{0.8}O_4$	30	1.14E-06	$LiBa_2Ti_2NbO_9$	30	1.00E-07
$Li_{1.15}Y_{0.15}Zr_{1.85}(PO_4)_3$	25	7.10E-05	$Li_{3.2}Ge_{0.2}P_{0.8}S_4$	27	5.40E-04	$LiBH_4$	27	9.89E-08
$Li_{1.1}Al_{0.1}Ge_{1.9}(PO_4)_3$	25	2.90E-06	$Li_{3.2}Ge_{0.2}V_{0.8}O_4$	30	1.90E-05	$LiCaTiNbO_6$	25	6.07E-07

$Li_{1.1}Al_{0.1}Ti_{1.9}(PO_4)_3$	25	3.00E-05	$Li_{3.2}PO_{3.1}N_{0.7}$	25	5.00E-07	$LiCrSb(PO_4)_3$	30	3.57E-10
$Li_{1.1}Cr_{0.1}Hf_{1.9}(PO_4)_3$	25	9.35E-08	$Li_{3.35}Ge_{0.35}P_{0.65}S_4$	25	1.83E-03	$LiFePO_4$	30	1.10E-09
$Li_{1.1}Fe_{0.1}Hf_{1.9}(PO_4)_3$	25	1.07E-04	$Li_{3.4}Ge_{0.4}P_{0.6}O_4$	30	4.50E-06	$LiFeSb(PO_4)_3$	30	1.37E-06
$Li_{1.1}In_{0.1}Hf_{1.9}(PO_4)_3$	25	1.00E-06	$Li_{3.4}Ge_{0.4}V_{0.6}O_4$	30	8.89E-07	$LiGe_2(PO_4)_3$	23	8.60E-07
$Li_{1.1}Lu_{0.1}Hf_{1.9}(PO_4)_3$	25	5.88E-05	$Li_{3.4}Si_{0.4}P_{0.6}S_4$	27	6.40E-04	$LiHf_2(PO_4)_3$	25	3.80E-06
$Li_{1.1}Sc_{0.1}Hf_{1.9}(PO_4)_3$	25	2.45E-05	$Li_{3.5}Ge_{0.5}V_{0.5}O_4$	25	7.00E-05	$LiHfTi(PO_4)_3$	23	5.63E-07
$Li_{1.1}Sc_{0.1}Ti_{1.9}(PO_4)_3$	25	1.58E-04	$Li_{3.6}Ge_{0.4}P_{0.6}S_4$	25	2.21E-04	LiI	25	2.80E-08
$Li_{1.1}Y_{0.1}Hf_{1.9}(PO_4)_3$	25	9.18E-05	$Li_{3.6}Ge_{0.6}P_{0.4}O_4$	30	1.10E-05	$LiLa(TiO_3)_2$	25	1.00E-03
$Li_{1.2}Al_{0.2}Ge_{0.8}Ti(PO_4)_3$	25	3.00E-05	$Li_{3.6}Ge_{0.6}V_{0.4}O_4$	30	3.19E-05	$LiP_{0.9}I_{0.1}O_{2.7}$	25	3.98E-04
$Li_{1.2}Al_{0.2}Ge_{1.0}Ti_{0.8}(PO_4)_3$	25	3.00E-05	$Li_{3.6}Si_{0.6}P_{0.4}S_4$	25	7.76E-05	$LiSn_2(PO_4)_3$	25	2.00E-09
$Li_{1.2}Al_{1.1}Ta_{0.9}(PO_4)_3$	30	6.50E-07	$Li_{3.75}Si_{0.75}P_{0.25}O_4$	25	5.54E-07	$LiSr_{1.65}Ti_{1.3}Nb_{1.7}O_9$	25	2.00E-05
$Li_{1.2}Cr_{0.2}Hf_{1.8}(PO_4)_3$	25	2.54E-07	$Li_{3.8}Ge_{0.8}P_{0.2}O_4$	30	1.03E-06	$LiSr_{1.65}Ti_{1.3}Ta_{1.7}O_9$	25	4.90E-05
$Li_{1.2}Cr_{0.2}Ti_{1.8}(PO_4)_3$	25	1.83E-05	$Li_{3.8}Ge_{0.8}V_{0.2}O_4$	30	2.71E-06	$LiSr_{1.65}Zr_{1.3}Ta_{1.7}O_9$	25	1.30E-05
$Li_{1.2}Fe_{0.2}Hf_{1.8}(PO_4)_3$	25	1.70E-04	$Li_{3.9}Zn_{0.05}GeS_4$	25	2.84E-07	$LiSr_2Ti_2NbO_9$	25	1.00E-06
$Li_{1.2}Fe_{0.2}Ti_{1.8}(PO_4)_3$	25	3.51E-04	$Li_3Al_{0.4}Sc_{1.6}(PO_4)_3$	25	1.28E-05	$LiSr_2Ti_2TaO_9$	30	3.20E-05
$Li_{1.2}In_{0.2}Hf_{1.8}(PO_4)_3$	25	1.32E-04	Li_3AlS_3	25	1.31E-08	$LiSrTiNbO_6$	25	1.00E-06
$Li_{1.2}In_{0.2}Ti_{1.8}(PO_4)_3$	25	4.01E-04	$Li_3Cr(MoO_4)_3$	25	3.19E-09	$LiSrTiTaO_6$	25	5.50E-04
$Li_{1.2}La_{0.2}Ti_{1.8}(PO_4)_3$	25	5.28E-04	$Li_3Fe(MoO_4)_3$	25	5.89E-10	$LiTa_2PO_8$	25	1.60E-03
$Li_{1.2}Lu_{0.2}Hf_{1.8}(PO_4)_3$	25	9.79E-05	$Li_3Fe_2P_3O_{12}$	25	1.11E-08	$LiTi_{0.1}Zr_{1.9}(PO_4)_3$	25	3.20E-08
$Li_{1.2}Sc_{0.2}Hf_{1.8}(PO_4)_3$	25	5.86E-05	Li_3N	25	5.75E-04	$LiTi_{0.2}Zr_{1.8}(PO_4)_3$	25	5.60E-08
$Li_{1.2}Sc_{0.2}Ti_{0.2}Zr_{1.5}(PO_4)_3$	25	3.10E-07	$Li_3O(Cl_{0.5}Br_{0.5})$	30	9.38E-04	$LiTi_{0.5}Zr_{1.5}(PO_4)_3$	25	6.20E-08
$Li_{1.2}Sc_{0.2}Ti_{1.6}Zr_{0.2}(PO_4)_3$	25	1.20E-04	Li_3OBr	25	1.06E-06	$LiTi_{1.8}Zr_{0.2}(PO_4)_3$	25	8.50E-08
$Li_{1.2}Sc_{0.2}Ti_{1.8}(PO_4)_3$	25	2.46E-04	Li_3P	25	1.00E-03	$LiTi_2(PO_4)_3$	25	3.62E-05
$Li_{1.2}Ti_{1.8}Sc_{0.2}(PO_4)_3$	25	2.31E-04	Li_3PS_4	25	1.60E-04	$LiTi_2P_3O_{12}$	30	4.00E-07
$Li_{1.2}Y_{0.2}Hf_{1.8}(PO_4)_3$	25	3.19E-05	Li_3VO_4	28	8.35E-07	$LiTiZr(PO_4)_3$	25	4.00E-08
$Li_{1.2}Y_{0.2}Ti_{1.8}(PO_4)_3$	25	5.04E-04	$Li_{4.25}Si_{0.75}Al_{0.25}O_4$	25	2.02E-08	$LiZr_2(PO_4)_3$	25	1.68E-06
$Li_{1.35}Al_{0.35}Ge_{0.2}Ti_{1.45}(PO_4)_3$	25	6.18E-04	$Li_{4.275}Ge_{0.61}Ga_{0.25}S_4$	25	6.34E-05	$LiZr_2P_3O_{12}$	30	5.00E-08
$Li_{1.3}Al_{0.15}Y_{0.15}Ti_{1.7}(PO_4)_3$	25	1.90E-05	$Li_{4.2}Ge_{0.8}Ga_{0.2}S_4$	25	6.50E-05	$Nd_{0.25}Li_{0.25}TaO_3$	25	1.91E-06
$Li_{1.3}Al_{0.1}Sc_{0.2}Ti_{1.7}(PO_4)_3$	25	4.26E-04	$Li_{4.4}Al_{0.4}Ge_{0.6}S_4$	30	4.33E-05	$Nd_{0.55}Li_{0.34}TiO_3$	25	8.00E-08
$Li_{1.3}Al_{0.1}Y_{0.2}Ti_{1.7}(PO_4)_3$	25	7.30E-05	$Li_{4.4}Al_{0.4}Sn_{0.6}S_4$	30	4.39E-06	$Sm_{0.52}Li_{0.38}TiO_{2.97}$	25	1.50E-03
$Li_{1.3}Al_{0.2}Sc_{0.1}Ti_{1.7}(PO_4)_3$	25	2.69E-04	$Li_{4.4}Ga_{0.4}Ge_{0.6}S_4$	30	1.63E-05			

Table S2. Elemental count in the computational (source) and experimental data (target). The count here refers to the number of times an element appears in the compounds, while the ratio is the frequency of its appearance in these compounds (i.e., the count divided by the number of compounds (8,750 and 398 respectively for source and target data)).

Element	Computational data (source)		Experimental data (target)		Element	Computational data (source)		Experimental data (target)	
	Count	Ratio	Count	Ratio		Count	Ratio	Count	Ratio
**Ag**	89	0.010	1	0.003	**Mo**	169	0.019	2	0.005
**Al**	234	0.027	75	0.188	**N**	358	0.041	7	0.018
**As**	132	0.015	0	0.000	**Na**	263	0.030	2	0.005
**Au**	102	0.012	0	0.000	**Nb**	343	0.039	27	0.068
**B**	407	0.047	7	0.018	**Nd**	91	0.010	5	0.013
**Ba**	130	0.015	6	0.015	**Ni**	777	0.089	2	0.005
**Be**	46	0.005	0	0.000	**O**	6072	0.694	316	0.794
**Bi**	191	0.022	1	0.003	**Os**	46	0.005	0	0.000
**Br**	84	0.010	18	0.045	**P**	1549	0.177	214	0.538
**C**	355	0.041	0	0.000	**Pb**	69	0.008	0	0.000
**Ca**	128	0.015	5	0.013	**Pd**	96	0.011	0	0.000
**Cd**	77	0.009	0	0.000	**Pm**	29	0.003	0	0.000
**Ce**	64	0.007	0	0.000	**Pr**	69	0.008	1	0.003
**Cl**	200	0.023	15	0.038	**Pt**	76	0.009	0	0.000
**Co**	792	0.091	1	0.003	**Rb**	149	0.017	0	0.000
**Cr**	771	0.088	16	0.040	**Re**	31	0.004	0	0.000
**Cs**	145	0.017	0	0.000	**Rh**	90	0.010	0	0.000
**Cu**	515	0.059	0	0.000	**Ru**	76	0.009	1	0.003
**Dy**	42	0.005	0	0.000	**S**	401	0.046	75	0.188
**Er**	44	0.005	0	0.000	**Sb**	370	0.042	14	0.035
**Eu**	59	0.007	1	0.003	**Sc**	79	0.009	18	0.045
**F**	881	0.101	2	0.005	**Se**	82	0.009	1	0.003
**Fe**	1203	0.137	21	0.053	**Si**	522	0.060	35	0.088
**Ga**	164	0.019	14	0.035	**Sm**	53	0.006	1	0.003
**Gd**	43	0.005	0	0.000	**Sn**	393	0.045	7	0.018
**Ge**	221	0.025	62	0.156	**Sr**	140	0.016	22	0.055
**H**	411	0.047	12	0.030	**Ta**	84	0.010	36	0.090
**Hf**	25	0.003	28	0.070	**Tb**	33	0.004	0	0.000
**Hg**	70	0.008	0	0.000	**Tc**	15	0.002	0	0.000
**Ho**	42	0.005	0	0.000	**Te**	198	0.023	3	0.008
**I**	68	0.008	11	0.028	**Ti**	656	0.075	133	0.334

**In**	159	0.018	18	0.045	**Tl**	103	0.012	0	0.000
**Ir**	85	0.010	0	0.000	**Tm**	44	0.005	0	0.000
**K**	221	0.025	1	0.003	**V**	988	0.113	6	0.015
**La**	135	0.015	105	0.264	**W**	230	0.026	1	0.003
**Li**	8750	1.000	398	1.000	**Y**	109	0.012	23	0.058
**Lu**	44	0.005	4	0.010	**Yb**	54	0.006	0	0.000
**Mg**	218	0.025	8	0.020	**Zn**	143	0.016	3	0.008
**Mn**	1261	0.144	2	0.005	**Zr**	82	0.009	50	0.126

Table S3. Performance on the computational and experimental data, based on from scratch (FS) training and transfer learning (TL). The mean and standard deviation (S. D.) on training data were calculated based on ten-fold cross-validation. The units for RMSE and MAE of experimental data are S cm^{-1} at log_{10} scale.

Model	Computational data			Experimental data (FS)			Experimental data (TL)		
	RMSE	MAE	R^2	RMSE	MAE	R^2	RMSE	MAE	R^2
m_g	0.169 ±0.004	0.115 ±0.004	0.627 ±0.019	0.976 ±0.112	0.746 ±0.102	0.718 ±0.049	0.912 ±0.060	0.710 ±0.058	0.742 ±0.026
m_e	0.194 ±0.011	0.141 ±0.012	0.507 ±0.057	0.945 ±0.056	0.724 ±0.053	0.722 ±0.029	1.009 ±0.062	0.778 ±0.059	0.696 ±0.018
m_t	0.141 ±0.004	0.095 ±0.003	0.738 ±0.014	0.768 ±0.061	0.586 ±0.064	0.814 ±0.025	0.746 ±0.049	0.558 ±0.037	0.830 ±0.017
m_g+m_e	0.165 ±0.006	0.116 ±0.005	0.647 ±0.024	0.818 ±0.039	0.617 ±0.033	0.197 ±0.019	0.806 ±0.040	0.616 ±0.051	0.794 ±0.016
m_g+m_t	0.129 ±0.003	0.087 ±0.003	0.781 ±0.013	0.747 ±0.050	0.565 ±0.046	0.832 ±0.019	0.708 ±0.062	0.532 ±0.058	0.842 ±0.023
m_e+m_t	0.127 ±0.007	0.085 ±0.005	0.781 ±0.018	0.745 ±0.055	0.560 ±0.043	0.835 ±0.018	0.696 ±0.057	0.530 ±0.059	0.847 ±0.014
m_g+m_e+m_t (IonNet)	**0.068 ±0.005**	**0.046 ±0.003**	**0.940 ±0.009**	0.743 ±0.063	0.565 ±0.058	0.836 ±0.016	**0.679 ±0.045**	**0.503 ±0.049**	**0.858 ±0.022**

Table S4. Domain discrepancy between the source (computational) and target (experimental) datasets. The maximum mean discrepancy and Wasserstein distance in both the raw feature space and the learned embedding space were calculated.

Model	Source data (shape)	Target data (shape)	Maximum mean discrepancy	Wasserstein distance
**Raw feature**	(8750, 332)	(398, 332)	50272.7469	3.0786
**Embedding**	(8750, 48)	(398, 48)	37.6475	0.7132

Table S5. Average gradient (AG) of each feature of m_g, m_e, and m_t. This highlights the features that IonNet prioritizes during the decision-making process. Since descriptors are introduced into different sub-networks, only the absolute values of gradients are compared under the same descriptor.

m_e				m_g				m_t			
**index**	**gradient**	**index**	**gradient**	**index**	**gradient**	**index**	**gradient**	**index**	**gradient**	**index**	**gradient**
1	-0.32519	61	2.26372	1	0.00452	67	0.00137	1	-2.37964	41	-2.37586
2	-0.32939	62	-23.44811	2	-0.00431	68	0.00473	2	-3.14702	42	-0.48613
3	2.83259	63	-11.92705	3	-0.00489	69	0.00383	3	0.84256	43	0.26821
4	-11.70007	64	8.64595	4	0.00154	70	0.02195	4	-4.68429	44	4.60288
5	-1.00151	65	-7.10372	5	0.00138	71	0.02391	5	1.01252	45	-0.36486
6	2.12221	66	-0.71591	6	-0.00588	72	-0.00258	6	2.52843	46	-0.83769
7	-7.9203	67	-3.78052	7	0.00296	73	0.00151	7	3.74665	47	-5.66882
8	1.08244	68	-4.21332	8	0.00101	74	-0.01443	8	1.14292	48	-1.58516
9	-17.31703	69	0.24095	9	-0.00026	75	-0.01617	9	0.65338	49	-1.12002
10	-0.21007	70	3.51168	10	0.00153	76	0.00377	10	0.42857	50	-0.62947
11	-24.07261	71	-1.87005	11	0.00457	77	0.00456	11	0.43474	51	-0.13589
12	24.78745	72	6.3408	12	-0.00772	78	-0.00542	12	-2.44915	52	0.96618
13	-9.50375	73	-13.2352	13	0.00129	79	-0.00701	13	-2.28421	53	1.32926
14	5.19032	74	-18.82942	14	-0.0059	80	0.00181	14	3.4137	54	-0.05741
15	-5.19009	75	5.21536	15	-0.00484	81	0.00463	15	3.13694	55	-0.06506
16	-6.82832	76	-0.09073	16	0.0023	82	0.00604	16	0.87382	56	-1.77688
17	3.10912	77	-4.12078	17	0.000467	83	0.00701	17	0.54663	57	-2.117
18	0.38534	78	2.84225	18	-0.00549	84	-0.00077	18	-0.39321	58	-0.02012
19	1.07528	79	1.10724	19	0.00546	85	0.00176	19	5.02266	59	-1.64476
20	18.57793	80	-0.21899	20	0.00164	86	0.00368	20	-1.61457	60	2.25081
21	-29.24575	81	-1.18475	21	0.000433	87	0.00463	21	0.26552	61	2.05305
22	-14.02271	82	-3.47181	22	-0.00195	88	0.01272	22	-0.26591	62	0.14149
23	3.33387	83	0.61566	23	-0.00051	89	0.00766	23	-1.18987	63	-1.55114
24	12.3105	84	0.07859	24	-0.00277	90	-0.00671	24	0.34908	64	1.59639
25	-9.74757	85	0.08738	25	0.00328	91	-0.00018	25	-1.03151	65	-0.57118
26	13.72414	86	-0.1113	26	0.00221	92	-0.01713	26	-2.00858	66	-1.17923
27	-8.02622	87	0.11032	27	0.00088	93	-0.01657	27	-0.14696	67	-2.09131
28	22.03631	88	0.02141	28	0.00273	94	-0.0368	28	0.24621	68	-0.28488
29	-0.8132	89	-0.33059	29	0.00241	95	-0.02531	29	-3.97556	69	2.02134
30	-8.30039	90	0.14383	30	-0.00471	96	-0.01742	30	0.36654	70	-1.24956
31	-10.08382	91	-0.0037	31	0.00155	97	-0.00108	31	-0.95725	71	2.59564
32	-12.68223	92	-0.36133	32	-0.00124	98	0.05207	32	1.24452	72	0.63592
33	-3.68771	93	-0.04232	33	-0.00254	99	0.05135	33	1.6366	73	-1.72229
34	-2.47099	94	-0.02746	34	0.003	100	0.07857	34	-1.58181	74	0.50971
35	-2.71916	95	0.0056	35	-0.00076	101	0.08274	35	0.28831	75	2.84432
36	0.0923	96	0.10539	36	0.0021	102	0.00408	36	1.2011	76	0.17497
37	-5.92958	97	0.0536	37	-0.00004	103	-0.01332	37	0.33236	77	-2.02733
38	2.39682	98	-0.14943	38	0.000285	104	0.00609	38	-0.383	78	-1.80358

39	0.53013	99	-0.25538	39	-0.00292	105	0.0092	39	-0.36276	79	-4.35905
40	12.24813	100	-0.2049	40	0.00206	106	0.00329	40	0.91649	80	1.94168
41	-6.88223	101	-0.13032	41	0.00667	107	-0.0044				
42	12.28139	102	0.40524	42	0.00113	108	-0.00535				
43	5.56244	103	-0.06919	43	0.00234	109	0.00042				
44	-1.75167	104	0.35047	44	-0.00155	110	-0.00583				
45	-1.84222	105	-0.03044	45	-0.0017	111	-0.01253				
46	1.75575	106	-0.07478	46	0.00197	112	-0.00087				
47	1.24703	107	-0.20209	47	0.00352	113	0.00766				
48	-2.09382	108	0.26185	48	-0.00392	114	-0.00331				
49	0.61184	109	-1.89369	49	0.00538	115	0.00145				
50	7.31932	110	0.81001	50	0.00352	116	0.0036				
51	13.05463	111	0.38682	51	0.00352	117	0.00151				
52	10.45509	112	0.60949	52	0.00112	118	-0.0027				
53	-1.93883	113	0.01967	53	0.00483	119	0.00181				
54	0.17422	114	0.04199	54	-0.00341	120	-0.01802				
55	-2.23852	115	1.0182	55	-0.00109	121	-0.0017				
56	4.42554	116	-2.28665	56	-0.00556	122	0.01226				
57	-4.2134	117	0.05581	57	-0.00416	123	0.01128				
58	-2.38201	118	0.30264	58	-0.00073	124	0.04557				
59	15.07411	119	0.50648	59	-0.00093	125	0.04636				
60	15.87243	120	1.13466	60	-0.01018	126	0.02313				
				61	0.000369	127	0.02075				
				62	-0.02436	128	0.00413				
				63	-0.0255	129	-0.0041				
				64	0.00867	130	0.00327				
				65	0.00188	131	0.00297				
				66	0.00711	132	-0.00416				

Table S6. Selected 102 electrolyte candidates. Materials project ID, chemical composition, energy above hull (E_{hull}: eV/atom), band gap (E_g: eV), ionic conductivity (σ: S cm^{-1}), ionic conductivity plus standard deviation (σ_{SD}: S cm^{-1}), and other notes (whether the electrolyte is known or in our compiled dataset or is selected for AIMD validation) are listed.

Group index	Materials project ID	Composition	E_{hull}	E_g	σ	σ_{SD}	Note
1	mp-760419	Li_2BiF_5	0.06	4.06	2.46E-01	1.72E-02	New FC, selected for AIMD
2	mp-1110656	Rb_2LiBiF_6	0.01	4.97	1.69E-01	6.57E-03	New FC
3	mp-769026	Li_2BiO_3	0.05	1.04	4.08E-02	5.48E-03	New FC, selected for AIMD
4	mp-1110838	K_2LiBiF_6	0.06	4.88	1.43E-01	4.56E-03	New FC
5	mp-28567	$LiBiF_4$	0.00	4.85	6.39E-02	4.09E-03	New FC, selected for AIMD
6	mp-756718	$Li_4Bi_2O_5$	0.03	2.68	3.44E-02	4.00E-03	New FC, selected for AIMD
7	mp-1097035	$Li_{20}Si_3P_3S_{23}Cl$	0.03	2.31	5.06E-03	3.10E-03	New FC, selected for AIMD
8	mp-1110629	Na_2LiTlF_6	0.06	3.37	3.32E-02	2.05E-03	New FC
9	mp-721236	$Li_{10}Sn(PS_6)_2$	0.02	2.34	3.03E-03	1.72E-03	Known FC with σ of 4.00E-03
10	mp-1111658	K_2LiTlF_6	0.01	3.51	3.98E-02	1.45E-03	New FC
11	mp-557738	Li_3BiO_4	0.00	1.06	1.28E-02	9.67E-04	Known FC with σ of 4.1E-10 and activation energy of 0.37 eV
12	mp-720509	$Li_{10}Si(PS_6)_2$	0.01	2.56	1.60E-03	8.88E-04	New FC, selected for AIMD
13	mp-696138	$Li_{10}Ge(PS_6)_2$	0.02	2.37	1.34E-03	8.76E-04	Known FC with σ of 1.2E-03
14	mp-1113693	Rb_2LiTlF_6	0.00	3.47	2.58E-02	8.13E-04	New FC
15	mp-985592	Li_6PS_5Cl	0.08	2.14	1.43E-03	8.02E-04	Known FC with σ of 3.15E-03
16	mp-504854	$Na_2Li_3CoO_4$	0.00	1.46	2.86E-03	7.52E-04	New FC
17	mp-644419	LiHS	0.00	3.53	1.35E-03	6.53E-04	New FC, selected for AIMD
18	mp-28510	Li_3BiO_3	0.00	2.44	1.50E-02	6.06E-04	Known FC with σ of 2.4E-10 and activation energy of 0.33 eV
19	mp-555439	$CsLi_3F_4$	0.07	6.74	4.95E-02	5.76E-04	New FC
20	mp-1206880	Li_2HgO_2	0.00	1.18	1.20E-02	5.69E-04	New FC
21	mp-1110976	Cs_2LiBiF_6	0.01	4.89	2.37E-02	3.77E-04	New FC
22	mp-721303	$LiBeH_5(NF_2)_2$	0.09	5.79	8.58E-04	3.68E-04	New FC
23	mp-776687	Li_5FeF_8	0.04	3.86	1.30E-03	3.68E-04	New FC
24	mp-772968	$LiTlPHO_3$	0.00	3.74	6.69E-03	3.60E-04	New FC
25	mp-776129	Li_3FeF_5	0.05	4.56	1.22E-03	3.36E-04	New FC
26	mp-780155	$Li_9Fe_3(OF_7)_2$	0.05	1.81	1.03E-03	3.36E-04	New FC
27	mp-861533	$Na_2Li_2CoO_4$	0.06	0.63	8.88E-04	3.33E-04	New FC, selected for AIMD
28	mp-1191085	$LiY(WO_4)_2$	0.00	3.78	9.98E-04	3.33E-04	New FC
29	mp-776627	Li_3FeF_6	0.00	3.68	1.26E-03	3.22E-04	New FC
30	mp-1001069	$Li_{48}P_{16}S_{61}$	0.02	2.30	4.01E-04	3.17E-04	New FC
31	mp-1540429	$LiBiO_2$	0.00	2.52	2.24E-03	3.02E-04	Known FC with σ of 3.80E-08 and activation energy of 0.10 eV
32	mp-985583	Li_3PS_4	0.00	2.81	3.73E-04	2.95E-04	Known FC with σ of 1.60E-04, in dataset

**33**	mp-1278660	Li_4CoOF_5	0.08	0.53	1.77E-03	2.95E-04	New FC, selected for AIMD
**34**	mp-1178085	$Li_{11}Fe_5(OF_{11})_2$	0.03	2.32	9.48E-04	2.85E-04	New FC
**35**	mp-1180504	LiH_2IO	0.00	3.72	2.56E-03	2.84E-04	New FC
**36**	mp-1198596	$Li_2P_7(H_3N)_9$	0.00	2.09	5.76E-04	2.82E-04	New FC
**37**	mp-606604	$CsLi_2F_3$	0.06	7.04	1.85E-02	2.71E-04	New FC, selected for AIMD
**38**	mp-1040450	$Li_5P(S_2Cl)_2$	0.02	2.78	5.91E-04	2.52E-04	New FC, selected for AIMD
**39**	mp-1199210	$Li_3MnP_2(O_3F_2)_2$	0.00	1.75	1.25E-03	2.52E-04	New FC
**40**	mp-756952	Li_4MnF_8	0.05	2.24	9.51E-04	2.50E-04	New FC
**41**	mp-722502	$Li_3P_{11}(H_3N)_{17}$	0.01	1.97	5.31E-04	2.42E-04	New FC
**42**	mp-1178101	$Li_{10}Sc_9Fe(SiO_3)_{20}$	0.01	3.06	5.08E-04	2.35E-04	New FC
**43**	mp-759724	Li_3MnF_7	0.06	1.28	7.78E-04	2.31E-04	New FC
**44**	mp-760028	Li_3MnF_6	0.00	1.72	8.92E-04	2.27E-04	New FC
**45**	mp-950995	Li_6PS_5I	0.00	2.27	5.02E-04	2.27E-04	Known FC with σ of 6.30E-04, in dataset
**46**	mp-706611	$LiBeH_4NF_4$	0.02	6.73	5.62E-04	2.15E-04	New FC
**47**	mp-755600	Li_4MnF_7	0.03	1.53	9.69E-04	2.03E-04	New FC
**48**	mp-760046	$LiPH_{21}S_3N_7$	0.00	3.06	4.57E-04	2.02E-04	New FC
**49**	mp-641703	$Li_7P_3S_{11}$	0.02	2.41	2.68E-04	1.95E-04	Known FC with σ of 2.42E-03, in dataset
**50**	mp-556229	Li_2MnF_5	0.00	1.84	6.29E-04	1.85E-04	New FC
**51**	mp-27419	$LiBiF_6$	0.00	2.54	5.93E-03	1.83E-04	New FC
**52**	mp-764088	$Li_6Co_2OF_{11}$	0.07	0.98	1.04E-03	1.78E-04	New FC
**53**	mp-1177082	Li_5MnF_8	0.04	1.98	8.72E-04	1.66E-04	New FC
**54**	mp-504426	$LiH_5S(NO_2)_2$	0.09	5.62	3.35E-04	1.65E-04	New FC, selected for AIMD
**55**	mp-756622	LiY_3WO_8	0.07	2.95	4.63E-04	1.65E-04	New FC
**56**	mp-850407	$LiMnP(H_6O_5)_2$	0.04	4.17	4.11E-04	1.64E-04	New FC
**57**	mp-754966	Li_2MnF_6	0.00	2.68	5.23E-04	1.62E-04	New FC
**58**	mp-759632	$Li_5Mn_3F_{14}$	0.06	1.36	5.56E-04	1.59E-04	New FC
**59**	mp-1211126	$LiSc(WO_4)_2$	0.00	3.57	4.98E-04	1.55E-04	New FC
**60**	mp-985591	Li_6PS_5Br	0.05	2.13	3.57E-04	1.53E-04	Known FC with σ of 4.42E-04, in dataset
**61**	mp-782655	$LiFeP(H_6O_5)_2$	0.04	3.64	4.23E-04	1.51E-04	New FC
**62**	mp-754749	Li_2CoOF_3	0.05	0.68	6.91E-04	1.49E-04	New FC
**63**	mp-1222601	$Li_3Al_2CoO_6$	0.03	0.83	5.69E-04	1.49E-04	New FC, selected for AIMD
**64**	mp-759827	$Li_8Co(O_2F)_2$	0.08	2.22	7.40E-04	1.46E-04	New FC
**65**	mp-759422	Li_4FeOF_5	0.08	1.55	4.29E-04	1.43E-04	New FC
**66**	mp-6247	$Na_3Li_3Fe_2F_{12}$	0.00	3.62	6.58E-04	1.42E-04	New FC
**67**	mp-754135	$Li_3Mn_2F_9$	0.07	1.79	5.19E-04	1.41E-04	New FC
**68**	mp-777589	Li_2MnF_4	0.02	4.03	7.41E-04	1.39E-04	New FC
**69**	mp-643069	Li_2HIO	0.00	4.32	1.51E-03	1.38E-04	Known FC with σ of 3.7E-03, AIMD

**70**	mp-2251	Li_3N	0.00	0.98	3.58E-04	1.37E-04	Known FC with σ of 5.74E-04, in dataset
**71**	mp-776635	Li_2FeF_5	0.01	3.66	5.80E-04	1.37E-04	New FC
**72**	mp-1114234	Na_2LiRhF_6	0.04	1.84	5.48E-04	1.36E-04	New FC
**73**	mp-643655	$LiAl_2H_6ClO_6$	0.00	4.94	3.85E-04	1.35E-04	New FC
**74**	mp-764848	$Li_{14}Mn_5F_{24}$	0.07	3.53	8.12E-04	1.33E-04	New FC
**75**	mp-561396	Li_3CrF_6	0.00	3.96	6.86E-04	1.32E-04	New FC
**76**	mp-1186148	$NaLi_5N_2$	0.08	0.56	4.95E-04	1.30E-04	New FC, selected for AIMD
**77**	mp-1311449	$Li_4Co_2OF_7$	0.04	1.41	6.05E-04	1.29E-04	New FC, selected for AIMD
**78**	mp-720554	$LiPH_4NO_3F$	0.01	5.10	2.67E-04	1.28E-04	New FC
**79**	mp-1210682	$Na_3Li_3Rh_2F_{12}$	0.00	1.99	5.73E-04	1.28E-04	New FC
**80**	mp-1210919	$LiLa(WO_4)_2$	0.00	3.68	3.24E-04	1.24E-04	New FC
**81**	mp-759420	Li_3MnF_5	0.05	3.63	7.87E-04	1.23E-04	New FC
**82**	mp-768074	$LiTaWO_6$	0.05	3.29	3.70E-04	1.21E-04	New FC
**83**	mp-770212	$Li_6Ti_2S_6O$	0.05	1.97	3.29E-04	1.18E-04	New FC, selected for AIMD
**84**	mp-697020	$LiAlSiH_2O_5$	0.01	4.81	2.92E-04	1.18E-04	New FC, selected for AIMD
**85**	mp-861557	$Li_4Mn_3OF_{11}$	0.05	1.09	5.59E-04	1.15E-04	New FC
**86**	mp-1211324	Li_7PS_6	0.03	2.08	3.13E-04	1.14E-04	Known FC with σ of 1.1E-04
**87**	mp-759784	$Li_2Bi_2P_2O_9$	0.09	3.31	1.65E-03	1.13E-04	New FC, selected for AIMD
**88**	mp-776522	$Li_4Fe_2OF_8$	0.06	0.70	4.63E-04	1.13E-04	New FC
**89**	mp-759770	$Li_7Co(OF)_3$	0.10	2.00	5.34E-04	1.13E-04	New FC
**90**	mp-770536	$Li_2VH_2OF_5$	0.06	2.52	4.89E-04	1.12E-04	New FC
**91**	mp-18925	Li_6CoO_4	0.00	2.40	9.73E-04	1.12E-04	New FC, selected for AIMD
**92**	mp-22450	Li_2PbO_3	0.00	1.04	2.46E-03	1.11E-04	New FC
**93**	mp-736	Li_3P	0.00	0.70	5.30E-04	1.11E-04	Known FC with σ of 1.00E-03, in dataset
**94**	mp-777562	$Li_{10}Mn_3F_{16}$	0.05	3.36	7.61E-04	1.10E-04	New FC
**95**	mp-989577	$LiTl_2GaF_6$	0.00	4.26	3.11E-03	1.10E-04	New FC
**96**	mp-776099	$Li_{11}Fe_6F_{29}$	0.09	3.21	4.76E-04	1.10E-04	New FC
**97**	mp-721067	$LiCaP_3H_8O_{13}$	0.04	5.22	2.58E-04	1.07E-04	New FC, selected for AIMD
**98**	mp-767402	$LiLaTi_2O_6$	0.06	1.52	1.93E-04	1.07E-04	Known FC with σ of 1.09E-03
**99**	mp-674531	Li_5GeP_3	0.01	0.52	2.63E-04	1.05E-04	New FC
**100**	mp-1176620	$LiMnF_5$	0.02	1.10	3.86E-04	1.05E-04	New FC
**101**	mp-1210997	$LiTm(WO_4)_2$	0.00	3.77	3.55E-04	1.03E-04	New FC
**102**	mp-9406	$Li_2La_2Ti_3O_{10}$	0.02	2.07	1.74E-04	1.02E-04	New FC

Table S7. Rules for elemental substitution. The process for element selection follows the principles of ionic radius and valence charge.

Original element	Substituted elements	Original element	Substituted elements	Original element	Substituted elements
**Li**	Na, K, Rb, Cs	**Na**	Li, K, Rb, Cs	**K**	Li, Na, Rb, Cs
**Rb**	Li, Na, K, Cs	**Cs**	Li, Na, K, Rb	**Be**	Mg, Ca, Sr, Ba
**Mg**	Be, Ca, Sr, Ba	**Ca**	Be, Mg, Sr, Ba	**Sr**	Be, Mg, Ca, Ba
**Ba**	Be, Mg, Ca, Sr	**Sc**	Y, Ti, Zr, V	**Ti**	Sc, Zr, Hf, V
**V**	Ti, Nb, Ta, Sr	**Cr**	Mo, W	**Mn**	Fe, Co, Tc, Re
**Fe**	Co, Mn, Cr, Ni, Ru, Rh, Pd, Pt	**Co**	Fe, Mn, Ni, Ru, Rh, Pd, Pt	**Ni**	Fe, Co, Cu, Pd, Ru, Rh, Pt
**Cu**	Ni, Zn	**Zn**	Cd, Cu	**Y**	Sc, La, Lu
**Zr**	Hf, Ti, Sc,	**Nb**	Ta, V, Mo	**Mo**	W, Cr, Nb
**Tc**	Re, Mn	**Ru**	Fe, Co, Ni, Os, Rh	**Rh**	Fe, Co, Ni, Ir, Co, Ru
**Pd**	Fe, Co, Ni, Pt, Ni, Cu	**Cd**	Zn, Hg	**Hf**	Zr, Ti
**Ta**	Nb, Mo	**W**	Mo, Cr	**Ir**	Rh, Pt
**Pt**	Pd, Ir	**La**	Ce, Pr, Nd	**Ce**	La, Nd, Sm
**Pr**	Ce, Nd, Sm	**Nd**	Pr, Sm, Gd	**Sm**	Nd, Eu, Gd
**Eu**	Sm, Gd	**Gd**	Eu, Tb	**Tb**	Gd, Dy
**Dy**	Tb, Ho	**Ho**	Dy, Er	**Er**	Ho, Tm
**Er**	Ho, Tm	**Tm**	Er, Yb	**Yb**	Tm, Lu
**Lu**	Yb, Sc, Y	**Al**	Ga, In	**Ga**	Al, In
**In**	Ga, Sn, Tl	**Sn**	Pb, In	**Pb**	Sn, Bi
**Tl**	In, Bi	**Bi**	Sb, Tl	**B**	P, S, Si
**C**	Si, Ge	**Si**	Ge, Sn	**Ge**	Si, Sn
**N**	P	**P**	N	**O**	S, Se, Te
**S**	O, Se, Te	**Se**	O, S, Te	**Te**	O, S, Se
**F**	Cl, Br, I	**Cl**	F, Br, I	**Br**	F, Cl, I
**I**	F, Cl, Br				

Table S8. Validations with experimental and AIMD measurements. Only Li_2HIO was measured using AIMD simulations. In additional to table S6, we have expanded the external validation set by incorporating eight additional experimental data points ($Li_{16.2}In_9Sn_{1.1}O_{23.8}$, $Li_{6.333}Sn_{0.333}As_{0.667}S_5I$, Li_2TaOF_5, $Li_{0.36}La_{0.56}Ti_{0.97}Ga_{0.03}O_3$, $Li_{10}SnP_2S_{11.9}Te_{0.1}$, Li_2TiF_6, Li_6AsS_5I, and $Li_{6.8}Si_{0.8}As_{0.2}S_5I$) from recently published studies.

Compound	IonNet predicted σ (S cm^{-1})	Experimental/AIMD measured σ (S cm^{-1})	Compound	IonNet predicted σ (S cm^{-1})	Experimental/AIMD measured σ (S cm^{-1})
$Li_{0.36}La_{0.56}Ti_{0.97}Ga_{0.03}O_3$	2.64×10^{-4}	4.15×10^{-3}	Li_3PS_4	3.73×10^{-4}	1.50×10^{-4}
$Li_{10}Ge(PS_6)_2$	1.34×10^{-3}	1.20×10^{-3}	$Li_{6.333}Sn_{0.333}As_{0.667}S_5I$	7.16×10^{-6}	3.04×10^{-6}
$Li_{10}Sn(PS_6)_2$	3.03×10^{-3}	4.00×10^{-3}	$Li_{6.8}Si_{0.8}As_{0.2}S_5I$	3.62×10^{-5}	1.04×10^{-2}
$Li_{10}SnP_2S_{11.9}Te_{0.1}$	3.53×10^{-4}	4.30×10^{-3}	Li_6AsS_5I	6.80×10^{-6}	1.42×10^{-5}
$Li_{16.2}In_9Sn_{1.1}O_{23.8}$	8.45×10^{-6}	2.17×10^{-4}	Li_6PS_5Br	3.57×10^{-4}	4.42×10^{-4}
Li_2HIO	1.51×10^{-3}	3.70×10^{-3}	Li_6PS_5Cl	1.43×10^{-3}	3.15×10^{-3}
Li_2TaOF_5	1.94×10^{-5}	1.08×10^{-6}	Li_6PS_5I	5.02×10^{-4}	6.30×10^{-4}
Li_2TiF_6	2.45×10^{-5}	9.69×10^{-5}	$Li_7P_3S_{11}$	2.68×10^{-4}	2.42×10^{-3}
Li_3N	3.58×10^{-4}	5.74×10^{-4}	Li_7PS_6	3.13×10^{-4}	1.10×10^{-4}
Li_3P	5.30×10^{-4}	1.00×10^{-3}	$LiLaTi_2O_6$	1.93×10^{-4}	1.09×10^{-3}

Table S9. AIMD results for candidate materials. AIMD simulations were conducted at 1200 K, 900 K, 600 K, and 450 K for 30 ps for each compound, enabling the acceleration of atomic movements and the observation of diffusion behavior within a reasonable simulation timeframe. Standard deviations (S. D.) were also estimated for diffusion coefficient (D, cm^2 s^{-1}) and σ (S cm^{-1}) at different temperatures (T, K).

Compound	T	D	D S. D.	σ	σ S. D.	Compound	T	D	D S. D.	σ	σ S. D.
Li_6CoO_4	1200	4.05×10^{-5}	2.11×10^{-9}	3.96×10^{0}	2.06×10^{-1}	$Li_5P(S_2Cl)_2$	1200	8.93×10^{-5}	2.94×10^{-9}	3.03×10^{0}	1.00×10^{-1}
	900	1.48×10^{-5}	6.46×10^{-10}	1.92×10^{0}	8.42×10^{-2}		900	4.19×10^{-5}	2.44×10^{-9}	1.90×10^{0}	1.10×10^{-1}
	600	4.62×10^{-6}	1.09×10^{-9}	9.03×10^{-1}	2.12×10^{-4}		600	1.50×10^{-6}	4.93×10^{-10}	1.02×10^{-1}	3.35×10^{-2}
	450	3.05×10^{-6}	2.90×10^{-10}	5.97×10^{-1}	5.67×10^{-5}		450	2.63×10^{-9}	3.42×10^{-12}	2.39×10^{-4}	3.09×10^{-4}
Li_2BiO_3	1200	8.09×10^{-6}	5.11×10^{-10}	3.23×10^{-2}	2.25×10^{-2}	Li_4CoOF_5	1200	4.37×10^{-5}	2.44×10^{-9}	2.40×10^{0}	1.34×10^{-1}
	900	3.35×10^{-7}	5.88×10^{-10}	1.97×10^{-2}	3.46×10^{-3}		900	1.22×10^{-5}	2.16×10^{-9}	8.96×10^{-1}	1.58×10^{-1}
	600	6.01×10^{-8}	7.25×10^{-11}	5.31×10^{-3}	6.40×10^{-3}		600	1.22×10^{-7}	5.03×10^{-11}	1.34×10^{-2}	5.52×10^{-3}
	450	5.49×10^{-9}	1.98×10^{-11}	6.46×10^{-4}	2.34×10^{-3}		450	1.51×10^{-6}	5.13×10^{-10}	2.22×10^{-1}	7.52×10^{-2}
$LiH_5S(NO_2)_2$	1200	5.70×10^{-8}	8.22×10^{-11}	3.49×10^{-4}	1.16×10^{-4}	$NaLi_5N_2$	1200	4.61×10^{-5}	5.52×10^{-10}	3.92×10^{0}	4.70×10^{-2}
	900	1.20×10^{-8}	9.75×10^{-12}	2.26×10^{-4}	1.83×10^{-4}		900	4.49×10^{-7}	1.87×10^{-9}	5.09×10^{-2}	2.12×10^{-2}
	600	8.13×10^{-9}	9.19×10^{-12}	2.29×10^{-4}	2.59×10^{-4}		600	1.53×10^{-7}	7.79×10^{-11}	2.60×10^{-2}	1.33×10^{-2}
	450	3.49×10^{-9}	4.88×10^{-12}	1.31×10^{-4}	1.83×10^{-4}		450	2.18×10^{-7}	5.05×10^{-11}	4.94×10^{-2}	1.15×10^{-2}
$Li_3Al_2CoO_6$	1200	1.36×10^{-8}	2.41×10^{-11}	6.53×10^{-4}	1.16×10^{-4}	$CsLi_2F_3$	1200	6.77×10^{-6}	3.46×10^{-10}	2.86×10^{-1}	1.46×10^{-2}
	900	9.19×10^{-9}	2.70×10^{-12}	5.87×10^{-4}	1.73×10^{-4}		900	4.86×10^{-6}	1.12×10^{-9}	2.74×10^{-1}	6.32×10^{-2}
	600	6.16×10^{-9}	5.35×10^{-12}	5.90×10^{-4}	5.13×10^{-4}		600	1.78×10^{-7}	3.19×10^{-11}	1.50×10^{-2}	2.70×10^{-3}
	450	3.14×10^{-10}	5.14×10^{-12}	4.01×10^{-5}	6.58×10^{-4}		450	1.24×10^{-8}	1.92×10^{-11}	1.40×10^{-3}	2.17×10^{-3}
$Li_6Ti_2S_6O$	1200	8.35×10^{-5}	3.57×10^{-9}	2.78×10^{0}	1.19×10^{-1}	$Li_4Co_2OF_7$	1200	2.34×10^{-6}	1.75×10^{-10}	1.08×10^{-1}	2.27×10^{-2}
	900	3.66×10^{-5}	1.65×10^{-9}	1.62×10^{0}	7.30×10^{-2}		900	1.06×10^{-8}	1.03×10^{-11}	6.50×10^{-4}	6.29×10^{-4}
	600	2.94×10^{-6}	6.14×10^{-9}	1.96×10^{-1}	4.08×10^{-1}		600	1.87×10^{-9}	1.60×10^{-11}	1.72×10^{-4}	1.47×10^{-3}
	450	1.03×10^{-8}	1.97×10^{-11}	9.09×10^{-3}	1.75×10^{-3}		450	1.40×10^{-9}	5.20×10^{-12}	1.72×10^{-4}	6.37×10^{-4}
$Li_4Bi_2O_5$	1200	2.15×10^{-5}	5.30×10^{-10}	4.64×10^{-1}	2.31×10^{-2}	$LiCaP_3H_8O_{13}$	1200	6.28×10^{-5}	9.17×10^{-9}	3.78×10^{-1}	5.52×10^{-2}
	900	2.26×10^{-7}	2.80×10^{-10}	1.31×10^{-2}	1.62×10^{-2}		900	3.68×10^{-5}	1.50×10^{-9}	2.96×10^{-1}	1.20×10^{-2}
	600	1.71×10^{-7}	2.54×10^{-11}	1.48×10^{-2}	2.20×10^{-3}		600	8.89×10^{-6}	1.31×10^{-9}	1.07×10^{-1}	1.58×10^{-2}
	450	8.83×10^{-8}	1.03×10^{-11}	1.02×10^{-2}	1.19×10^{-3}		450	8.97×10^{-7}	3.51×10^{-10}	1.44×10^{-2}	5.64×10^{-3}
$LiAlSiH_2O_5$	1200	9.01×10^{-6}	7.25×10^{-10}	1.35×10^{-1}	1.09×10^{-2}	$Li_{10}SiP_2S_{12}$	1200	4.28×10^{-5}	4.88×10^{-9}	1.34×10^{0}	1.53×10^{-1}
	900	1.97×10^{-6}	3.81×10^{-11}	3.93×10^{-2}	7.60×10^{-4}		900	3.26×10^{-5}	2.73×10^{-9}	1.36×10^{0}	1.14×10^{-1}
	600	1.45×10^{-6}	3.19×10^{-11}	4.33×10^{-2}	9.57×10^{-4}		600	9.29×10^{-6}	1.00×10^{-10}	5.82×10^{-1}	6.28×10^{-2}
	450	9.49×10^{-8}	7.12×10^{-11}	3.79×10^{-3}	2.85×10^{-3}		450	7.56×10^{-6}	5.07×10^{-10}	6.32×10^{-1}	4.24×10^{-2}
$Li_2Bi_2P_2O_9$	1200	8.05×10^{-8}	4.27×10^{-11}	1.28×10^{-3}	6.79×10^{-4}	$Li_{20}Si_3P_3S_{23}Cl$	1200	6.00×10^{-5}	2.14×10^{-9}	1.87×10^{0}	6.66×10^{-2}
	900	1.83×10^{-7}	5.59×10^{-11}	3.88×10^{-3}	1.18×10^{-3}		900	1.96×10^{-5}	6.30×10^{-10}	8.17×10^{-1}	2.62×10^{-2}
	600	2.44×10^{-8}	3.64×10^{-11}	7.75×10^{-4}	1.16×10^{-4}		600	1.25×10^{-5}	9.66×10^{-10}	7.83×10^{-1}	6.03×10^{-2}

	450	4.32×10^{-8}	1.95×10^{-11}	1.83×10^{-3}	2.94×10^{-4}		450	5.97×10^{-6}	3.40×10^{-10}	4.97×10^{-1}	2.83×10^{-2}
LiHS	1200	1.27×10^{-4}	4.44×10^{-9}	4.04×10^{0}	1.41×10^{-1}	$LiBiF_4$	1200	2.73×10^{-2}	1.18×10^{-9}	1.64×10^{-1}	2.74×10^{-2}
	900	7.57×10^{-5}	6.95×10^{-9}	3.20×10^{0}	2.94×10^{-1}		900	7.70×10^{-6}	1.19×10^{-9}	2.39×10^{-1}	3.68×10^{-2}
	600	7.76×10^{-6}	1.00×10^{-10}	4.21×10^{-1}	5.44×10^{-3}		600	1.46×10^{-6}	6.22×10^{-10}	6.80×10^{-2}	2.89×10^{-2}
	450	2.55×10^{-8}	7.70×10^{-11}	2.16×10^{-3}	6.51×10^{-3}		450	1.76×10^{-6}	1.09×10^{-10}	1.09×10^{-1}	6.77×10^{-3}
Li_2BiF_5	1200	6.39×10^{-5}	5.18×10^{-9}	1.78×10^{0}	1.44×10^{-1}	$Na_2Li_2CoO_4$	1200	1.27×10^{-5}	8.68×10^{-10}	3.82×10^{-1}	2.60×10^{-2}
	900	2.11×10^{-5}	1.43×10^{-9}	7.83×10^{-1}	5.32×10^{-2}		900	2.29×10^{-6}	1.98×10^{-10}	9.16×10^{-2}	7.93×10^{-3}
	600	6.33×10^{-6}	4.39×10^{-10}	3.53×10^{-1}	2.45×10^{-2}		600	7.41×10^{-7}	1.24×10^{-10}	4.44×10^{-2}	7.45×10^{-3}
	450	2.96×10^{-6}	2.44×10^{-10}	2.19×10^{-1}	1.81×10^{-2}		450	7.54×10^{-7}	8.06×10^{-11}	6.30×10^{-2}	6.45×10^{-3}

Data S1.
Standardized tabular dataset containing 8,750 computational samples for model training.

Data S2.
Data containing 398 experimental samples for transfer learning.
